# Identification and Analysis of Putative Homologues of Mechanosensitive Channels in Pathogenic Protozoa

**DOI:** 10.1371/journal.pone.0066068

**Published:** 2013-06-13

**Authors:** David L. Prole, Colin W. Taylor

**Affiliations:** Department of Pharmacology, University of Cambridge, Cambridge, United Kingdom; Institut national de la santé et de la recherche médicale - Institut Cochin, France

## Abstract

Mechanosensitive channels play important roles in the physiology of many organisms, and their dysfunction can affect cell survival. This suggests that they might be therapeutic targets in pathogenic organisms. Pathogenic protozoa lead to diseases such as malaria, dysentery, leishmaniasis and trypanosomiasis that are responsible for millions of deaths each year worldwide. We analyzed the genomes of pathogenic protozoa and show the existence within them of genes encoding putative homologues of mechanosensitive channels. *Entamoeba histolytica*, *Leishmania* spp., *Trypanosoma cruzi* and *Trichomonas vaginalis* have genes encoding homologues of Piezo channels, while most pathogenic protozoa have genes encoding homologues of mechanosensitive small-conductance (MscS) and K^+^-dependent (MscK) channels. In contrast, all parasites examined lack genes encoding mechanosensitive large-conductance (MscL), mini-conductance (MscM) and degenerin/epithelial Na^+^ (DEG/ENaC) channels. Multiple sequence alignments of evolutionarily distant protozoan, amoeban, plant, insect and vertebrate Piezo channel subunits define an absolutely conserved motif that may be involved in channel conductance or gating. MscS channels are not present in humans, and the sequences of protozoan and human homologues of Piezo channels differ substantially. This suggests the possibility for specific targeting of mechanosensitive channels of pathogens by therapeutic drugs.

## Introduction

Protozoan parasites are major causes of human and animal disease [1], [2]. Prominent human pathogenic protozoa include the trypanosomatid parasites *Leishmania* spp. (the cause of leishmaniasis) and *Trypanosoma* spp. (sleeping sickness, Chagas’ disease), as well as the apicomplexan parasites *Plasmodium* spp. (malaria), *Cryptosporidium* spp. (cryptosporidiosis, diarrhoea), *Toxoplasma gondii* (toxoplasmosis) and *Babesia bovis* (babesiosis). Others include *Giardia intestinalis* (giardiasis), *Entamoeba histolytica* (dysentery) and *Trichomonas vaginalis* (trichomoniasis). Collectively these parasites cause billions of infections and millions of deaths every year. Current treatments for protozoan diseases have significant side effects, they are often ineffective, and they are prone to the emergence of drug-resistant strains [3]–[5]. New therapeutic targets and drugs are needed.

Mechanosensitive channels are transmembrane proteins with pores that mediate flow of ions or osmolytes across membranes in response to mechanical stimuli. They are essential for somatosensory perception in animals [6]–[13]. In plants, they are essential for responses to osmotic shock [14], perception of touch and pressure [15]–[18], sensing of gravity [19], [20] and regulation of the volume and division of plastids [21], [22]. In fungi, they are involved in responses to osmotic shock [23]. In prokaryotes, they contribute to osmotic homeostasis [24] and resistance to ionic stress [25], [26]. Mechanically-activated currents have been detected in *Paramecium* [27]–[29] and several other free-living protozoa [30]–[35], but the proteins responsible have not been defined. Nor is it known whether mechanically-induced transmembrane current or flow of osmolytes occurs in pathogenic protozoa.

Several major types of mechanosensitive channel are known. These include the recently described Piezo channels [6], [12], [36]; degenerin/epithelial Na^+^ (DEG/ENaC) channels [8], [11], [37], [38]; some transient receptor potential (Trp) channels [39]–[44]; some two-pore K^+^ (K_2P_) channels [45]; and the osmoregulatory small-conductance (MscS), K^+^-dependent (MscK), mini-conductance (MscM) and large conductance (MscL) mechanosensitive channels found in prokaryotes, fungi, plants, and photosynthetic protists [23], [24], [46], [47]. The *mid1*-complementing activity (MCA) proteins of plants may also be mechanosensitive Ca^2+^ channels [15], [48]. We and others have previously reported the presence of genes encoding putative Trp channels [1], [49], but not K_2P_ channels [50], in the genomes of pathogenic protozoa. It is possible that some of the protozoan Trp channels are mechanosensitive, although this will require experimental analysis as many Trp channels are modulated by other stimuli [40]–[42]. Genes encoding Piezo subunits exist in the protozoan ciliates *Paramecium tetraurelia* and *Tetrahymena thermophila*, and the amoeba *Dictyostelium discoideum* [6]. However, the presence of mechanosensitive Piezo, DEG/ENaC, MCA and Msc channels in pathogenic protozoa has not been reported.

Here, we show that genes encoding homologues of mechanosensitive Piezo and MscS/MscK channel subunits exist in many of the protozoan genomes examined. Genes encoding MscS/MscK channel subunits are absent from humans, and there are substantial sequence differences between protozoan and human homologues of Piezo channel subunits. Furthermore, loss of the activity of mechanosensitive channels has profound effects on cell function [6], [12], [24], [39], suggesting that parasite-specific targeting of these channels by drugs may be a novel therapeutic strategy. Piezo channels have only recently been described and little is known about the structural basis of their function. We use comparisons of protozoan, amoeban, plant, insect and vertebrate homologues of Piezo channel subunits to identify a conserved region that may be involved in the conduction of ions, or gating, in channels formed from these subunits.

## Results and Discussion

We searched the genomes of pathogenic protozoa for genes encoding putative homologues of mechanosensitive channels. Our analyses indicate that in addition to previously described genes encoding homologues of Trp channel subunits [1], [49], the genomes of pathogenic protozoa also encode homologues of Piezo and MscS/MscK subunits (**Table 1**). Many of these putative homologues are not yet annotated in pathogen databases (http://eupathdb.org/eupathdb). Experimental studies will be required to confirm the expression and function of these proteins in parasites. In contrast, all protozoan genomes examined lack genes encoding homologues of MscL, MscM, DEG/ENaC and MCA channel subunits.

**Table 1:** Homologues of mechanosensitive channels in pathogenic protozoa.

Parasite	MscS/MscK channels	Piezo channels
*Plasmodium falciparum*	XP_001347767 (7)	NF
*Plasmodium knowlesi*	XP_002259080 (8)	NF
*Plasmodium vivax*	XP_001615208 (8)	NF
*Toxoplasma gondii*	XP_002368881 (12) XP_002370717 (2)	NF
*Cryptosporidium hominis*	XP_667697 (6) XP_667739 (6)	NF
*Cryptosporidium muris*	XP_002140152 (6) XP_002139761 (6) XP_002141174 (6)	NF
*Cryptosporidium parvum*	XP_625349 (6) XP_628630 (6)	NF
*Babesia bovis*	XP_001609879 (8) XP_001609876 (2)	NF
*Giardia intestinalis*	NF	NF
*Entamoeba histolytica*	XP_655684 (7)	XP_649449 (36) XP_655549 (36)
*Leishmania major*	XP_001687164 (2)	XP_001686914 (44) XP_001686223 (18)
*Leishmania infantum*	XP_001469960 (2)	XP_001469682 (42) XP_001468509 (18)
*Leishmania braziliensis*	XP_001569218 (2)	XP_001564414 (18) (XP_001568976)^a^ (≥29)
*Trypanosoma brucei*	XP_823127 (2)	NF
*Trypanosoma cruzi*	XP_808940 (2) XP_806638 (2)	XP_812333 (34) XP_819187 (44) XP_817508 (42) XP_820998 (42)
*Trichomonas vaginalis*	NF	XP_001319509 (42) XP_001582897 (42) XP_001581503 (29) XP_001580012 (42) XP_001305124 (42) (XP_001322153)^a^ (≥24)

Protein accession numbers for homologues are shown and the number of predicted transmembrane domains (TMDs) is indicated in parentheses following each accession number. NF denotes no homologues found.

a Incomplete sequences that are not included in Figures describing the homologues of Piezo channel subunits. Genes encoding homologues of Piezo also exist in non-pathogenic, free-living protozoa such as *P. tetraurelia* and *T. thermophila* [6], and *Naegleria gruberi* (XP_002682879), but these protozoa lack genes encoding homologues of Msc channels. The free-living choanoflagellate, *Monosiga brevicollis*, has genes encoding homologues of Piezo (XP_001743938 and XP_001743848; incomplete sequences) and MscS (XP_001744510, XP_001748077 and XP_001745172).

### Homologues of Piezo Channels

**Figure 1:**
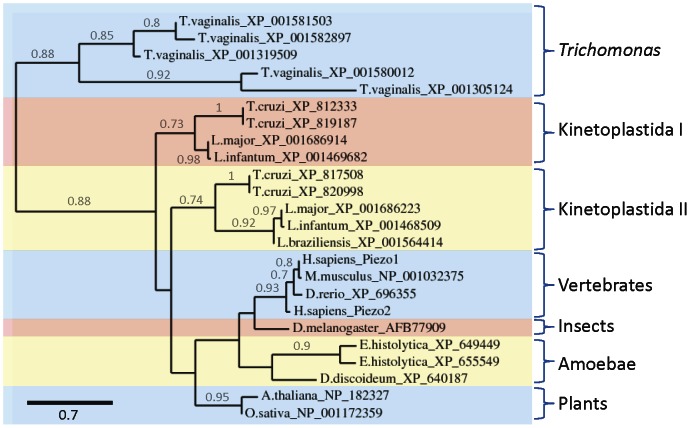
Homologues of Piezo channel subunits in pathogenic protozoa. Phylogram showing the relationship between homologues of Piezo channel subunits (*see Methods*: based on 63 high-confidence positions from a multiple sequence alignment; gamma shape parameter 1.974; proportion of invariant sites 0.085). Homologues from different groups of organisms are indicated, along with the two phylogenetically distinct groups of homologues in trypanosomatid parasites. Branch length scale bar (amino acid substitutions per site) and branch support values >0.5 are shown. Protozoan homologues with incomplete sequences (*see* *Table 1*) are not shown.

**Figure 2:**
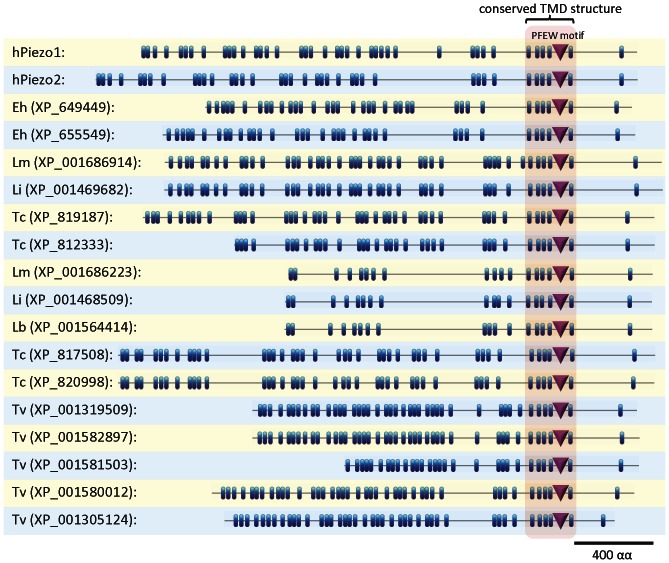
Conserved domains in homologues of Piezo channel subunits. Locations of predicted transmembrane domains (TMDs) and conserved motifs in the homologues of Piezo channel subunits from humans and pathogenic protozoa. Horizontal lines depict the length of each protein, while predicted TMDs are indicated by blue bars. The positions of TMDs along the length of each protein are shown to scale. A conserved arrangement of five TMDs near the C-terminal end of each protein is indicated by red shading. This region contains the conserved domain pfam12166 (Conserved Domains Database, NCBI). The conserved PFEW motif is indicated by a magenta triangle. Protozoan homologues with incomplete sequences (*see* *Table 1*) are not shown. Topologies were drawn using MyDomains (Swiss Institute of Bioinformatics; http://prosite.expasy.org/mydomains). Abbreviations are as follows: h, human; Eh, *E. histolytica*; Lm, *L. major*; Li, *L. infantum*; Lb, *L. braziliensis*; Tc, *T.cruzi*; Tv, *T. vaginalis*.

Piezo proteins were recently described as the pore-forming subunits of mechanosensitive channels [36]. Piezo subunits multimerize, probably as tetramers [36], to form channels that conduct both monovalent and divalent cations. They have modest selectivity for Ca^2+^ [6], [36]. They are large proteins (∼1500–4700 residues) with many predicted transmembrane domains (TMDs) (∼18–44) distributed throughout their sequences [6]. Their existence has been reported in vertebrates, plants, nematodes, insects, amoebae and free-living ciliates, but they are absent from many fungi [6], [51], [52]. We found that genes encoding homologues of Piezo are present in the genomes of *E. histolytica*, *Leishmania* spp., *Trypanosoma cruzi* and *T. vaginalis*, but they are absent from *Trypanosoma brucei*, *G. intestinalis* and the apicomplexan parasites examined (**Table 1**). Two phylogenetically distinct groups of Piezo homologues exist in the trypanosomatid parasites and these are more divergent than the two human subtypes of Piezo (**Figure 1**). This suggests that multiple subtypes of Piezo may have evolved separately in trypanosomatid parasites and humans. The mammalian Piezo subunits and their protozoan homologues range in size from 1496–2752 residues, but they are similar in having many predicted TMDs (**Figure 2**).

**Figure 3:**
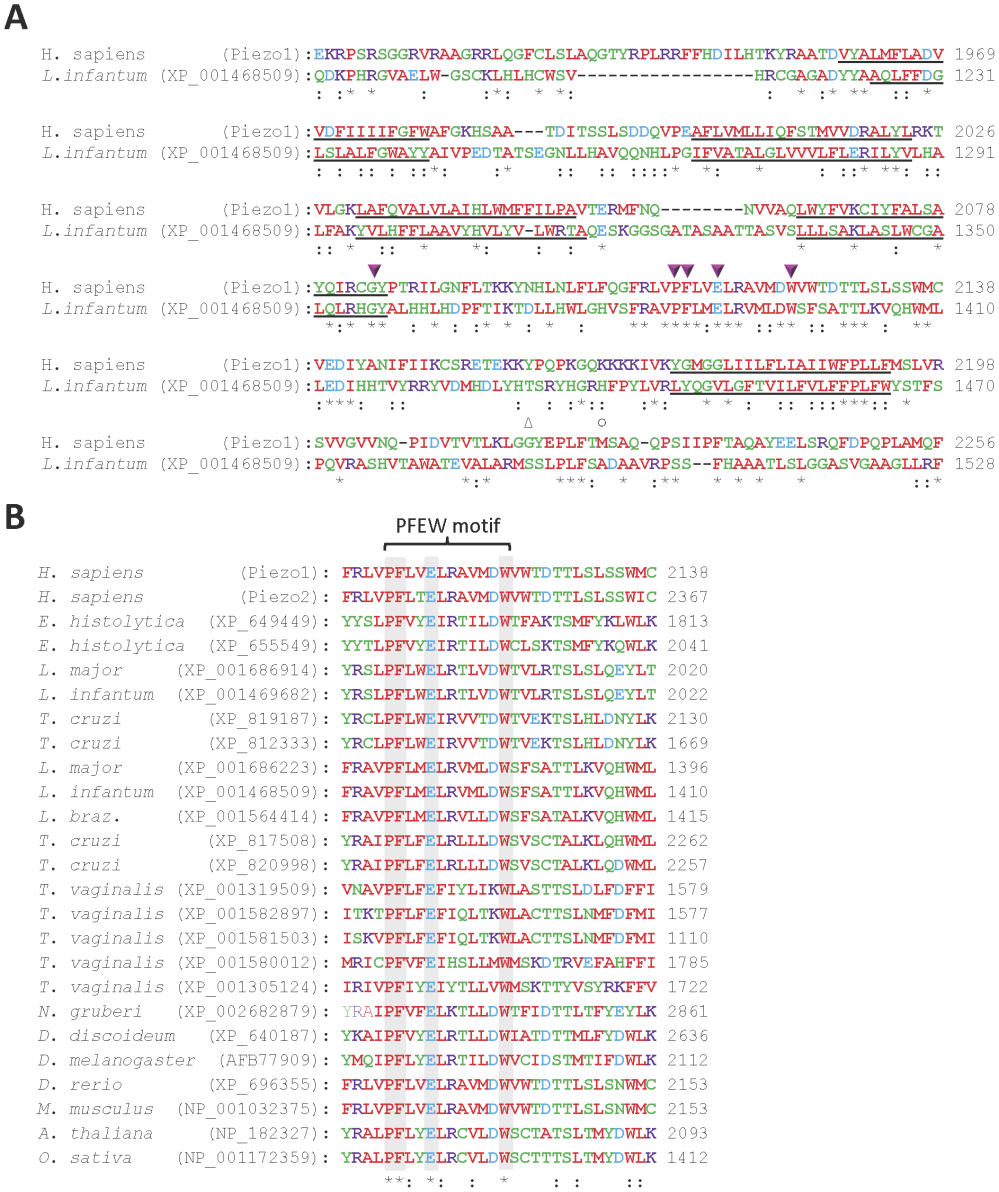
Conserved residues in homologues of Piezo channel subunits. (**A**) Alignment of human Piezo1 with a homologue in *L. infantum*. Predicted TMDs are underlined. Asterisks below the alignment indicate residues conserved in human Piezo1 and the homologue shown from *L. infantum*. Colons indicate residues with highly similar properties. Magenta triangles indicate residues, including the PFEW motif, that are conserved absolutely in predicted homologues of Piezo in all organisms examined (*Figure S1*). An open circle above the alignment indicates a residue in human Piezo1 that is mutated in familial xerocytosis and alters channel gating [53]. An open triangle above the alignment indicates a position at which the introduction of a stop codon alters gating [53]. (**B**) Multiple sequence alignment of predicted Piezo homologues from protozoa, humans, mouse, *Dictyostelium discoideum*, *Drosophila melanogaster*, *Danio rerio*, *Arabidopsis thaliana* and *Oryza sativa*. The absolutely conserved PFEW motif is indicated and residues comprising this motif in each protein are shaded. Asterisks indicate absolutely conserved residues, while colons indicate residues with highly similar properties. Protozoan homologues with incomplete sequences (*see* *Table 1*) are not shown.

The structural basis of function in Piezo channels is almost completely unknown. Neither the pore-forming region nor the mechanosensing region of Piezo channels has been defined. We therefore sought to identify residues that are conserved between known Piezo channel subunits and the newly identified and evolutionarily distant protozoan proteins. This might identify residues with conserved functions within Piezo channels. When the sequences of Piezo homologues from protozoa, vertebrates, amoebae, insects and plants are aligned, only five residues are conserved in all proteins. These all lie within a relatively small region of each protein, near their C-terminal ends (red-shaded region in **Figure 2**, and **Figure S1**). Four of the absolutely conserved residues lie within a single motif, PF(X_2_)E(X_6_)W, henceforth termed PFEW. This is shown in a representative alignment of human Piezo with a homologue from *L. infantum* (**Figure 3A**) and in a multiple sequence alignment of homologues from diverse organisms (**Figure 3B**). Pore-lining TMDs and selectivity filters are often the most highly conserved regions in ion channels [14], [53], [54]. We therefore aligned the predicted TMDs from each protein to identify regions with conserved arrangements of TMDs (**Figure 2**). The PFEW motif in each protein is surrounded by five TMDs, whose arrangement is absolutely conserved between homologues of Piezo (**Figure 2** and **Figure 3A**). In contrast, the arrangements of TMDs near their N-terminal ends differ substantially (**Figure 2**). The absolute conservation of residues and arrangement of TMDs suggest that the region containing the PFEW motif is likely to be involved in the primary functions of Piezo channels, namely ion conduction and mechanosensitive gating. A mutation just outside this conserved region in human Piezo1 (**Figure 3A**) occurs in familial xerocytosis and alters channel gating [55]. In addition, human Piezo1 truncated just after this conserved region is functional, albeit with altered gating [53]. The selectivity filters of ion channels are often comprised of re-entrant pore loops, which in Ca^2+^-permeable channels contain conserved acidic residues that bind cations [56], [57]. Hence, we speculate that the conserved region of Piezo channel subunits identified in this study, containing the PFEW motif and surrounding TMDs, may be the pore-forming region. This will require experimental investigation.

### Homologues of Msc Channels

**Figure 4:**
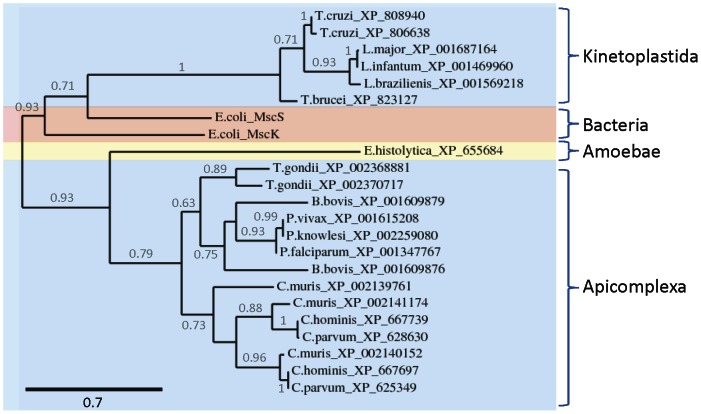
Homologues of MscS and MscK channel subunits in pathogenic protozoa. Phylogram showing the relationship between bacterial and protozoan homologues of MscS and MscK channel subunits (*see Methods*: based on 113 high-confidence positions from a multiple sequence alignment; gamma shape parameter 3.83; proportion of invariant sites 0). Homologues from different groups of organisms are indicated. Branch length scale bar (amino acid substitutions per site) and branch support values >0.5 are shown.

**Figure 5:**
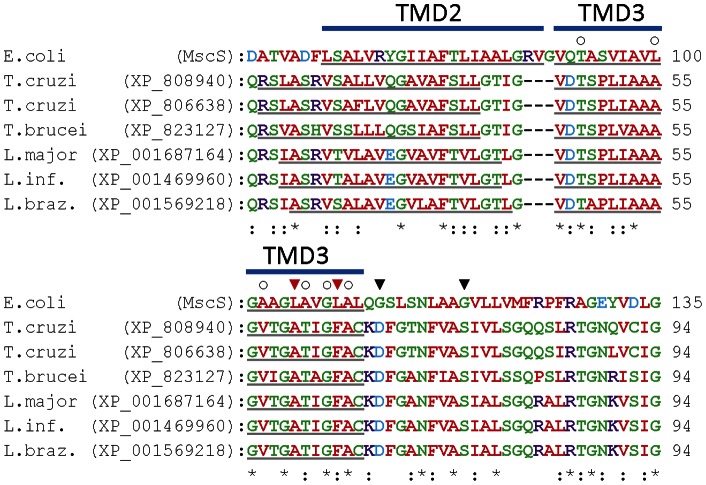
The transmembrane regions of protozoan and bacterial MscS homologues have similar sequences. Multiple sequence alignment of MscS homologues from *Escherichia coli* and trypanosomatid parasites. The TMD2 and TMD3 regions of MscS are indicated by bars above the alignment, and the predicted TMDs of individual proteins are underlined. Asterisks below the alignment indicate positions that have a single fully conserved residue, while colons below the alignment indicate positions that have residues with highly similar properties. Black triangles above the alignment indicate two glycine residues in MscS (G113 and G121) that may form gating hinges [24]. Red triangles above the alignment indicate two leucine residues in MscS (L105 and L109) that may occlude the pore in the closed state [24]. Open circles above the alignment indicate residues in MscS or the related MscK, which when mutated lead to a gain-of-function phenotype [66].

Mechanosensitive MscS, MscK, MscM and MscL channel subunits share some sequence similarity, but the resulting channels have different structures, subunit stoichiometries, gating mechanisms and biophysical properties [24], [46]. They are characteristically present in organisms with cell walls, including prokaryotes, fungi, plants and photosynthetic protists, but have not been reported in animals [23], [24], [46], [58]–[61]. Our analyses indicate the presence of genes encoding homologues of MscS/MscK subunits in all pathogenic protozoa examined except *G. intestinalis* and *T. vaginalis* (**Table 1**). Several protozoa have multiple genes encoding homologues of MscS/MscK (**Table 1**), but genes encoding homologues of MscL and MscM subunits are not found. Homologues of MscS/MscK in trypanosomatid and apicomplexan parasites are phylogenetically distinct (**Figure 4**). We chose to analyze further the MscS/MscK homologues in trypanosomatid parasites, because they showed greatest similarity to the extensively studied bacterial MscS/MscK channels. Multiple sequence alignments show that these homologues have TMDs with sequences and boundaries that are similar to TMD2/3 of MscS subunits from *E. coli* (**Figure 5**). In heptameric MscS channels, these residues line the pore (TMD3) and are involved directly in sensing membrane tension and voltage (TMD2) [46], [62]–[64]. Bacterial MscS channels contain glycine residues that may act as gating hinges (G113 and G121 in MscS) and leucine residues that may occlude the pore in the closed state (L105 and L109 in MscS) (**Figure 5**) [24], [62], [65]. Like MscK channels [24], homologues of MscS/MscK in trypanosomatid parasites lack these glycine and leucine residues, but do have several glycine and alanine residues in their putative pore-lining TMD (corresponding to TMD3 of MscS; **Figure 5**). Equivalent residues in MscS are important for gating [65]. Homologues of MscS/MscK in trypanosomatid parasites also differ from bacterial MscS at several other positions within TMD3 that are involved in channel activity (**Figure 5**) [66]. Overall, these sequence analyses suggest that the protozoan homologues may form mechanically-activated channels with gating characteristics that differ from MscS/MscK channels in bacteria. This will require experimental analysis.

### Potential Physiological Functions and Therapeutic Targeting of Mechanosensitive Channels in Protozoa

Mechanically-activated currents are present in many diverse organisms [24], [37], [46], [51], [67], [68]. They have been observed in motile, free-living protozoan ciliates such as *Paramecium* [27]–[29], [69], *Stentor coeruleus* [31], *Vorticella convallaria* [32], *Euplotes vannus* [33] and *Stylonychia mytilus* [30], [34], [35], and in the photosynthetic protists *Chlamydomonas* [70] and *Euglena gracilis* [71]. Genes encoding homologues of Piezo exist in the free-living ciliates *P. tetraurelia* and *T. thermophila* [6] and in the free-living flagellate *Naegleria gruberi* (**Table 1**), but these organisms lack genes encoding homologues of Msc channels. The free-living choanoflagellate *Monosiga brevicollis*, the closest unicellular relative of animals, has genes encoding homologues of Piezo and MscS (**Table 1**). These observations suggest that mechanosensitive channels are not exclusive adaptations to parasitic life cycles of protozoa, and that genes encoding Msc channels may have been lost from the genomes of animals after the divergence of animals and choanoflagellates. The identification of genes encoding putative Msc channel subunits in apicomplexans, and putative Piezo channel subunits in excavates (*T. vaginalis* and the kinetoplastid parasites), extends the evolutionary diversity of these proteins and suggests that mechanosensitive channels may have widespread roles in eukaryotes.

Many pathogenic protozoa are motile, they have varied lifecycles, and often invade host cells [2], [72]. These events likely involve physiological responses to mechanical stimuli. Like prokaryotic MscS/MscK channels, protozoan homologues may conduct osmolytes [24], [46], or allow adaptation to high concentrations of K^+^ [24]–[26], [46], [73] after invasion of host cells. They may be involved in sensory perception [6]–[13], [15]–[18], sensing of gravity [19], [20], [30], [71], [74], [75], or regulation of intracellular organelles [21], [22], [76]. They may reside within the plasma membrane, or like mechanosensitive Trp channels [43], [77], [78], MscS channels in yeast [23], and a variety of channels in other organisms [40], [79], they may function in the membranes of intracellular organelles where changes in membrane tension also occur [80]–[83]. Mammalian Piezo channels are also present within intracellular membranes [6], suggesting additional intracellular roles. Like K^+^ channels in *P. falciparum* [84], mechanosensitive channels in protozoa may be trafficked to the host cell membrane. The multiple homologues of mechanosensitive channels in many protozoa (**Table 1**) suggest a variety of functions. In *Paramecium*, mechanically-activated currents are comprised of distinct K^+^ and Ca^2+^ conductances [85]. Several mechanosensitive channels are also present in *E. coli*, each with a different threshold for activation [25], [26], [86]–[89]. By analogy, the multiple homologues in protozoa may serve different functions, or have graded sensitivity to stimuli. Experimental analysis will be required to define their cellular locations and functions.

Mechanosensitive channels are critical for cellular homeostasis and signal transduction in many organisms, and disruption of their function can reduce cell viability. For example, disruption of MscS and MscL channels in *E. coli* makes osmotic stress lethal [25], [90]. Reduced expression of the MscS homologue in *T. brucei* impairs growth of the differentiated form of the parasite, and heat shock increases levels of mRNA encoding this homologue in the procyclic form (TriTrypDB; http://tritrypdb.org/tritrypdb; gene identifier Tb927.10.9030) [91]. Ruthenium red [6], [36], Gd^3+^ [6], the tarantula toxin GsMTx4 [92] and extracellular divalent cations [36] inhibit Piezo channels. Furthermore, GsMTx4 acts at the extracellular surface [92], suggesting that targetable epitopes may be accessible to drugs if parasite homologues of Piezo reside within the plasma membrane. Protozoan homologues of Trp channels also have potential as drug targets [1], [49]. While the human genome encodes two Piezo, nine DEG/ENaC, several mechanosensitive K_2P_ and more than 30 Trp channel subunits [6], [37], [54], [55], [93], the genomes of pathogenic protozoa each contain only a small number of genes encoding homologues of mechanosensitive channel subunits (**Table 1**) [1]. This suggests a lack of redundancy amongst mechanosensitive channels in pathogenic protozoa. These observations, together with the absence of Msc channels in humans, suggest that protozoan mechanosensitive channels are potential drug targets.

This study presents the opportunity for cloning and functional characterization of mechanosensitive channels in pathogenic protozoa, and suggests that drugs targeted against these channels could be new treatments for disease.

## Materials and Methods

### Genome Analysis, Sequence Alignments and Topology Analysis

Analyses of genomes, sequence alignments and topology analysis were conducted as reported previously [1], [50]. BLASTP and TBLASTN searches of protozoan genomes were carried out against the National Center for Biotechnology (NCBI) genomic protein databases. BLAST searches of the genome of *M. brevicollis* [94] were made using the Genome Portal of the Department of Energy Joint Genome Institute (http://genome.jgi-psf.org) [95]. In multiple sequence alignments (ClustalW2.1), residues with highly similar properties are defined as those scoring >0.5 in the Gonnet PAM 250 matrix, and physiochemical residue colours are shown. BLAST analyses were carried out using sequences of the following mechanosensitive channels (protein accession number in parentheses): human Piezo1 (NP_001136336); human Piezo2 (NP_071351); ENaC1a (NP_001029); MscS (AC75961); MscK (NP_414998); MscM (AAC73678); MscL (AAA58088); *Schizosaccharomyces pombe* MsY1 (NP_587894) and MsY2 (NP_594520); and *A. thaliana* MCA1 (AEE86590) and MCA2 (AEC06682). The sequences of parasite homologues were then used in further BLAST searches to identify additional homologues. Default BLAST parameters for assessing statistical significance and for filtering were used in all cases (*ie*. an Expect threshold of 10, and SEG filtering). Several procedures ensured that hits were probable mechanosensitive channel homologues. Firstly, the occurrence of multiple putative TMDs was confirmed using TOPCONS [96]. Secondly, reciprocal BLASTP searches (non-redundant protein database at NCBI) were undertaken, using identified parasite hits as bait, and only proteins that gave the original mammalian protein family as hits were analyzed further. Thirdly, conserved domains were identified using the Conserved Domains Database (NCBI). For phylogenetic analysis, multiple sequence alignments were constructed with MUSCLE v3.7 using default parameters, except for the long Piezo proteins for which alignments were made using ClustalW2.1, with default parameters. After use of GBLOCKS at low stringency to remove regions of low confidence, and removal of gaps, maximum likelihood analysis was undertaken using PhyML v3.0 (WAG substitution model; 4 substitution rate categories; default estimated gamma distribution parameters; default estimated proportions of invariable sites; 100 bootstrapped data sets). Phylogenetic trees are shown using TreeDyn (v198.3). MUSCLE, GBLOCKS, PhyML and TreeDyn are all functions of Phylogeny.fr (http://www.phylogeny.fr) [97].

## Supporting Information

Figure S1 **Identification of conserved residues in homologues of Piezo channel subunits.** Multiple sequence alignment of predicted homologues of Piezo channel subunits from protozoa, vertebrates, *Naegleria gruberi*, *D. discoideum*, *D. rerio*, *D. melanogaster*, *A. thaliana* and *O. sativa*. The absolutely conserved PFEW motif and the surrounding five conserved predicted TMDs in human Piezo1 are highlighted with yellow shading. Open circles above the alignment indicate residues in human Piezo1 that are mutated in familial xerocytosis and alter channel gating [53]. An open triangle above the alignment indicates a position at which the introduction of a stop codon alters gating [53]. A filled circle above the alignment indicates the position of a single amino acid deletion in human Piezo2 that occurs in a subtype of Distal Arthrogryposis (E2727del) and alters channel gating [98]. Asterisks indicate absolutely conserved residues, while colons indicate residues with highly similar properties. Protozoan homologues with incomplete sequences (*see* *Table 1*) are not shown.
(DOCX)
