# Supplementary material for: Identification and Analysis of Putative Homologues of Mechanosensitive Channels in Pathogenic Protozoa
Source: PLoS One. 2013 Jun 13;8(6):e66068. doi: 10.1371/journal.pone.0066068 (PMC3681921; doi:10.1371/journal.pone.0066068)
Supplement: Figure S1 — Identification of conserved residues in homologues of Piezo channel subunits. Multiple sequence alignment of predicted homologues of Piezo channel subunits from protozoa, vertebrates, Naegleria gruberi, D. discoideum, D. rerio, D. melanogaster, A. thaliana and O. sativa. The absolutely conserved PFEW motif and the surrounding five conserved predicted TMDs in human Piezo1 are highlighted with yellow shading. Open circles above the alignment indicate residues in human Piezo1 that are mutated in familial xerocytosis and alter channel gating [53]. An open triangle above the alignment indicates a position at which the introduction of a stop codon alters gating [53]. A filled circle above the alignment indicates the position of a single amino acid deletion in human Piezo2 that occurs in a subtype of Distal Arthrogryposis (E2727del) and alters channel gating [98]. Asterisks indicate absolutely conserved residues, while colons indicate residues with highly similar properties. Protozoan homologues with incomplete sequences (see Table 1 ) are not shown. (DOCX) [file pone.0066068.s001.docx]

**Figure S1. Identification of conserved residues in homologues of Piezo channel subunits.**

hPiezo1 (NP_001136336): ------------------------------------------------------------

hPiezo2 (NP_071351): ------------------------------------------------------------

E.histolytica (XP_649449): ------------------------------------------------------------

E.histolytica (XP_655549): ------------------------------------------------------------

L.major (XP_001686914): ------------------------------------------------------------

L.infantum (XP_001469682): ------------------------------------------------------------

T.cruzi (XP_819187): ------------------------------------------------------------

T.cruzi (XP_812333): ------------------------------------------------------------

L.major (XP_001686223): ------------------------------------------------------------

L.infantum (XP_001468509): ------------------------------------------------------------

L.braziliensis (XP_001564414): ------------------------------------------------------------

T.cruzi (XP_817508): ------------------------------------------------------------

T.cruzi (XP_820998): ------------------------------------------------------------

T.vaginalis (XP_001319509): ------------------------------------------------------------

T.vaginalis (XP_001582897): ------------------------------------------------------------

T.vaginalis (XP_001581503): ------------------------------------------------------------

T.vaginalis (XP_001580012): ------------------------------------------------------------

T.vaginalis (XP_001305124): ------------------------------------------------------------

N.gruberi (XP_002682879): MNTITTDNNNDNQRRRSSMLGNSADETTPSYLTFNYNNNNNNNNNDNNIINNNNDISPSL

D.discoideum (XP_640187): ------------------------------------------------------------

D.rerio (XP_696355): ------------------------------------------------------------

D.melanogaster (AFB77909): ------------------------------------------------------------

M.musculus (NP_001032375): ------------------------------------------------------------

R.norvegicus (NP_001070668): ------------------------------------------------------------

B.taurus (XP_002694905): ------------------------------------------------------------

C.lupus (XP_546782): ------------------------------------------------------------

A.thaliana (NP_182327): ------------------------------------------------------------

O.sativa (NP_001172359): ------------------------------------------------------------

hPiezo1 (NP_001136336): -----------------MEP-----HV---------------------------------

hPiezo2 (NP_071351): ------------------------------------------------------------

E.histolytica (XP_649449): ------------------------------------------------------------

E.histolytica (XP_655549): ------------------------------------------------------------

L.major (XP_001686914): -------------MSSSREFITTGGGPAPIS-----------------RRCVAT------

L.infantum (XP_001469682): -------------MSSSHEFITMDGGPAPIS-----------------RRRVAT------

T.cruzi (XP_819187): -------------------------MIMAVE-----------------RQCVDS------

T.cruzi (XP_812333): ------------------------------------------------------------

L.major (XP_001686223): ------------------------------------------------------------

L.infantum (XP_001468509): ------------------------------------------------------------

L.braziliensis (XP_001564414): ------------------------------------------------------------

T.cruzi (XP_817508): -----------------------MPWSHGVS-----------------RPCTPH------

T.cruzi (XP_820998): -----------------------MPWSHGLS-----------------RPFTPH------

T.vaginalis (XP_001319509): -------MNQETELREVMDSTLIQ-PSED-------------------------------

T.vaginalis (XP_001582897): -------MEQPAEPHVVQKETLVS-ESTS-------------------------------

T.vaginalis (XP_001581503): ------------------------------------------------------------

T.vaginalis (XP_001580012): ---MSSDSSDEYEVSSEPSSTTVT-GPDDGNPE---------------------------

T.vaginalis (XP_001305124): ------MEDSEYELSSEPSTTTAS-GDEVVDPD---------------------------

N.gruberi (XP_002682879): LDDDLIMDDSKSDILSTIQTTTSGGGIRGGGDIDIADDQSSIEQSQTSFNALDQMTNLTD

D.discoideum (XP_640187): ------------------------------------------------------------

D.rerio (XP_696355): ------------------------------------------------------------

D.melanogaster (AFB77909): ------------------------------------------------------------

M.musculus (NP_001032375): ------------------------------------------------------------

R.norvegicus (NP_001070668): ------------------------------------------------------------

B.taurus (XP_002694905): -------------MTSVLAL-----GA-G---------------------------GSSA

C.lupus (XP_546782): -------------MTVALQP-----PP-QGHP----------------FSCI--LSGTSQ

A.thaliana (NP_182327): ------------------------------------------------------------

O.sativa (NP_001172359): ------------------------------------------------------------

hPiezo1 (NP_001136336): ----------LGAVLYWLLLPCALLAACLLRFSG-----------LS-----LVY---LL

hPiezo2 (NP_071351): -M----ASEVVCGLIFRLLLPICLAVACAFRYNG-----------LS-----FVY---LI

E.histolytica (XP_649449): ------------------------------------------------------------

E.histolytica (XP_655549): ------------------------------------------------------------

L.major (XP_001686914): ----------LHVLFFVLATIS----------------------AVS-------------

L.infantum (XP_001469682): ----------LHVLFFALATIS----------------------AVS-------------

T.cruzi (XP_819187): ----------FAYFLYAFNTFHLCLCLCCTRNAHLFFFSCNDSFTLFMRVYLFVMVIAFF

T.cruzi (XP_812333): ------------------------------------------------------------

L.major (XP_001686223): ------------------------------------------------------------

L.infantum (XP_001468509): ------------------------------------------------------------

L.braziliensis (XP_001564414): ------------------------------------------------------------

T.cruzi (XP_817508): ----------LQCVILSIVYL---------------------------------------

T.cruzi (XP_820998): ----------LQCVILSIVYL---------------------------------------

T.vaginalis (XP_001319509): ------------------------------------------------------------

T.vaginalis (XP_001582897): ------------------------------------------------------------

T.vaginalis (XP_001581503): ------------------------------------------------------------

T.vaginalis (XP_001580012): ------------------------------------------------------------

T.vaginalis (XP_001305124): ------------------------------------------------------------

N.gruberi (XP_002682879): EVIGKTLVLEIISIIICVLFYLSLLVSFAIRPSF-----------IN-----LTY---ML

D.discoideum (XP_640187): -MIGYF--------FVSLIYPFSLICSSVFRSNV-----------VS-----YIY---FI

D.rerio (XP_696355): -M----ELQVVCGLLYCCLLPIFLLAACIFRYNA-----------LS-----LVY---LL

D.melanogaster (AFB77909): -M----VFSYACMVLQRIVVPAVLVLAALMRPVG-----------IS-----FVY---LL

M.musculus (NP_001032375): -M----EPHVLGAGLYWLLLPCTLLAASLLRFNA-----------LS-----LVY---LL

R.norvegicus (NP_001070668): -M----EPHVLGAGLYWLLLPCTLLAASLLRFNA-----------LS-----LVY---LL

B.taurus (XP_002694905): ER----VL--LHVQHEGPLLSVNRACPCLFRVNA-----------LS-----LVY---LL

C.lupus (XP_546782): RF----SLKRMKFQVPPLEAGSGLMPACLFRYNA-----------LS-----LVY---LL

A.thaliana (NP_182327): ----------MASFLVGFLLPSLLLAAALINWSV-----------IS-----FLD---LI

O.sativa (NP_001172359): ------------------------------------------------------------

hPiezo1 (NP_001136336): FLLLLPWFPGPTRCGLQGHTGRLLRA---------------------LLGLS--------

hPiezo2 (NP_071351): YLLLIPLFSEPTKTTMQGHTGRLLKSL-------CFISL-------S-------------

E.histolytica (XP_649449): ------------------------------------------------------------

E.histolytica (XP_655549): ------------------------------------------------------------

L.major (XP_001686914): -LRAPIWLIGGLVYCSK------------------HRNALTGQHLFPLRLWQVLLASLVT

L.infantum (XP_001469682): -LRAPMWLIGGLVYCSK------------------HRSALIGQHLFPLRLWQVLLASLVA

T.cruzi (XP_819187): FVFIFFWGIVLLVFSIKLGLAKMIHPFALVFSGLLTSSAMLGGHLPAAVLLIY--MMLLR

T.cruzi (XP_812333): ------------------------------------------------------------

L.major (XP_001686223): ------------------------------------------------------------

L.infantum (XP_001468509): ------------------------------------------------------------

L.braziliensis (XP_001564414): ------------------------------------------------------------

T.cruzi (XP_817508): --------------------------------HSLFIPALLGQSIIATLLLVL-------

T.cruzi (XP_820998): --------------------------------HSLFIPALLGQSIIATLLLVL-------

T.vaginalis (XP_001319509): ------------------------------------------------------------

T.vaginalis (XP_001582897): ------------------------------------------------------------

T.vaginalis (XP_001581503): ------------------------------------------------------------

T.vaginalis (XP_001580012): ------------------------------------------------------------

T.vaginalis (XP_001305124): ------------------------------------------------------------

N.gruberi (XP_002682879): IFFLHNLTPSFYPYFQKVGINRIWMMIP----RFSTLSI-------SLLVSICYFIGTI-

D.discoideum (XP_640187): FFLLSILCLPHKSLILKNKISK---TLP----I-----I-------TLVLSMFSLILQLL

D.rerio (XP_696355): YLLLLPWFQWPNKHTLRGHTGCYIKAL-------FSTS----------------------

D.melanogaster (AFB77909): MFFVSPFVPLATRRNFKGSVTAFFIIL-------LTLST---------------------

M.musculus (NP_001032375): FLLLLPWLPGPSRHSIPGHTGRLLRAL-------LCLS----------------------

R.norvegicus (NP_001070668): FLLLLPWLPGPSRHSIPGHTGRLLRAL-------LCLS----------------------

B.taurus (XP_002694905): FLLLLPWFPGPSRHSIRDLCCDLILQG-------RCRVP-------PAMGWATFLPGSIH

C.lupus (XP_546782): FLLLLPWFPGPCRRGFPGHTRRLLRA---------------------LLVFS--------

A.thaliana (NP_182327): AFLLVHYIAPEIGYRFQRR---HWLLWP----IFIFSFA-------VFLAQVVYLVIW--

O.sativa (NP_001172359): ------------------------------------------------------------

hPiezo1 (NP_001136336): ------------------------------------------------------------

hPiezo2 (NP_071351): ------------------------------------------------------------

E.histolytica (XP_649449): ------------------------------------------------------------

E.histolytica (XP_655549): ------------------------------------------------------------

L.major (XP_001686914): SAVC--------------------------------------------------------

L.infantum (XP_001469682): STVC--------------------------------------------------------

T.cruzi (XP_819187): NPKCAT-----------------------------------------SPIVLVLAFLFAV

T.cruzi (XP_812333): ------------------------------------------------------------

L.major (XP_001686223): ------------------------------------------------------------

L.infantum (XP_001468509): ------------------------------------------------------------

L.braziliensis (XP_001564414): ------------------------------------------------------------

T.cruzi (XP_817508): NLVCAVRYENHRWRHLTLHSSDNWNDAISARLIPVRK-VSLLSFLLPSQLLLYVSLVFSC

T.cruzi (XP_820998): NLVCAVRYENHRWRHLTLHSSDNWNDAISARLIPVRK-VSLLSFLLPSQLLLYVSLVFSC

T.vaginalis (XP_001319509): ------------------------------------------------------------

T.vaginalis (XP_001582897): ------------------------------------------------------------

T.vaginalis (XP_001581503): ------------------------------------------------------------

T.vaginalis (XP_001580012): ------------------------------------------------------------

T.vaginalis (XP_001305124): ------------------------------------------------------------

N.gruberi (XP_002682879): --VVSVVSINDNYLT-------DVLTDIGFVSLKGKDISFILIHVIPDALVIIFS--FTC

D.discoideum (XP_640187): VNVVKVFQEQDELSV-------NILTAFGFYKYNSF--WIVFRNVLPDVIVFVIS-----

D.rerio (XP_696355): ------------------------------------------------------------

D.melanogaster (AFB77909): ------------------------------------------------------------

M.musculus (NP_001032375): ------------------------------------------------------------

R.norvegicus (NP_001070668): ------------------------------------------------------------

B.taurus (XP_002694905): --VCSVL------CG-------AVLSSDHL-------------------VVREPS--SIS

C.lupus (XP_546782): ------------------------------------------------------------

A.thaliana (NP_182327): ---AALGQDWDTPDT-------GWMRVIGFMILKS-------------------------

O.sativa (NP_001172359): ------------------------------------------------------------

hPiezo1 (NP_001136336): LLFLVA------------------------HLALQICLHI--------------------

hPiezo2 (NP_071351): --FLLL------------------------HIIFHITLVSL-------------------

E.histolytica (XP_649449): ------------------------------------------------------------

E.histolytica (XP_655549): ------------------------------------------------------------

L.major (XP_001686914): -----SLVANLVTTLQ---RNTAWVQKAVWRTLYPLW----------------ALLGVQQ

L.infantum (XP_001469682): -----SLVANLVTTLQ---YNTAWVQKAVWRTLYPLW----------------ALLGVQQ

T.cruzi (XP_819187): LQLLSTLIVNLLVAFSPSTRGGLFVHHRVLIESFGIY---------------EAGPGVKA

T.cruzi (XP_812333): ------------------------------------------------------------

L.major (XP_001686223): ------------------------------------------------------------

L.infantum (XP_001468509): ------------------------------------------------------------

L.braziliensis (XP_001564414): ------------------------------------------------------------

T.cruzi (XP_817508): LQLFLAATLGWLLGL-------SFMQQ-GWPK--------------------AVLNSLGV

T.cruzi (XP_820998): LQLFLAATLGWLLGL-------SFMQQ-GWPK--------------------AVLNSLGV

T.vaginalis (XP_001319509): ------------------------------------------------------------

T.vaginalis (XP_001582897): ------------------------------------------------------------

T.vaginalis (XP_001581503): ------------------------------------------------------------

T.vaginalis (XP_001580012): ------------------------------------------------------------

T.vaginalis (XP_001305124): ------------------------------------------------------------

N.gruberi (XP_002682879): FIFLSV---LWRRRLKLEKEGVSLKIKKVTPSTFKS---SLINMSGKVETVKSTDTGITT

D.discoideum (XP_640187): -LFTII---LWFKNLVYPAS---INIKDSLKKT--T---SLNNV-DQILNSPYVHGGSGN

D.rerio (XP_696355): LIFI------------------------LGHVTFQICLYT--------------------

D.melanogaster (AFB77909): -------------------------LVLLGHITLQILAVS--------------------

M.musculus (NP_001032375): LLFLVA------------------------HLAFQICLHT--------------------

R.norvegicus (NP_001070668): LLFLVA------------------------HVAFQICLHT--------------------

B.taurus (XP_002694905): SVILLA---LWAMCPSRAQ----LQQKFQGNLRVSSCDLINRDLRGQALQL---------

C.lupus (XP_546782): LIFLAA------------------------HLTFQICLHT--------------------

A.thaliana (NP_182327): ----------WRNPTVMYF----L------ALQLLTSLVALAD-----------------

O.sativa (NP_001172359): ------------------------------------------------------------

hPiezo1 (NP_001136336): ------------------------------------------------------------

hPiezo2 (NP_071351): ------------------------------------------------------------

E.histolytica (XP_649449): ------------------------------------------------------------

E.histolytica (XP_655549): ------------------------------------------------------------

L.major (XP_001686914): WDAADYVNTIVWPGATAVV----LGAY----LLILKRTL------AGRVLSRNDAMRAVQ

L.infantum (XP_001469682): WDAADYVNTIVWPLATAVV----LGAY----LSILKRTL------AGRVLSRNDATRTVQ

T.cruzi (XP_819187): W-----------ILAGACVPSGLICVY----IIYFRKKL------LSRI-----------

T.cruzi (XP_812333): ------------------------------------------------------------

L.major (XP_001686223): ------------------------------------------------------------

L.infantum (XP_001468509): ------------------------------------------------------------

L.braziliensis (XP_001564414): ------------------------------------------------------------

T.cruzi (XP_817508): WGRKDVLP-----LVVNVVSSTLLAATSAALCFLLRKHQFQPSTEPNRLFHRSSLLCLAL

T.cruzi (XP_820998): WGRKDVLP-----LVVNVVSSTLLAVSSASLCFLLRKHQFQPSTEPNRLFHRSSLLCLAL

T.vaginalis (XP_001319509): ------------------------------------------------------------

T.vaginalis (XP_001582897): ------------------------------------------------------------

T.vaginalis (XP_001581503): ------------------------------------------------------------

T.vaginalis (XP_001580012): ------------------------------------------------------------

T.vaginalis (XP_001305124): ------------------------------------------------------------

N.gruberi (XP_002682879): IDIGEGQNETMYAMQVDDVVTTKVSTS-------------------NTKLDDS-ALKKQQ

D.discoideum (XP_640187): FNSGSNNNN-----------------------------------------------NNNN

D.rerio (XP_696355): ------------------------------------------------------------

D.melanogaster (AFB77909): ------------------------------------------------------------

M.musculus (NP_001032375): ------------------------------------------------------------

R.norvegicus (NP_001070668): ------------------------------------------------------------

B.taurus (XP_002694905): -LSGEALEAF---------LEVAHCSS-------------------DTFLPRP----GTQ

C.lupus (XP_546782): ------------------------------------------------------------

A.thaliana (NP_182327): ------------------------------------------------------------

O.sativa (NP_001172359): ------------------------------------------------------------

hPiezo1 (NP_001136336): ---VPRLDQLLGPSCSRWE--TLS--R------------------------------HIG

hPiezo2 (NP_071351): ---EAQHRIAPGYNCSTWEKT----------------------------------FRQIG

E.histolytica (XP_649449): ------------------------------------------------------------

E.histolytica (XP_655549): ------------------------------------------------------------

L.major (XP_001686914): DRPVPS-SLLPRWAGM-----ALAA--------------------------------HRP

L.infantum (XP_001469682): DRPVPS-SFLPRWAGA-----ALAA--------------------------------HRP

T.cruzi (XP_819187): ---------------------------------------------------------SMG

T.cruzi (XP_812333): ------------------------------------------------------------

L.major (XP_001686223): ------------------------------------------------------------

L.infantum (XP_001468509): ------------------------------------------------------------

L.braziliensis (XP_001564414): ------------------------------------------------------------

T.cruzi (XP_817508): LAPVTA-SLYPTYDC---------------------------------------------

T.cruzi (XP_820998): LAPVTA-SLYPTYDC---------------------------------------------

T.vaginalis (XP_001319509): -------------------------------------------------------TNEIK

T.vaginalis (XP_001582897): -------------------------------------------------------SEEPK

T.vaginalis (XP_001581503): ------------------------------------------------------------

T.vaginalis (XP_001580012): --------AAPR---------------------------KK--VYQEP----VRKKRQKS

T.vaginalis (XP_001305124): --------KLPT---------------------------TK--SYANIPMPRKKKKDHII

N.gruberi (XP_002682879): IESIPKVKGA---TMYMWDLPGVFTKHPKLLTCSVQELKTSVTHVGSMPPKEKTEKKHVN

D.discoideum (XP_640187): IVYRPAGRGL---TGF--------------------------------------------

D.rerio (XP_696355): ---IPSLDDALGHNCSSWETL----------------------------------SRHVG

D.melanogaster (AFB77909): -------LTLPIYNCSFSERL----------------------------------LRHIG

M.musculus (NP_001032375): ---VPHLDQFLGQNGSLWVK--V--------------------------------SQHIG

R.norvegicus (NP_001070668): ---MPRLNQLLGQNCNLWAN--V--------------------------------SQHIG

B.taurus (XP_002694905): MTQVPLSTFLIRTAMEAWGWGDSG--HTVRGDCARGQPALI-----LLPMGHLESEAALG

C.lupus (XP_546782): ---VPHLYQLLEPSCGPWE--TLS--R------------------------------HIG

A.thaliana (NP_182327): ------------------------------------------------------------

O.sativa (NP_001172359): ------------------------------------------------------------

hPiezo1 (NP_001136336): VT---RLDL----------------KDIPNAIRLVAPDLGILV-----------------

hPiezo2 (NP_071351): FE---SLKG----------------ADAGNGIRVFVPDIGMFIASLT-------------

E.histolytica (XP_649449): ------------------------------------------------------------

E.histolytica (XP_655549): ------------------------------------------------------------

L.major (XP_001686914): AA-KPLLSLFS--WAAAWTAEPS----------LVGLCLEIFTFVFVAAFLYVEHRIRKP

L.infantum (XP_001469682): AA-GPLLSLFS--WAAAWTAEPS----------LVGLCLEGFTFVFAAAFLYVEHRIRKP

T.cruzi (XP_819187): RV-RPLLTTVS--WALMSVATGL----------IFPTVLAGVLLVIGMMAL--S-CVGL-

T.cruzi (XP_812333): ------------------------------------------------------------

L.major (XP_001686223): ------------------------------------------------------------

L.infantum (XP_001468509): ------------------------------------------------------------

L.braziliensis (XP_001564414): ------------------------------------------------------------

T.cruzi (XP_817508): --------------------TPL----------FVVHFLCMMSFS-----------ISSF

T.cruzi (XP_820998): --------------------TPL----------FVVHFLCIMSFS-----------ISSF

T.vaginalis (XP_001319509): FSVGKLLCLLEAVLPILWIYAACEASYIGTLLH----MVFIFFYSIGMNSV---------

T.vaginalis (XP_001582897): FTVGKLSCLTEVFLPCLWLYAACEGSYGNTLAH----LVFILLYAIGVNSV---------

T.vaginalis (XP_001581503): ------------------------------------------------------------

T.vaginalis (XP_001580012): YP-IPCIVLLEFILPICYVINSMTTEGVMGMIN----III-LVANVAITNL---------

T.vaginalis (XP_001305124): FP-IMTIVLLELILPICYLINSISTQSIMGLLN----III-LIVNVIITNL---------

N.gruberi (XP_002682879): IH---ELKLII--FALFLMITASFYPSFVGIPYLLLSFLIITLASFG-SLK-TFISIGLF

D.discoideum (XP_640187): -------S------FLMLTLSSISYPSIINVIYFVFTILIILLLASKLSIH-KV-MLKCY

D.rerio (XP_696355): VS---RLPL----------------EDPQGVLWLLTPDLGVFIMSLI----------T--

D.melanogaster (AFB77909): FV---SFID----------------LQPFAIIEWLVPEVLVFATSLG----------S--

M.musculus (NP_001032375): VT---RLDL----------------KDIFNTTRLVAPDLGVLL-----------------

R.norvegicus (NP_001070668): VT---RLDL----------------KDIFNTTRLVAPDLGVLV-----------------

B.taurus (XP_002694905): VQ---A-------------------ESVGEPGALVTPQLHISQLGSL----------GKP

C.lupus (XP_546782): VT---RLDL----------------KDIPTAVRLVAPDLGVLV-----------------

A.thaliana (NP_182327): ------------------------------------------------------------

O.sativa (NP_001172359): ------------------------------------------------------------

hPiezo1 (NP_001136336): ----VS---------------------------SVCLG----------------------

hPiezo2 (NP_071351): ----IW---------------------------LLCRNI--VQKP-----VTDEAAQSNP

E.histolytica (XP_649449): ------------------------------------------------------------

E.histolytica (XP_655549): ------------------------------------------------------------

L.major (XP_001686914): PRRAVQSGKGAQRRIPHAF-----------G-TAVAVVL-VGVCVTQNSAF---------

L.infantum (XP_001469682): PGRAVQSGKGAQRHIPHAF-----------G-TAVAVVL-VCVCVTQNGAF---------

T.cruzi (XP_819187): --GGARTIFGAARSRPRRFLLLFLHFCSLCGTFASCIIL-VASCVSQNAAV---------

T.cruzi (XP_812333): ------------------------------------------------------------

L.major (XP_001686223): ------------------------------------------------------------

L.infantum (XP_001468509): ------------------------------------------------------------

L.braziliensis (XP_001564414): ------------------------------------------------------------

T.cruzi (XP_817508): PVLRVRLLEVAT-----------------KGCTVVSLLLLAGGLLAQSPVVQKIVLEHGE

T.cruzi (XP_820998): PVLRVRLLEVAT-----------------KGCTVVSLLLLAGGLLAQSPVVQQIVLEHGE

T.vaginalis (XP_001319509): ------------------------RIMTNSLYKWPFLFV---------------------

T.vaginalis (XP_001582897): ------------------------HKMSTKFHRLPCSII---------------------

T.vaginalis (XP_001581503): ------------------------------------------------------------

T.vaginalis (XP_001580012): ------------------------TKKSITGHKIIL------------------------

T.vaginalis (XP_001305124): ------------------------TKKSITGHKVIL------------------------

N.gruberi (XP_002682879): IFTQVY---------------------------SILLLI--FYFLT---RFQTNSISSTG

D.discoideum (XP_640187): PILLIT---------------------------SLCHLL--FVYLN---QIEYFYMKYT-

D.rerio (XP_696355): -----L---------------------------ILC------------------------

D.melanogaster (AFB77909): -----Y---------------------------LTVKRV--ASQP-----VGA-------

M.musculus (NP_001032375): ----AS---------------------------SLCLGL---------------------

R.norvegicus (NP_001070668): ----AS---------------------------SLCLGL---------------------

B.taurus (XP_002694905): PNLSES---------------------------VFCLSN--PGPS------SGKAAVENE

C.lupus (XP_546782): ----VC---------------------------AVCLG----------------------

A.thaliana (NP_182327): ------------------------------------------------------------

O.sativa (NP_001172359): ------------------------------------------------------------

hPiezo1 (NP_001136336): ------------------------------------------------------------

hPiezo2 (NP_071351): EF----------------------------------------------------------

E.histolytica (XP_649449): ------------------------------------------------------------

E.histolytica (XP_655549): ------------------------------------------------------------

L.major (XP_001686914): -FFFTNARG---------DWCRLLG--MGPA-------ALD--------T---VAGLRYA

L.infantum (XP_001469682): -FFFTNAHG---------DWCRLLG--MGPA-------ALD--------T---VAGLRYA

T.cruzi (XP_819187): -ACALDNLD---------PMYGLNKLYEQPA-------DI--------------AICRFV

T.cruzi (XP_812333): ------------------------------------------------------------

L.major (XP_001686223): ------------------------------------------------------------

L.infantum (XP_001468509): ------------------------------------------------------------

L.braziliensis (XP_001564414): ------------------------------------------------------------

T.cruzi (XP_817508): PIYVWSIMR---------YECDWAKIFKSAA-------VFR-------------RGAQFL

T.cruzi (XP_820998): PIDAWSIMR---------YECDWAKIFKSSA-------VFR-------------RGAQFL

T.vaginalis (XP_001319509): -----------------------------------------------------LLSQVYS

T.vaginalis (XP_001582897): -----------------------------------------------------LFSLIYS

T.vaginalis (XP_001581503): ------------------------------------------------------------

T.vaginalis (XP_001580012): -----------------------------------------------------SIELVYN

T.vaginalis (XP_001305124): -----------------------------------------------------SIELVYN

N.gruberi (XP_002682879): YFSKMILQN-------------PYGKMAGFF-DIDGNLKSFEFFK---KFYTGYVFITFV

D.discoideum (XP_640187): ---VFKEQK-------------WYGV-----------LNYTEWDVTYWPLVIGYIT---V

D.rerio (XP_696355): ------------------------------------------------------------

D.melanogaster (AFB77909): ------------------------------------------------------------

M.musculus (NP_001032375): ------------------------------------------------------------

R.norvegicus (NP_001070668): ------------------------------------------------------------

B.taurus (XP_002694905): RFWVWEEQKQLLGERTDPPQEQLLGQMLPAAPARSGTVRCDASA----PR---DTHLTSP

C.lupus (XP_546782): ------------------------------------------------------------

A.thaliana (NP_182327): --------------------------------IYSSRFGFARWRDTWWSHFSGIF-----

O.sativa (NP_001172359): ------------------------------------------------------------

hPiezo1 (NP_001136336): ----------------ICG-----------------------RLARNT-RQSPHPRELD-

hPiezo2 (NP_071351): ---------------------ENE----------ELAEGE---------KIDSEEALIY-

E.histolytica (XP_649449): ------------------------------------------------------------

E.histolytica (XP_655549): ------------------------------------------------------------

L.major (XP_001686914): MQMVGMAGVFL-GAAFMHHG-------------TESSESTLPDSG---------------

L.infantum (XP_001469682): MQMVGMAGVFL-GAAFMCHG-------------TESSESALPDSG---------------

T.cruzi (XP_819187): QLCLMMVTLFLAQMAMECHG-------------KELQQQNAGDPL---------------

T.cruzi (XP_812333): ------------------------------------------------------------

L.major (XP_001686223): ------------------------------------------------------------

L.infantum (XP_001468509): ------------------------------------------------------------

L.braziliensis (XP_001564414): ------------------------------------------------------------

T.cruzi (XP_817508): LLAYTIVASRYVHGSWRCIS-------------RRE------------------------

T.cruzi (XP_820998): LLSYTIVASRYVHGSWRRIS-------------RRE------------------------

T.vaginalis (XP_001319509): ALYVIAELS---------IYYMPQ-------------------------NKKVKITPILK

T.vaginalis (XP_001582897): LAYFITETT---------IYYLPK-------------------------NKRPTITKAMK

T.vaginalis (XP_001581503): ------------------------------------------------------------

T.vaginalis (XP_001580012): IILLGLASS---------IGMREE-------------------------EDTN-IIKIM-

T.vaginalis (XP_001305124): IILLACAAT---------LGYRSD-------------------------RLTNKALIYL-

N.gruberi (XP_002682879): LITFIL----------ACNFVC-QY------IAKVLQERELQRQV----KKFLTLLPTM-

D.discoideum (XP_640187): LLLYIS----------TCILFR-K---------QQLFNRTKPR-Y----KQKLDKLGIM-

D.rerio (XP_696355): ------------------SRLLKKRDEGSVPHMSAL-------------LHEADETEDD-

D.melanogaster (AFB77909): ---------------------------------EQLENGEVVDGQAEN-AQTSSQ-----

M.musculus (NP_001032375): -----------------CGRLTRK-------------------ARQSRRTQELDDDDDD-

R.norvegicus (NP_001070668): -----------------CGRLTRK-------------------ARQSQRTQELEED----

B.taurus (XP_002694905): LLSRLSTPSLYA-SKGSGGRFWLQKLVQAGHSMQAYLEGDVGCQEEEAEAQQGSEEATS-

C.lupus (XP_546782): ----------------LCG-----------------------RLTQEA-PRSQRAQELD-

A.thaliana (NP_182327): ------------------------------------------------------------

O.sativa (NP_001172359): ------------------------------------------------------------

hPiezo1 (NP_001136336): -DD-ERDVDASP--TAGLQE---------A-------------------ATLAP------

hPiezo2 (NP_071351): ----EEDFNGGDGVEGELEE---------S-------------------TKLKM-F----

E.histolytica (XP_649449): ------------------------------------------------------------

E.histolytica (XP_655549): -----------------MKT-----------------------------LNTKNVFQKGK

L.major (XP_001686914): --ADAAV-------P--ASG-----EATPLL-AAMDQRVLATPAPDASLPSTS-------

L.infantum (XP_001469682): --ADAAV-------P--ASG-----EATRLL-AARDQPVLVTPVPDAFSPSTS-------

T.cruzi (XP_819187): --LREEVSNNNARVVEGTQG-----TSVAVVFNHADVPSA-PNFASAAVPSHSNHHLSRP

T.cruzi (XP_812333): ------------------------------------------------------------

L.major (XP_001686223): ------------------------------------------------------------

L.infantum (XP_001468509): ------------------------------------------------------------

L.braziliensis (XP_001564414): ------------------------------------------------------------

T.cruzi (XP_817508): ------L------------------------------------------EEHREYFYEPP

T.cruzi (XP_820998): ------L------------------------------------------EEHREYFYEPP

T.vaginalis (XP_001319509): LLDLFNKEDKNLFKTPYF-VLQIVLIIINFIYL----------------TS---------

T.vaginalis (XP_001582897): CFDFFDNVDKIMFKTTFV-ILQMIIIFVDFIYL----------------AS---------

T.vaginalis (XP_001581503): ------------------------------------------------------------

T.vaginalis (XP_001580012): GLDFNNYVASNAKLTIGISVIAIILEILSAVLL----------------FKTKP-----E

T.vaginalis (XP_001305124): GLDFNNTVVKSPLLTIVVSSIAIFLELVAVVFI----------------SKTKP-----E

N.gruberi (XP_002682879): -ISNKEDLDTNS--EEYLQD---------LYYKSEER----------RLIEIDEHTSMGG

D.discoideum (XP_640187): -SDSNNNNNNNN-----------------------KS----------RTTKLAIIFSK--

D.rerio (XP_696355): -DEDVEGEDEGMLSCSETED---------E-------------------ESPSS------

D.melanogaster (AFB77909): --PSAADANGGD-----VQQ---------A-------------------TVTTP-LQQQQ

M.musculus (NP_001032375): -DDDDEDIDAAP--AVGLKG---------A-------------------PALAT------

R.norvegicus (NP_001070668): ------DIDAAP--AAGLQG---------A-------------------PTLAT------

B.taurus (XP_002694905): -DDDSEELDTGS--LGELRE---------A-------------------PELSP------

C.lupus (XP_546782): -DDDDGEVDTGS--PVGLQG---------A-------------------PVQAP------

A.thaliana (NP_182327): ------------------------------------------------------------

O.sativa (NP_001172359): ------------------------------------------------------------

hPiezo1 (NP_001136336): --------TRRSRLAARFRVTAHWLLVA-----------------------------AG-

hPiezo2 (NP_071351): --------RRLASVASKLKEFIGNMITT-----------------------------AG-

E.histolytica (XP_649449): -----------------MSVLRVLSYLV-T---------------------------CY-

E.histolytica (XP_655549): FLV-----VSFSGIFTETYILRGWSILI-T---------------------------LY-

L.major (XP_001686914): --------ARGEGLPNRHRRTPQ----VVAIVCI-------------------TALLAYA

L.infantum (XP_001469682): --------ARGERLPNRHRRAPQ----VVAIACI-------------------TALLAYA

T.cruzi (XP_819187): LNVPHGLSLAGHSSPNSSAAFGTNYLERGLVVCGSLGTWMMRKLFASSRMLPLAFMAANM

T.cruzi (XP_812333): ---------------------------------------MMRKLFASSRMLPLAFMAANM

L.major (XP_001686223): -----------------------------------------MPELP--YAVV------NG

L.infantum (XP_001468509): -----------------------------------------MSELL--YAVV------NG

L.braziliensis (XP_001564414): ---------------------------------------MAMSELP--YAIA------NS

T.cruzi (XP_817508): FLLWQ---LFSVRYPARLRKIQQ-----------FMGYYLPLAVFP--LMIIMSFMVMDL

T.cruzi (XP_820998): FLLWQ---LFSVRYPARLRKIQQ-----------FMGYYLPLAVFP--LMIIMSFMVMDL

T.vaginalis (XP_001319509): ----------RS--TFI----LLWYKQSRFVIIK-HLMVLIRPIFL----FTLAVLVTS-

T.vaginalis (XP_001582897): ----------RT--TFG----LAWYSQARIVIMR-HVMILIRPLFL----FTQSVLVAS-

T.vaginalis (XP_001581503): ------------------------------------------------------------

T.vaginalis (XP_001580012): IML-----ERRAKIYSS----MAWIFASDFI-------------WT----FCLAVMGAV-

T.vaginalis (XP_001305124): QMI-----RLREMFFAS----MGWIFSADFI-------------WC----FCLAYAGAS-

N.gruberi (XP_002682879): -IA-----GGVHSIYESSYKFRMFLYEQLQ---------------------------KF-

D.discoideum (XP_640187): ---------------------HGWTIC---------------------------------

D.rerio (XP_696355): --------SSAAQLAARLRATAQRFLRN-----------------------------MG-

D.melanogaster (AFB77909): QQL-----RKRVSMISQHIHFE--GLVK-----------------------------IS-

M.musculus (NP_001032375): --------KRRLWLASRFRVTAHWLLMT-----------------------------SG-

R.norvegicus (NP_001070668): --------KRRLWLAPRFRITAHWLLVT-----------------------------SG-

B.taurus (XP_002694905): --------TRRSRLAARFRITAHWLLVA-----------------------------AG-

C.lupus (XP_546782): --------TRRSRLATRFQITAHWLLVA-----------------------------AG-

A.thaliana (NP_182327): ---------------EHL----GSHLRV-----------------------------AS-

O.sativa (NP_001172359): ------------------------------------------------------------

hPiezo1 (NP_001136336): RVLAVTLLALAGIAHPSALSSVYLLLFLALCTW-WAC------HFPISTRGFSRL---CV

hPiezo2 (NP_071351): KVVVTILLGSSGMMLPSLTSSVYFFVFLGLCTW-WSW------CRTFDPLLFSCL---CV

E.histolytica (XP_649449): SIFHFTLNIIFV------FCGSDNSFVL-----------------FLCGLNFKYY---GI

E.histolytica (XP_655549): SFVHFIILLIFY------FVDIEEHWGL-----------------MLCGLMFKNQ---KQ

L.major (XP_001686914): VY------------YPSLVSTAVWLLHLCCC-----------AEGLVQNPMAPPS-----

L.infantum (XP_001469682): VY------------YPSLVSTAVWLLHLCCC-----------AGGLVQSPMAPPS-----

T.cruzi (XP_819187): VA------------FPSVVSLSLLPTM---M-----------LGQLVRFHFFA-S-----

T.cruzi (XP_812333): VA------------FPSVVSLSLLPTM---M-----------LGQLVRFHFFA-S-----

L.major (XP_001686223): AYLFLVLQLL----------------------------------------FLLAV-----

L.infantum (XP_001468509): AYLFLVLQLL----------------------------------------FLLVV-----

L.braziliensis (XP_001564414): AYLFLVLHLL----------------------------------------FVLTV-----

T.cruzi (XP_817508): VSILMVITTLFGFLLPSDTYVALLPFCYGAIALGIVLEFVATVDSRLQQPFFMVYAATGV

T.cruzi (XP_820998): VSILMVITTLFGFLLPSDTYVVLLPFCYGAIALGIVLEFVATVDSRLQQPFFMVYAATGV

T.vaginalis (XP_001319509): -C-----NSLKLSFMPVIFALTIISFSLLSLNL----------------ANW--------

T.vaginalis (XP_001582897): -T-----NSIYLTFMPVIFALKIISFCLLSCNM----------------PKF--------

T.vaginalis (XP_001581503): ------------------------------------------------------------

T.vaginalis (XP_001580012): -QMFMEIVEVSYCFLPLLVYILYISITMGIFGR----------------PFV--------

T.vaginalis (XP_001305124): --------NTSYLFMPLLLYFVYISITMGIIGR----------------PCV--------

N.gruberi (XP_002682879): ILYALMIALICIILSVPSLCSLPYLLFLYLGMV------------LSPGPRFETN---KV

D.discoideum (XP_640187): ---CSQILVVCFFLTASVA----SAILLASGLI----------CTLLPLKVFK-----KV

D.rerio (XP_696355): RILAVTLLALAGITLPSAFSAFYFLLFIGVCTW-WAC------HFPISHLGFNAL---CV

D.melanogaster (AFB77909): PLFCLATLFFAAVLRPSVPGGFYFLIFLLSGTY-WAT------CQTLQ-RGFALL---LR

M.musculus (NP_001032375): RTLVIVLLALAGIAHPSAFSSVYLVVFLAICTW-WSC------HFPLSPLGFNTL---CV

R.norvegicus (NP_001070668): RMLVIVLLALAGIAHPSAFSSVYLMVFLAICTW-WSC------HFPLSSLGFNTL---CV

B.taurus (XP_002694905): RTLAIMLLALAGIAHPSAFSSVYFLLFLAIGTW-WAC------HFPISLLGFNTL---CV

C.lupus (XP_546782): RTLAIVLLALAGIAHPSAFSSVYFLVFLATCTW-WAC------HFPISLLGFSTL---CV

A.thaliana (NP_182327): CLLLPAVQLAVGICN-PSWVSLPFFIGSCAGLVDWSL------TSNVSG-LF--------

O.sativa (NP_001172359): ------------------------------------------------------------

hPiezo1 (NP_001136336): --AVGCFG-----------AGH---------LICLY--------CYQMPLAQALLPP-AG

hPiezo2 (NP_071351): --LLAIFT-----------AGH---------LIGLY--------LYQFQFFQEAVPP-ND

E.histolytica (XP_649449): --VRSCLHFVSVLVLFFFSFLY---------CLTKK--------VSWQPIPSKFAPS-LT

E.histolytica (XP_655549): --EFYILHTIFVGVIGIISFFG---------IFSRS--------EKIIIRHTALVPT-LT

L.major (XP_001686914): ------AHRIAIACVLAVLCCT---------ILVQY--------VFQALAGNPRTAAWGQ

L.infantum (XP_001469682): ------PHRIAIACALAVLCCT---------ILVQY--------VFQALAGNPRTAAWGQ

T.cruzi (XP_819187): ------L----LRPTLVLNFFL---------VNVQY--------VVLATASIQPPIV---

T.cruzi (XP_812333): ------L----LRPTLVLNFFL---------VNVQY--------VVLATASIQPPIV---

L.major (XP_001686223): ------VWQSVVAASLLFSFVS---------L----------------SCLESVR--WG-

L.infantum (XP_001468509): ------VWQNVVAASLLFLFVS---------L----------------SCLEAVR--WG-

L.braziliensis (XP_001564414): ------VWQSIVAAALLVAFLS---------F----------------SCLESVT--SG-

T.cruzi (XP_817508): TAFMIRLAQCFI-NSLLIL------------A----------------VCMFRVNPIQGQ

T.cruzi (XP_820998): TAFMIRLAQCFV-NSLLIL------------A----------------VCMFRVNPIQGQ

T.vaginalis (XP_001319509): -------I------TWIYWIIY---------ILADFAFIAYKDQTFKEPVGYNWVPRWAV

T.vaginalis (XP_001582897): -------V------SHIYWTLY---------VIGDIGFIAYKDQSFHGTATYAWVPRWAV

T.vaginalis (XP_001581503): ------------------------------------------------------------

T.vaginalis (XP_001580012): -------HQYILIVIYVYAIIFSLFIFYTTSPLGQLYPIIGKTS-SAKIHSSSYFQKYAP

T.vaginalis (XP_001305124): -------PHIMQYVVYGYSILYSMYLFYQVSNIGKIYSIANQIK-FVMLA----NRKYDY

N.gruberi (XP_002682879): --MVILLPF-----CFIYSLLY---------ICAQY--------VFQMPNFASYIPQ-TT

D.discoideum (XP_640187): --IYI---------ILLYFLVF---------ISAQY--------IFNIPFSYSE-----T

D.rerio (XP_696355): --MVAFFT-----------GGH---------LVCLY--------LYQSSFAQAMFSP-AG

D.melanogaster (AFB77909): --CVMVVL-----------VLH---------SLSIV--------SYQTPWMQSHLNH-TT

M.musculus (NP_001032375): --MVSCFG-----------AGH---------LICLY--------CYQTPFIQDMLPP-GN

R.norvegicus (NP_001070668): --MVSCFG-----------AGH---------LVCLY--------CYQTPFVQSVLLP-GS

B.taurus (XP_002694905): --MVSCIG-----------TGH---------LICLY--------CYQTPLAQATLPP-AG

C.lupus (XP_546782): --TVGCFG-----------AGH---------LLCIY--------CYQTPFAQTVLPP-AG

A.thaliana (NP_182327): ---------RWWRVLYIYAGFN---------IVLLY--------LYQLPINFSDMIRWIA

O.sativa (NP_001172359): ------------------------------------------------------------

hPiezo1 (NP_001136336): IWARVLGLKDFVGPTNCSSPHALVLNTGLDW-----------------------------

hPiezo2 (NP_071351): YYARLFGIKSVIQ-TDCSSTWKIIVNPDLSW-----------------------------

E.histolytica (XP_649449): SLV--LFCSSLVL-N-TSQGGIIPLVL---M--------------------------V--

E.histolytica (XP_655549): ALF--FFISAFVV-N-SSQGGIIPILF---F--------------------------L--

L.major (XP_001686914): WSP-------ARPAY--PDDG--------CAVG-AQ------------------------

L.infantum (XP_001469682): WSP-------ARPAY--PDDG--------RAVG-AQ------------------------

T.cruzi (XP_819187): ----------RHLFL--GDEG--------RWWG-LAQ-----------------------

T.cruzi (XP_812333): ----------RHLFL--ADEG--------RWWG-LAQ-----------------------

L.major (XP_001686223): ----------ALDSG--KQQN--------QSLH---------------------------

L.infantum (XP_001468509): ----------ALESG--KQQN--------QSLN---------------------------

L.braziliensis (XP_001564414): ----------VPETR--QQPR--------GSPN---------------------------

T.cruzi (XP_817508): YRS-------LAEKK--AQIR--------MAFNRLQNRIDEIQCIFKRVCNS--------

T.cruzi (XP_820998): YRS-------LAEKK--AQIR--------MAFNHLQNRLDEIQCIFKRVCNG--------

T.vaginalis (XP_001319509): VSS--------------AATNLLFL---------------GMIIGVNRNTTVHTIRLF--

T.vaginalis (XP_001582897): VSA--------------AGVNLLLI---------------GAIIGCNRYTHSFPIRLL--

T.vaginalis (XP_001581503): ------------------------------------------------------------

T.vaginalis (XP_001580012): LSL---------------AVNVIQI-----WFS-V-----QLCFVFKRS-----------

T.vaginalis (XP_001305124): LVL---------------SVNIIQI-----WLS-T-----QLCFAFNRA--------G--

N.gruberi (XP_002682879): EM------SANEF-NPSGSGGVLFYPLQ-GILY-RAI--------------------V--

D.discoideum (XP_640187): DL---------------QSYGLFSFNNS-KWLY-I-------------------------

D.rerio (XP_696355): LWARLFGLKDLVTPGNCTTFE-VTLNTQYDW-----------------------------

D.melanogaster (AFB77909): LTARLIGLEPLIESYCSPDIRVFLYNNKLSL-----------------------------

M.musculus (NP_001032375): IWARLFGLKNFVDLPNYSSPNALVLNTKHAW-----------------------------

R.norvegicus (NP_001070668): LWARLFGLKNFVDIPNCSSPNVLVLNTKHAW-----------------------------

B.taurus (XP_002694905): IWARVFGLKDFLAPTNCSSPNVLVINANHDW-----------------------------

C.lupus (XP_546782): IWARVFGLKALVTHGNCSSPNVLVFSTSHDW-----------------------------

A.thaliana (NP_182327): SFIGLFRISLETE-GPDICSGLFLVLFYIMLSY-VRSDLEDMDFIMSTSENNLAERLLPP

O.sativa (NP_001172359): ------------------------------------------------------------

hPiezo1 (NP_001136336): ----------------------PVYASPGVL---------LLLCYAT-------------

hPiezo2 (NP_071351): ----------------------YHHANPILL---------LVMYYTL-------------

E.histolytica (XP_649449): -------ISTISFFVPKRIQTVIF-VLTTILLSIYLLCWTLFTIYAWTA-----------

E.histolytica (XP_655549): -------ASIVLIISPRIIFKFLS-VSLAFIMFIYLIILSLWNVFSFVS-----------

L.major (XP_001686914): -----------------VVA-------EHLI------ALL-FGVYVSIAGV---------

L.infantum (XP_001469682): -----------------VVA-------EHLI------ALL-FGVYVSIAGV---------

T.cruzi (XP_819187): -----------------VIS-------SEII------FLAMLAADAAIRQQ---------

T.cruzi (XP_812333): -----------------VVS-------SEII------FLAMLAADAAIRQQ---------

L.major (XP_001686223): ---------------------------AEVV------VVRPSTAEVSRKQN---------

L.infantum (XP_001468509): ---------------------------AEVV------VVSPSTAELSRREN---------

L.braziliensis (XP_001564414): ---------------------------AEHV------VVGSSTVDVKEKED---------

T.cruzi (XP_817508): ---------TTG----TVNREGF----PELL------KLVVPTSRIREKEI---------

T.cruzi (XP_820998): ---------TTG----TVNRDGF----PELL------KQVLPTSRIREKEI---------

T.vaginalis (XP_001319509): ------KNATAPTWFMD--------IIPYVVLISCGVF--CICRDAWESFGVLALACFTT

T.vaginalis (XP_001582897): ------KNVKVPGWFLH--------IIPYIVIVTSGLF--CILEDSWQSYGVLAFASFTS

T.vaginalis (XP_001581503): ------------------------------------------------------------

T.vaginalis (XP_001580012): -------KPTIPQSLVTLSD--ILLVFSFVAVLVYSVFFSCYIAGVWELIV--YLACFFS

T.vaginalis (XP_001305124): ------KAPKIPAFLTSASD--FVLVFSFVGVLVYAVFYPCYISVLWIIIV--YITAFFN

N.gruberi (XP_002682879): -------GTTVGAFLTDVSDSGRSIVLVAVI-FG-QLILSLFMGLTV-------------

D.discoideum (XP_640187): ---------------------GVQIV--------VSLTLSLYCFYSDIK-----------

D.rerio (XP_696355): ----------------------PVYVNPGIL---------LLLYISV-------------

D.melanogaster (AFB77909): ----------------------DSYLNPFAL---------FFAYFAL-------------

M.musculus (NP_001032375): ----------------------PIYVSPGIL---------LLLYYTA-------------

R.norvegicus (NP_001070668): ----------------------PIYVSPGIL---------LLLYYTA-------------

B.taurus (XP_002694905): ----------------------PVYVSPGIL---------LLLCYTV-------------

C.lupus (XP_546782): ----------------------PIYVSPGIL---------LLLYYTV-------------

A.thaliana (NP_182327): KYSFFIRESRAGVRHTNVLLRGAVFKTFSINFFTYGFPVSLFALSFWSFHF---------

O.sativa (NP_001172359): ------------------------------------------------------------

hPiezo1 (NP_001136336): -----------------------------ASLRKLRAYRPSGQRKEAAKG----------

hPiezo2 (NP_071351): -----------------------------ATLIRIWLQEPLVQDEGTKEEDKAL----AC

E.histolytica (XP_649449): ---------------HEYEFIND------LVLKNRMKD--------------IF----ET

E.histolytica (XP_655549): ---------------NEYNFFQV------TEL-DVLQK--------------AM----ST

L.major (XP_001686914): -SD-T--------GGGG-----N------SA-------REGAEPAAPPRSSLVSRV----

L.infantum (XP_001469682): -DG-T--------GGGG-----N------SA-------HEGAEPAAPSRSSLVNRV----

T.cruzi (XP_819187): -YRLA--------HSEE-----D------HP-------DEIVVPHPPPLPSIAPSS----

T.cruzi (XP_812333): -HRLT--------HSEE-----D------HP-------DEIVVPHPPPLPSIAPSA----

L.major (XP_001686223): -DGGTEVSVSALHATHK-----P------PAPDGCRASCYRCTP-PPPREERSSVTIASC

L.infantum (XP_001468509): -DGGTEVFVSALYATHE-----P------PALDGCRASCYRCTP-PPPREERSSVAIASW

L.braziliensis (XP_001564414): -GGGTEVFVSAVYASRE-----S------P---------------SLPHEECSSVVITSW

T.cruzi (XP_817508): -DSLWR------FLTEG-----E------PVL-------LQTTPQVTPRINKATLE---F

T.cruzi (XP_820998): -DSLWR------FLTEG-----E------PVL-------LQTTPQVTPRINKATLE---F

T.vaginalis (XP_001319509): LFGLK-FLQK----ASRFVLTALAIVYCYQIFDDLARNKTEHHPK---------------

T.vaginalis (XP_001582897): LFGVD-FLQR----SSRFVLTAMAGVYCGQTLYFYIKKQPGFHPK---------------

T.vaginalis (XP_001581503): ------------------------------------------------------------

T.vaginalis (XP_001580012): LNASRKFFFRLLSVIHNILFS-AIYVTCLNLFYS-AEKAKTNHPRLFSRIL---------

T.vaginalis (XP_001305124): LNTAKKVFFRVISLIHNFSFS-CIYVTLADLYPE-SKIAPKTLI-----RL---------

N.gruberi (XP_002682879): ------------------RVSAV------SKLLKRHKK--------------TV----ST

D.discoideum (XP_640187): ---------------DD----DL------GTIKKDQQS-QQSQPQPQQQQQQQQ----SS

D.rerio (XP_696355): -----------------------------TIVLKINSNSLGDADKAREGEAV--------

D.melanogaster (AFB77909): -----------------------------ALTTKHLI-KPRLVRQSTRKAR---------

M.musculus (NP_001032375): -----------------------------TSLLKLHKSCPSELRKETPRE----------

R.norvegicus (NP_001070668): -----------------------------TSLLKLRKGRFSELRKEIPRE----------

B.taurus (XP_002694905): -----------------------------TSLLKLHAHQPVDQRKEAAGD----------

C.lupus (XP_546782): -----------------------------TSLLKLRTRQPLDQRKEVARN----------

A.thaliana (NP_182327): -ASLCAF---GLLAYVGYIIYAF------PSLFQLHRL----------------------

O.sativa (NP_001172359): ------------------------------------------------------------

hPiezo1 (NP_001136336): -----YEARELEL---AELDQWPQERESDQ---------HVVPTA-----------PDTE

hPiezo2 (NP_071351): SPIQITAGRRRSL---WYATHYPTDERKLL---------SMTQDDYKPSDGLLVTVNGNP

E.histolytica (XP_649449): IGFTIT---------------------TLN------------------SWVFSW-LMFIS

E.histolytica (XP_655549): VGFSIV---------------------KYP------------------SWGFFG-MNYAA

L.major (XP_001686914): ----------------------GFS---TEANAH-------FSDSVQVTWLRAQ------

L.infantum (XP_001469682): ----------------------GFS---TEANAH-------FTDSVQVTWLRAQ------

T.cruzi (XP_819187): -PTASHVTREF----VALSDGKDSS---SESGGD-------TSLDFLGIWRHAEFKL--G

T.cruzi (XP_812333): -PTASHVAREF----VALSDEKDSS---GESGGD-------TSLDFLGIWRHAEFKL--G

L.major (XP_001686223): MKCDGDLASP-------DMG--PPSTSRERE------PCRDAGNVSAGLYSPIP--N--G

L.infantum (XP_001468509): TGCDGDLASP-------GMG--PPSTSRERE------PCRDAGNVSSGPYSPIQ--N--G

L.braziliensis (XP_001564414): MKRNDDLLSS-------VVD--PSSNSREGE------PCSDAGNTSAELYSPLR--N--G

T.cruzi (XP_817508): AGHADAVRNPLSHLSSALHGGRRASTSREESNGNRDDNDEKGEDMDLEAWEEIK--N--G

T.cruzi (XP_820998): AGNADAVRNPLSHLSSALHGGRRASTSREESNGNRDDNDEKGEDMDLEAWKEIK--N--G

T.vaginalis (XP_001319509): ---------------------------------------------------------Y-N

T.vaginalis (XP_001582897): ---------------------------------------------------------Y-N

T.vaginalis (XP_001581503): ------------------------------------------------------------

T.vaginalis (XP_001580012): ----------------QPIGFYHFYEENTD-----------SEN-----------KNK-E

T.vaginalis (XP_001305124): ----------------NEVGLYHFK--------------------------------D-N

N.gruberi (XP_002682879): SNTAAASTGSQRK---AFLK-------STK------------------YWFHKR-SINNN

D.discoideum (XP_640187): QNNQIQQSPLQYQ---QPLPPTPISNKSLP----------SSPMSTKSTTVHIQ-NNNNG

D.rerio (XP_696355): KTVQGEAGEEVE------LRLWEARRQSSE---------DDTKQTL---------LT--T

D.melanogaster (AFB77909): ---------------------TPQPLESGS---------SVAPSVT---------QRGND

M.musculus (NP_001032375): -----DEEHELEL---DHLEPEPQARDATQ---------GEMPMTT---------EPD--

R.norvegicus (NP_001070668): -----DEEHELEL---DQLEPEPQARGTTQ---------GATPTTT---------GPD--

B.taurus (XP_002694905): -----DDTREVEL---TEVDQWPQGQARHT---------MPTPTT-----------PD-P

C.lupus (XP_546782): -----DEEQEVEL---TQVDQWLQDQARAA---------HMLPTTV---------GSDWE

A.thaliana (NP_182327): --------NGLLL---VFILLWAVSTYIFN-----------------VAFSFLN-TKVGK

O.sativa (NP_001172359): ------------------------------------------------------------

hPiezo1 (NP_001136336): ADNCIVHELTGQSSVL----RRPV----------------------R-------------

hPiezo2 (NP_071351): VDYHTIH---PSLPME----NGPG----------------------KADLYSTPQYRWEP

E.histolytica (XP_649449): YI---F---VCLSC-----------GGIYLLIVF--NKA-----NKK-------QNGYKY

E.histolytica (XP_655549): YL---M---FPLIC-----------LCIKI--IF--NEK-----EPENNSENAPQTGVTE

L.major (XP_001686914): ----------------LSDAAG-----------------------------DQKQMRYLF

L.infantum (XP_001469682): ----------------LSDAAA-----------------------------DRKQMRYLF

T.cruzi (XP_819187): A-----RRVAELLAYVPSEPLE-----------------------------SSEAFEQLF

T.cruzi (XP_812333): A-----RRVAELLAYVPSEPLE-----------------------------SSEAFEQLF

L.major (XP_001686223): A-----DNT---LIYFSSASNGPN-------------------------TER--LIA---

L.infantum (XP_001468509): A-----DDT---LIYFPTASDGPN-------------------------TER--LIA---

L.braziliensis (XP_001564414): A-----DDT---IAYVGSALDMPN-------------------------TER--LIA---

T.cruzi (XP_817508): L-----DST---SCMGALETKSPS-------------------------REGEKSLEAAF

T.cruzi (XP_820998): L-----DSS---SCMGALEIKSPS-------------------------REEEKSLEAAS

T.vaginalis (XP_001319509): L----------VICTL----QA-----ISAVFVH-----------------------CN-

T.vaginalis (XP_001582897): F----------IICVL----QA-----ISAVFVH-----------------------CT-

T.vaginalis (XP_001581503): ------------------------------------------------------------

T.vaginalis (XP_001580012): FM---FVLLGFLLTSI----LA-----QIGRVVH-----------------------FK-

T.vaginalis (XP_001305124): FT---YTLLGFFLAAI----LA-----QIGRIIH-----------------------YK-

N.gruberi (XP_002682879): YG---MDDTAPLVPNDSVNSNRSTSMDIKRRSIYYTNEE-----DSDLDVIMSPAMGDQS

D.discoideum (XP_640187): GGGGIIRPRKPLPPVP-LGMIGKSSMAMTSNSSFGSNKPLNYIQQQQLQLQQQKVIGYQT

D.rerio (XP_696355): VDSTLSESHITKEPVI----NGSS----------------------QHDI----------

D.melanogaster (AFB77909): MQ---------LESME----QRSE----------------------Q-------------

M.musculus (NP_001032375): LDNCTVHVLTSQSPVR----QRPV----------------------R-------------

R.norvegicus (NP_001070668): IDNCTVHVLTSQSPVR----QRPV----------------------R-------------

B.taurus (XP_002694905): EDNCIIHDLTGHSAVQ----QRTS----------------------C-------------

C.lupus (XP_546782): TDSCTVHDLTGHTPVR----QRPV----------------------H-------------

A.thaliana (NP_182327): FGLGMLVALGNLV----------------NNSVF------LYLSEES-------------

O.sativa (NP_001172359): ------------------------------------------------------------

hPiezo1 (NP_001136336): -----------------------------------------PK-RAEP------------

hPiezo2 (NP_071351): SDESSEKREEE--------------------EEE-KEEFEEER-SREEKRS---------

E.histolytica (XP_649449): LS----SEEERE----------KE---ENE-ETTMIEKSEDIKEENKSIWN---------

E.histolytica (XP_655549): FT----SKTEED----------N-----SE-EEPFI-------DEKKSNNK---------

L.major (XP_001686914): QV-----AVGRSPRTIELDAICLAAAQQSGGSSPIAS--------------------RGG

L.infantum (XP_001469682): QV-----AVGRSPRTIELNAICAAAAQQSGASSPVAS--------------------RGG

T.cruzi (XP_819187): SF-----LVGRRPTAEEEKCLCKEGIGDALLREKLHD--------------------SQS

T.cruzi (XP_812333): SF-----LVGRRPTAEEEKCLCKEGIGDALLREKLHD--------------------SQS

L.major (XP_001686223): ------------------ENVCTQGDQD-DAQAEMQHRRRGAPVSKSQ------------

L.infantum (XP_001468509): ------------------ENVRTQGDQD-DAHAEMQHGQRGAPVSQSQ------------

L.braziliensis (XP_001564414): ------------------ENVHTEAGHD-DEQVAVQHSRRDAPAPQSQ------------

T.cruzi (XP_817508): TT-----ISFQDV--SSGEDQFRRGEHKNGLEEKVQHQQCSGRNSDALSRTISAVGSPGG

T.cruzi (XP_820998): TT-----ISFWDV--SSGEDQFRRGEHKNGLEDKVQHQQCSGRNSDALSRTISAVGSPGG

T.vaginalis (XP_001319509): --------------------PMIRTNPNHQNT----------------------------

T.vaginalis (XP_001582897): --------------------SSKKVNLDNESPE--------P------------------

T.vaginalis (XP_001581503): ------------------------------------------------------------

T.vaginalis (XP_001580012): --------------------PPKEAEGEQEEGMELRDIIDRPKKKKEKGP----------

T.vaginalis (XP_001305124): --------------------DDQE-ENEQENYNNLPDVVEKPKEEKKEFL----------

N.gruberi (XP_002682879): IHDSVFSDSIRDPTNVDMTELDKI---NDEADNNLGEVECEPQNTSKRVWV---------

D.discoideum (XP_640187): SA-SINQLDINDSFSIAFPSLMLL-------TSGIGKGYDTFRGKYGGTFS---------

D.rerio (XP_696355): -----------------------------------------GP-SGEA------------

D.melanogaster (AFB77909): -----------------------------------------EN-TTTSILD---------

M.musculus (NP_001032375): -----------------------------------------PR-LAEL------------

R.norvegicus (NP_001070668): -----------------------------------------PR-LAEL------------

B.taurus (XP_002694905): -----------------------------------------PR-LAEP------------

C.lupus (XP_546782): -----------------------------------------PR-LAEP------------

A.thaliana (NP_182327): -----------------------------------------SRSSNERSYV---------

O.sativa (NP_001172359): ------------------------------------------------------------

hPiezo1 (NP_001136336): -RE------------ASPLHSLGHLIMDQSYVCALIA------MM-----VWSIT---YH

hPiezo2 (NP_071351): -IK------------VHAMVSVFQFIMKQSYICALIA------MM-----AWSIT---YH

E.histolytica (XP_649449): -KITEYIKVIMKQI-VLIIKLLIQITIDCGLYVSLGI------MF-----CVGLV---DI

E.histolytica (XP_655549): -IIKEGLKQIYKFI-ITCLAVCLQIIVKGGVFISIIV------MF-----CVGLV---NI

L.major (XP_001686914): -L---------------TVEEVTQLLVSGPFYTYAVI-----LCMF----VLGTS---SV

L.infantum (XP_001469682): -L---------------TVEEVAQLLVSGPFYTYAVI-----FCMF----VLGTS---GV

T.cruzi (XP_819187): PL------------LSYYVHWAGILLMR--HAVLIAL-----LLLF----IVGSF---TY

T.cruzi (XP_812333): PL------------LSYYVHWAGILLMR--HAVLIAL-----LLLF----IVGSF---TY

L.major (XP_001686223): -----------MHVQSFYLAP------RSGWVFATAVISLATVCASVVLFGLLYS---SM

L.infantum (XP_001468509): -----------MRVQSFYPAP------RSGWVFATAIISLATVCTSVVLFGLLYS---SV

L.braziliensis (XP_001564414): -----------VRVQSFYLAP------RNGWVFATAVTSFATVCASGVLFGCLYT---SE

T.cruzi (XP_817508): TVAYLDFLVLVSKIESYRMQH------RNGVYVAASML-KQIICLNASTLSLI-------

T.cruzi (XP_820998): TVAYLDFLVLVSKIESYRMQH------RNGVYVAASML-KQIICLNTSTLSLV-------

T.vaginalis (XP_001319509): -ES-QVIKV---IA-----QYIITIIFI-----GLV--------------LLNAIFSSQY

T.vaginalis (XP_001582897): -EH-KWMNK---II-----QSVIVTIFL-----GNV--------------ILNAIFSSKF

T.vaginalis (XP_001581503): ------------------------------------------------------------

T.vaginalis (XP_001580012): -VV-KFIIKIAKIV-----TDVIVFIFKN-LSVGAVV-------------VLGITFGYQE

T.vaginalis (XP_001305124): -IV-KGIKLCFKWI-----KLIVVFIFKY-FTAAAII-------------ILALTYGYAE

N.gruberi (XP_002682879): -KIKLILQTIKIILFDYIWKVLLILFVRNSFYLSLIV------CY-----FADLT---SE

D.discoideum (XP_640187): -KITSSLKAIAEIT--------FLAIIGQSYRLALVG------LF-----FCGLT---SI

D.rerio (XP_696355): -TV------------DNPLRLVGRMVLQQSYICALIA------MM-----VWSIT---YH

D.melanogaster (AFB77909): -QI------------SYGFVSVGGFIYQNSYIFTNIL------MM-----AWSIV---YH

M.musculus (NP_001032375): -KE------------MSPLHGLGHLIMDQSYVCALIA------MM-----VWSIM---YH

R.norvegicus (NP_001070668): -KE------------MSPLHGLGHLILDQSYVCALIA------MM-----VWSIM---YH

B.taurus (XP_002694905): -KE------------TSPLHGLGHLIMDQSYVCALIA------MM-----VWSIT---YH

C.lupus (XP_546782): -QE------------TSTLHSLGHLIMDQSYVCALIA------MM-----VWSIT---YH

A.thaliana (NP_182327): -EADEETKVLVVATIAWGLRKCSRAIM-----LALIF------L----------I---AM

O.sativa (NP_001172359): ------------------------------------------------------------

hPiezo1 (NP_001136336): SWLTF---------------VLLLWACLIWTVRSRHQLAMLCS-----PCILLYGMTLCC

hPiezo2 (NP_071351): SWLTF---------------VLLIWSCTLWMIRNRRKYAMISS-----PFMVVYGNLLLI

E.histolytica (XP_649449): SFLGF--------------SFMIISICFVFIPPY---YSKKMW-----PLVIVFIILQII

E.histolytica (XP_655549): NIFGF--------------SFLVISISLIFIPIN---YCQKLW-----PIVIIYTLLIVL

L.major (XP_001686914): SMDLLH------------ATCLVVSLGVSVIGGS---AMLYRR-VRSVP--PM-CVAVVV

L.infantum (XP_001469682): SMDLLH------------ATCLVVSLCVSVIGGN---AMLYRR-VRSVP--PV-CVAVVV

T.cruzi (XP_819187): QMDILH------------AIVLLLFVLYFFLPDSMDDAWMFVV-IYLMLLTSFKLIFTFV

T.cruzi (XP_812333): QMDILH------------AIVLLLFVLYFFLPDSMDDAWMFVV-IYLMLLTSFKLIFTFV

L.major (XP_001686223): QVDASPAGAVLPGWATTPVAKSLLTVCVGIQVHSSNPKLCFCGATACLSVVSLTSWCLLL

L.infantum (XP_001468509): QVDSSLAGAVLPGWATTPAAKLLLTVCVGIQVDSSNPKLRFCGATACLSVVSLTSWCLLL

L.braziliensis (XP_001564414): QADLSSADSILPGWVTTPVAKLLLTVCFGVPVDSPNAKWQFCWTAACLTVVSLTSWCLLL

T.cruzi (XP_817508): -----------AMFAISMAVK-RLDIL-------NGVYLLFFIGLACVPWYMLYCWFIIV

T.cruzi (XP_820998): -----------AMFAISMAVK-RLDIL-------NGMYLLFFIGLACVPWYMLYCWFIIV

T.vaginalis (XP_001319509): HYLASD------------------------------------F-----PLFCMILILWFK

T.vaginalis (XP_001582897): NFLTYD------------------------------------I-----PLAFILLLMIFK

T.vaginalis (XP_001581503): --------------------------------------------------------MIFK

T.vaginalis (XP_001580012): NQYCFN------------------------------------A-----ICVIILITNVLG

T.vaginalis (XP_001305124): NQIIYH------------------------------------I-----VWLIALCISVLG

N.gruberi (XP_002682879): NADLLH-------------AIYLIFCAVYFIFPS---FARKTW-----ILLVLYCQFVIL

D.discoideum (XP_640187): NLLN---------------AGYMLFFIVFVISES---LASRFW-----MCLIIYAQMVLL

D.rerio (XP_696355): SWLTF---------------VLLLWSCVIWMLRTRQRFAAYCS-----PFILLYGLALCC

D.melanogaster (AFB77909): SWLTF---------------VLLLSANVLWMIPNQRKAMMRSS-----PFIVLYAEALLI

M.musculus (NP_001032375): SWLTF---------------VLLLWACLIWTVRSRHQLAMLCS-----PCILLYGLTLCC

R.norvegicus (NP_001070668): SWLTF---------------VLLLWACLIWTVRSRHQLAMLCS-----PCILLYGLTLCC

B.taurus (XP_002694905): SWLTF---------------VLLLWACLIWTVRSRHQLAMLCS-----PFILLYGLALCC

C.lupus (XP_546782): SWLTF---------------VLLLWACLIWTVRSRHQMAMLCS-----PFILLYGLALCG

A.thaliana (NP_182327): KPGFFH-------------AVYVIFFLMYLLSHN---INRKIR-----KSLILLCEVHFA

O.sativa (NP_001172359): ----------------------------------------------------------MP

hPiezo1 (NP_001136336): LRY----VWAMDLRPEL-------PT-----TL----GPVSLRQLGLE----HT------

hPiezo2 (NP_071351): LQY----IWSFEL-PEI-------KK-----VP----GFLEKKE----------------

E.histolytica (XP_649449): T-------------EI-I------V------QLPIIDISAIPSYIGLHKFNNKSTETSEF

E.histolytica (XP_655549): M-------------EM-I------V------QLDYIDVSSLPSYIGLYKFNDQTTHSVDI

L.major (XP_001686914): GVQ--LCYT-------VFAAGDE-KQRQQ--PHQLIPRGAQPSFLKAALGNY-VSL-TAL

L.infantum (XP_001469682): GVQ--LCYG-------VFAAGDE-KQRQQQQQRQLVPTGTQPSFLKATLCNY-VGL-TSL

T.cruzi (XP_819187): SEF--VTLS-------VYIAGSP-LQYA---TVGLVPLRS--------------------

T.cruzi (XP_812333): SEF--VTLP-------VYIAGSP-LQYA---TVGLVPLRS--------------------

L.major (XP_001686223): SRALGGCVQGAHKAASLVVAGSR-VTATSRWSR--VRVGWNTAL-RLFTGAVTSAP-YSL

L.infantum (XP_001468509): CRALGRCVQGAPKAASSVVAGAR-VTTTSRWSR--VRVGWNTAL-RLFAGVVTSAP-YSL

L.braziliensis (XP_001564414): CRVLGRCAHEAHEAASLTVASSF-VTSTSTWSR--VRVWWIMAV-RLLAGFITSAP-YSL

T.cruzi (XP_817508): A------YTALHVAVRFTV--------------------------MVYNASYHPND-TPS

T.cruzi (XP_820998): A------YTALHVAVRFTV--------------------------MVYNASYHPND-TPS

T.vaginalis (XP_001319509): LFY--NWVWAFLN--V-I----TFVS-----TSIYFINPLLKKKINI-------------

T.vaginalis (XP_001582897): LFY--RWAWTILN--L-I----TFAS-----TSIYFIAPLLKKPLNI-------------

T.vaginalis (XP_001581503): VFN--IWVWTVLN--L-I----TFGS-----TSIYFIAPLLKKKINI-------------

T.vaginalis (XP_001580012): LFH--RPIFYFIL--F-L-TGLIIIT-----ATVFETMPTDYFNSTVL------------

T.vaginalis (XP_001305124): IYY--RIIFEFIK--F-F-IGITIIT-----ATFFEANGLDFHN--ID------------

N.gruberi (XP_002682879): ALY----FFNVWYAPE-V------V------DLPYEIIGFNPSFITIEN--QISTSSFGV

D.discoideum (XP_640187): TLY----IWQLSWISSYE------N------DL--------TVLIGMTN-----------

D.rerio (XP_696355): LQY----VWAMDLETEL-------PQ-----HI----GSMSLHQLGLD----RA------

D.melanogaster (AFB77909): AQY----IYGMDLNNEEL------PT-----SVPT--AGINLQQIGFERPIENQ------

M.musculus (NP_001032375): LRY----VWAMEL-PEL-------PT-----TL----GPVSLHQLGLE----HT------

R.norvegicus (NP_001070668): LRY----VWAMEL-PEL-------PT-----TL----GPVSLHQLGLE----HT------

B.taurus (XP_002694905): LRY----VWAMDLRPEL-------PT-----AL----GPVSLRQLGLE----HT------

C.lupus (XP_546782): LRY----VWAMDLRPEL-------PT-----TL----GPVSLRQLGLE----HT------

A.thaliana (NP_182327): LLY----ILEIDLVSNSL------KQ-----EG--SASREVLFQLGLLR----SES----

O.sativa (NP_001172359): LL----------------------------------SILLLPFHHGLSN----KST----

hPiezo1 (NP_001136336): RYPCL----------DLGAMLLY--------TLTFWLLLRQFVKEKLLKWAESPAALT--

hPiezo2 (NP_071351): ---PG----------ELASKILF--------TITFWLLLRQHLTEQKALQEKE-ALLSE-

E.histolytica (XP_649449): DLKNT----------ILSGTLSR--------IILLVIAILQHVANKVGYKITTNTHN---

E.histolytica (XP_655549): KWDDT----------HLGELISK--------IILLFVSFLEYYANKIGVKVIHDESK---

L.major (XP_001686914): TWEDC----------APYL--CA-------QLVLMWC---SRYTPK--------------

L.infantum (XP_001469682): TWEDC----------APYL--CA-------QLVLMWC---SRYTPK--------------

T.cruzi (XP_819187): -RYEA----------IPYV--VA-------VFILTFLFLRIRVCRS--------------

T.cruzi (XP_812333): -RYEA----------IPYV--VA-------VFILTFLFLRIRVCRS--------------

L.major (XP_001686223): GWVGAAGVGQG---TALGLLSLA-------VVMV-VLLLSSS------VGRLQ-------

L.infantum (XP_001468509): GWVGAAGVGQG---TALGLLSLA-------VVMM-VLLLSSS------VGQLQ-------

L.braziliensis (XP_001564414): VWVCVAGVGQA---TTLGLLSLA-------VAMTTVLLFSSR------VGQVQ-------

T.cruzi (XP_817508): YWMEIIGIDIG---KDPFALLVY-------FLLLILAVFQLRISRHEASGKIT-------

T.cruzi (XP_820998): YWMEIIGIDIG---KDPFALLVY-------FLLLILAVFQLRISRHEASGKIT-------

T.vaginalis (XP_001319509): EWLF--DITVTKDPKVMFSKVWP-------FLVLFILTAILRYYYRQPPMIVVKFSK---

T.vaginalis (XP_001582897): PWLF--DGSVTTEPRNMLSKVWP-------FLILFLLTAIQRYYYRPPPKIVIKFSK---

T.vaginalis (XP_001581503): PWLF--DSTVTVNPNVMFSKVWP-------FLILFILTAIQRYYYRPPPQIVIKFSR---

T.vaginalis (XP_001580012): DYLEKTGLKRASGEADLPTYLWP-------YIVMVFLCVLITHMGKVK--VIYPSTV---

T.vaginalis (XP_001305124): DFLRKTGLAVPSG-YTLAGYLWP-------YMVILFLTVICTNMSDPI--FSLPPTG---

N.gruberi (XP_002682879): FWKGL---------------IWH--------IFILTLTSIQYHISNVKT--VGN------

D.discoideum (XP_640187): YYGSP----------LWVGLIWH--------IIIITFSIIQWNVNKLYQRGLFSSSS---

D.rerio (XP_696355): QYPCL----------RLGALLLF--------TLTFWLLLRQSVKDTFSRKKSLTVPLQ--

D.melanogaster (AFB77909): MRPCV----------PLIVKTAF--------VLMFWVTSRQFFKEKRDRRRDSTLADFIA

M.musculus (NP_001032375): RYPCL----------DLGAMLLY--------LLTFWLLLRQFVKEKLLKKQKVPAALL--

R.norvegicus (NP_001070668): RYPCL----------DLGAMLLY--------LLTFWLLLRQFVKEKLLKKRKAPSTLL--

B.taurus (XP_002694905): RYPCL----------DLGAMLLC--------TLTFWLLLRQFVKEKLLRRARAPVALT--

C.lupus (XP_546782): RYPCL----------DLGAMLLY--------TLTFWLLLRRFVKEKLLKKGKVPAALT--

A.thaliana (NP_182327): SWDFL----------EIALLACFCAIHNHGFEVLFSFSAIVRHTP-------------SP

O.sativa (NP_001172359): TKDFM----------KIGSIVCFCAVHSHGFKMLFALSAVLRHTP-------------ST

hPiezo1 (NP_001136336): -------------------------------------------EVTVAD---TEPTRTQT

hPiezo2 (NP_071351): ---VKIGSQENEEKDEELQDIQVEGEPKEEEEEEAKEEKQERKKVEQEE---AEEEDEQD

E.histolytica (XP_649449): ---VEIIK-----------------------------------------------YCFSK

E.histolytica (XP_655549): ---IEVFK-----------------------------------------------YSCAK

L.major (XP_001686914): ----------------------------------------W-------LTNWTHIAQCLA

L.infantum (XP_001469682): ----------------------------------------W-------LTNWTHIAQCLA

T.cruzi (XP_819187): ----------------------------------------WG--TAVSLHEWDRFCHSNT

T.cruzi (XP_812333): ----------------------------------------WG--TAVSLHEWDRFCHSNT

L.major (XP_001686223): -----R---------------------------------QRV--GHPRQRLWVLYTCMLL

L.infantum (XP_001468509): -----R---------------------------------QRV--GHPRQRLWVPYTCMLL

L.braziliensis (XP_001564414): -----R---------------------------------QRE--GNARQRLWSLYTCMLL

T.cruzi (XP_817508): -----I---------------------------------QEI----LQRMEWRRFTSCDF

T.cruzi (XP_820998): -----I---------------------------------QEI----LQRMEWCRYTSCDF

T.vaginalis (XP_001319509): -----------------------------------------------------------T

T.vaginalis (XP_001582897): -----------------------------------------------------------T

T.vaginalis (XP_001581503): -----------------------------------------------------------T

T.vaginalis (XP_001580012): -----------------------------------------------------------V

T.vaginalis (XP_001305124): -----------------------------------------------------------A

N.gruberi (XP_002682879): -------------------------------------------------------VNVNT

D.discoideum (XP_640187): ---SSSSSSNNNQ-----------------------NNNQNNQNNSY-E---DKFKNIPN

D.rerio (XP_696355): -------------------------------------------EVTTGE---S-TGRNES

D.melanogaster (AFB77909): PLQITVGSAG----------------------------------SSYLI---NDGKKTSK

M.musculus (NP_001032375): -------------------------------------------EVTVAD---TEPTQTQT

R.norvegicus (NP_001070668): -------------------------------------------EVTVSD---TEPTQTQT

B.taurus (XP_002694905): -------------------------------------------EVTVAA---TEPTRTQM

C.lupus (XP_546782): -------------------------------------------EVTVAD---TEPTRART

A.thaliana (NP_182327): PIGFSILKAGLNKSV--L--LSVY----------------SSPSSSYSQDNTTYERHIAS

O.sativa (NP_001172359): PVGFTILKAGLNKSV--L--LSVY----------------NSQNSRNGQADRST------

hPiezo1 (NP_001136336): LLQSLGELVKGVYAKYWIYVCAGM-------------------------FIVVSFAG-RL

hPiezo2 (NP_071351): IMKVLGNLVVAMFIKYWIYVCGGM-------------------------FFFVSFEG-KI

E.histolytica (XP_649449): IVDFWGIFICCFVIAFAAFFESINI-----------------------------------

E.histolytica (XP_655549): FIDFWGLFICCVVIAIAAFYEDINI-----------------------------------

L.major (XP_001686914): FRCSWARFIRTVYQVAGAVVLAWIV---------------------------LLL-PRSA

L.infantum (XP_001469682): FRCSWARFIRTVHQVAGVVALAWIA---------------------------LLL-PRSA

T.cruzi (XP_819187): A-RQWSMRV--TMTVATTVF-VALG---------------------------VFL-HYSF

T.cruzi (XP_812333): A-RQWSMRV--TMTVATTVF-VALG---------------------------VFL-HYSF

L.major (XP_001686223): C-L----------------WEVQMV---------------------------VL------

L.infantum (XP_001468509): C-L----------------WAVQMI---------------------------VL------

L.braziliensis (XP_001564414): F-L----------------WEVQMI---------------------------VL------

T.cruzi (XP_817508): FPQF----YRHFLELSTSLVLVMMA---------------------------LFI-PHNV

T.cruzi (XP_820998): FPQF----YRHFLELSTSLVLVMMA---------------------------LFI-PHNV

T.vaginalis (XP_001319509): VGVFVCLCLSCI-----------------------------------------Y---IQN

T.vaginalis (XP_001582897): VGVFLALCLSCI-----------------------------------------Y---IDN

T.vaginalis (XP_001581503): IGVFLSLCFSCV-----------------------------------------Y---IDN

T.vaginalis (XP_001580012): MFIFWALAILHL----------------------------------------AYLYVYGP

T.vaginalis (XP_001305124): TLVFWFLCLLHM----------------------------------------LYLYLFKF

N.gruberi (XP_002682879): SFDQWDMFTKEKNPYLHTFFEKLILVWDKIFLETELLNFLGTLASYFAVFLVIFL-QEEA

D.discoideum (XP_640187): FLLVFGDFIYRSVQQLSLPFCYLVIV-------------------------IVSI-FTKI

D.rerio (XP_696355): ILKVFGGLVMSLYAKYWIYVCGGM-------------------------FIMVSFAG-KL

D.melanogaster (AFB77909): FLKKAGDVIKNLLVRLWIWLLVLV-------------------------IFLCAITGENM

M.musculus (NP_001032375): LLRSLGELVTGIYVKYWIYVCAGM-------------------------FIVVSFAG-RL

R.norvegicus (NP_001070668): LLRSLGELVTGIYVKYWIYVCAGM-------------------------FIVVSFAG-RL

B.taurus (XP_002694905): LLRSLGELVRGIYAKYWIYVCAGM-------------------------FIVVSFAG-RL

C.lupus (XP_546782): LLQSLGELVTGLYAKYWIYVCAGM-------------------------FIVVSFAG-RL

A.thaliana (NP_182327): FLSAIGQKFLSMYRSCGTYIAFITI------------------------LISVYLVKPNY

O.sativa (NP_001172359): ------------------------------------------------------------

hPiezo1 (NP_001136336): VVYKIVYMFLF--LLCLTLFQVYYSLWRK-------LLKAFWWL--VVAYTML---VLIA

hPiezo2 (NP_071351): VMYKIIYMVLF--LFCVALYQVHYEWWRK-------ILKYFWMS--VVIYTML---VLIF

E.histolytica (XP_649449): ---ELM--MYIIALGVLVLIQIHFSNYKQ----IIRVILPIYA---IIFL--F---VLVV

E.histolytica (XP_655549): ---ELI--LYIIALAILVLLQTHFVYSKL----VIQFILPIYS---IIFF--V---VLLV

L.major (XP_001686914): SVTGLI--LLLFTVA---LLQ---HLRLH-------RLAYVWRRFFVVGYCGV---VLMC

L.infantum (XP_001469682): SVTGLI--LLLFTVA---LLQ---HLRLH-------RLAYVWRRFFVVGYCGV---VLMC

T.cruzi (XP_819187): IVEGFL--ALFLLLTISAAAF---SLSGA-------FLGVLWSVA--CVYSGI---VVVV

T.cruzi (XP_812333): IVEGFL--ALFLLLTISAAAF---SLSDA-------FLGVLWSVA--CVYSGI---VVVV

L.major (XP_001686223): ------------------------------------------------------------

L.infantum (XP_001468509): ------------------------------------------------------------

L.braziliensis (XP_001564414): ------------------------------------------------------------

T.cruzi (XP_817508): FIAGFA--LLFLLYFSLGAYS---KLAES-------V-----TLIALGAYNVA---VLLI

T.cruzi (XP_820998): FIAGFA--LLFLLYFSLGAYS---KLAES-------V-----TLIALGAYNVA---VLLI

T.vaginalis (XP_001319509): SIFTVLYQLLII----GNLFLYK------------------FHSVLMIINSASTSCHLLT

T.vaginalis (XP_001582897): SIFTVLYQLLIV----ANLFLYK------------------WNYVLMVINTILTSLQIVV

T.vaginalis (XP_001581503): SIFTVLYQLLIV----ANLFLYH------------------WNYILIVINTIISASQIVV

T.vaginalis (XP_001580012): NIFSVIYVI-------TGIFIFYSKV----------LNKVLLTKIAIVVSGISVSLHLTM

T.vaginalis (XP_001305124): NVFSLAYLV-------AGVWIFYCQV----------LNKVQLLKVAIMVSGFCVSLHQGL

N.gruberi (XP_002682879): SFIGFIFLMIVMACFCLNVLSVHNSNSTKAGYRLFQFFVGLWYV--FVFYSTL---VFVV

D.discoideum (XP_640187): SLINIVYMATVFLC----LLIHHISANGS------IHIKRFWII--IILSQGV---VLVA

D.rerio (XP_696355): VAYKIVYMLLF--LLCMCLYQVYYSLWRR-------LLKAFWWM--VVAYTMV---VLIA

D.melanogaster (AFB77909): TGFRICYMALF--LFFLLVFQSSSKAWVK-------IMYGFWLF--LIFYAMS---ILIL

M.musculus (NP_001032375): VVYKIVYMFLF--LLCLTLFQVYYTLWRK-------LLRVFWWL--VVAYTML---VLIA

R.norvegicus (NP_001070668): VVYKIVYMFLF--LLCLTLFQVYYTLWRK-------LLRVFWWL--VVAYTML---VLIA

B.taurus (XP_002694905): VVYKIVYMLLF--LLCLILFQVYYSLWRK-------LLKAFWWL--VVAYTML---VLVA

C.lupus (XP_546782): VVYKIVYMLLF--LLCLTLFQVYYSLWRK-------LLKVFWWL--VVAYTML---VLIA

A.thaliana (NP_182327): VSFGYIFLLLL--------WITGRQLFE-------ETKRRLWFP--LKAYAVL---VFMF

O.sativa (NP_001172359): ------------------------------------------------------------

hPiezo1 (NP_001136336): VYTFQFQDFP--------------------------------AYWRNLTG----------

hPiezo2 (NP_071351): IYTYQFENFP--------------------------------GLWQNMTG----------

E.histolytica (XP_649449): RYIIQMDVII--------------------SDNSQSESPLIDSFGDIFTK----------

E.histolytica (XP_655549): RYVSQFRPDS---------------------VYSNNNDIYINTFWDIFTG----------

L.major (XP_001686914): MLTAEFQP-------VQ------------------------PRLWRLLRA-LG---CPPG

L.infantum (XP_001469682): MLTAEFEP-------VQ------------------------PRLWQLLRT-LG---CPPG

T.cruzi (XP_819187): TSVCQFTQLA---DLIR------------------------EHVFQRLCGHDGDGSGNST

T.cruzi (XP_812333): TSVCQFTQLA---DFIW------------------------EHVFQRLCGHDGDGSGNST

L.major (XP_001686223): -----LPP-------VQ------------------------HSLKV-------ASVWAR-

L.infantum (XP_001468509): -----LPP-------VQ------------------------NSLKA-------TSVWTR-

L.braziliensis (XP_001564414): -----LPS-------IQ------------------------QSLKT-------TSVWAK-

T.cruzi (XP_817508): SALYHVPA-------IS------------------------NSFAERL---SNVTVCQKI

T.cruzi (XP_820998): SALYHVPA-------IS------------------------NSFAERL---SNITVCQKI

T.vaginalis (XP_001319509): ILLFSYEQIK---PYIE-----------------------KKELILKLFG----------

T.vaginalis (XP_001582897): LAAFGYDEIK---NKFT-----------------------NSGFYFKFFG----------

T.vaginalis (XP_001581503): IATFGYPEIK---SIVT-----------------------HKAFFFKFFG----------

T.vaginalis (XP_001580012): LMFSEFEWTR---NYLM-----------------------SITFNKKFT-----------

T.vaginalis (XP_001305124): LMLSEYPQTR---NFLC-----------------------AL------I-----------

N.gruberi (XP_002682879): EYAYQFPSLY---AYIQSILPEVERAKQLIREHELDKSIEIPSYYG---Y----------

D.discoideum (XP_640187): RYIMQFNQVS---HWLNSIFPKSN-------------------------Y----------

D.rerio (XP_696355): IYTFQFEDFP--------------------------------GYWGNFTG----------

D.melanogaster (AFB77909): IYTYQFDKFD--------------------------------TYWSDYLN----------

M.musculus (NP_001032375): VYTFQFQDFP--------------------------------TYWRNLTG----------

R.norvegicus (NP_001070668): VYTFQFQDFP--------------------------------TYWRNLTG----------

B.taurus (XP_002694905): VYTFQFQDFP--------------------------------AYWRNLTG----------

C.lupus (XP_546782): VYTFQFQDFP--------------------------------MYWRNLTG----------

A.thaliana (NP_182327): IYCL--SSFVSLQLWLS-------------------------GFIDLYFY----------

O.sativa (NP_001172359): ------------HGLVS-------------------------KFVKLYPD----------

hPiezo1 (NP_001136336): FTDEQLGDLGLEQFSVSEL-FSSILV--PGFFLLACILQLHYFHRP------F--MQLTD

hPiezo2 (NP_071351): LKKEKLEDLGLKQFTVAEL-FTRIFI--PTSFLLVCILHLHYFHDR------F--LELTD

E.histolytica (XP_649449): M---NQSELGLVKYNSLWERLLGLSP--NIIVIVICFVESRLLITL------P--KYLKN

E.histolytica (XP_655549): M---SQVEVGCLKYNSLWERLKGFLP--NILVIAVCAIESRLLITV------P--RYLKE

L.major (XP_001686914): SEGRCAQDIGL-PADSAMW-LTPLSI--PWWLIIVLATATARLHPL------PSS-----

L.infantum (XP_001469682): SEGRCAQDIGL-PADSAMW-LTPLSI--PWWLIIVLATATARPHPL------PSS-----

T.cruzi (XP_819187): NARRCEQESGLYPVREDMP-LTLFLA--PWFLAFATAMLHRAVKHV------RET-----

T.cruzi (XP_812333): NARRCEQESGLYPVREDMP-LTLFLA--PWFLAFATAMLHRAVKRV------RET-----

L.major (XP_001686223): -HGRWLAAVGAVPAASP-----DSML--RWRIVQAIGLWWAGTEFVCYHAAAPATSQVAS

L.infantum (XP_001468509): -HGRWLAAVGAVPAASH-----SSML--RWRIVQAIGLWWAGTEFVCYHTAAPATSQVTS

L.braziliensis (XP_001564414): -HGRWLAAVGAVPAASH-----NSMP--CWRIIAAIGLWWAGAEFVCYYGSVYGTSPVAR

T.cruzi (XP_817508): TVQRCALDVGVSQNNQFGS-MSLNLL--PWYLAAALSVA------LCRH-----------

T.cruzi (XP_820998): TVERCALDVGVSQNNQFGS-MSLNLL--PWYLAAALSVA------LCRH-----------

T.vaginalis (XP_001319509): I----I--------ENPNV-FKDIML--PNIILIL--YSVFASISRKYD---------EP

T.vaginalis (XP_001582897): I----Q--------TKPDV-IKDQMS--PIIILIM--YTFFGSIARKYD---------TT

T.vaginalis (XP_001581503): I----S--------EHPDI-TRHQMI--PIIILIL--YIFFGSIARKYD---------VD

T.vaginalis (XP_001580012): M----Q--------DLIDL-RTPQTPGLEAALLAV--TMF--CISLTYM---------NT

T.vaginalis (XP_001305124): P----S--------NIIDI-STPTSPGSESVLLAV--TQF--TISLTYR---------NF

N.gruberi (XP_002682879): L---TPKSIGLYKYQYKNVSL---LP--FTIIAIL-------TIVQ------L--KYFQF

D.discoideum (XP_640187): I---SLSDIGLRNYSSSDR-FIELFG--CSSILVVCVFQLTVFFS---------------

D.rerio (XP_696355): FTEQQLADMGLETFKLSEL-FTSIVI--PGFFLLACILQLHYFHKP------F--MKITD

D.melanogaster (AFB77909): VSATLQKDIGLKRYQTKDL-FLHLVS--PTIIVILTVIQVHYFHKR------F--IASLQ

M.musculus (NP_001032375): FTDEQLGDLGLEQFSVSEL-FSSILI--PGFFLLACILQLHYFHRP------F--MQLTD

R.norvegicus (NP_001070668): FTDEQLGDLGLEQFSVSEL-FSSILI--PGFFLLACILQLHYFHRP------F--MQLTD

B.taurus (XP_002694905): LTDEQLGDLGLEQFSVSEL-FSSILV--PGFFLLACILQLHYFHRP------F--MQLTD

C.lupus (XP_546782): LTDEQLGDLGLEQFSVSEL-FSSILI--PGFFLLACILQLHYFHRP------F--MQLTD

A.thaliana (NP_182327): L--------G---YNSKAPLLDNVW----ESLAVLIVMQLYSYER---------------

O.sativa (NP_001172359): L--------G---FDPEASLLMNVW----QSLAVLVVMQLYSYER---------------

hPiezo1 (NP_001136336): MEHVSLPGTRLP-RWAHRQDAVSGTPLLRE---------------EQQE-----HQQQQQ

hPiezo2 (NP_071351): LKSIPSKEDNTIYRLAHPEGSLPDLTMMHLTASLEKPEVRKLAEPGEEKLEGYSEKAQKG

E.histolytica (XP_649449): VRQIRREIRKRKQ---D-----YD------------------------------------

E.histolytica (XP_655549): VEEMYLAELKKKRGVQH-----FDREALQYSF-----------NRFEQETRLANEINEEK

L.major (XP_001686914): ----------LPSSLAA------------------------------------------A

L.infantum (XP_001469682): ----------LPSSLAA------------------------------------------A

T.cruzi (XP_819187): -----------PPRWARQPDG----------------------SGGLVGMTVEQQ--EEQ

T.cruzi (XP_812333): -----------PPRWARQPDG----------------------SGGLVGMTVEQQE-EEQ

L.major (XP_001686223): LKGGQKLRALLLRELAQRDENQWSRAVNQQRAA----------HGKLQQLRLERDIYLYR

L.infantum (XP_001468509): FKGGQKLRALLLRGLAQRDENQWSRAVNQQRGA----------HCKLQQLRLERDAYLHR

L.braziliensis (XP_001564414): FNGGRERRALLRRGLAQRDGKQWSDAIDQQRAA----------HGKLQELRLERDAYLFR

T.cruzi (XP_817508): ------YFGVLGRGINRREEGNDD------EGA----------SVRMEEMQN-----MPD

T.cruzi (XP_820998): ------YFGVLGRGINRREEGNDD------EGA----------SVRMEEMQN-----MPD

T.vaginalis (XP_001319509): ------------------------------------------------------------

T.vaginalis (XP_001582897): ------------------------------------------------------------

T.vaginalis (XP_001581503): ------------------------------------------------------------

T.vaginalis (XP_001580012): N-----------------------------------------------------------

T.vaginalis (XP_001305124): V-----------------------------------------------------------

N.gruberi (XP_002682879): VIGRRRQRIKTVQEQEE--------------I-----------RKKEQEQTIPNQPQQQP

D.discoideum (XP_640187): -IGQQQQQQQQQQQQQQ-----QQQQQQQQ-Q-----------QQQQQQQQQQQQQQQQQ

D.rerio (XP_696355): LENVSPIHRK---------RGIDNPDLVQS---------------TEEV-----------

D.melanogaster (AFB77909): Q--QPLAGGSAQQ-------KPTETTAL---------------EPAPSKRRGS-----AG

M.musculus (NP_001032375): LEHVPPPGTRHP-RWAHRQDAVSEAPLLEH---------------QE-------------

R.norvegicus (NP_001070668): LEHVPPPGTRRL-RWAHRQDTVSEAPLLQH---------------QE-------------

B.taurus (XP_002694905): PEHRPLPGACTP-RWVPGRDAVSRTPLLQQ---------------EE-------------

C.lupus (XP_546782): LEHVPPPGACPP-RWAHRQDVVSGTPLLQE---------------EE-------------

A.thaliana (NP_182327): ----RQ------------------------------------------------------

O.sativa (NP_001172359): ----RQ------------------------------------------------------

hPiezo1 (NP_001136336): EEEEEEE-DSRDEGL---GVATPHQATQVPEGA-AKWGLVAERLLELAAGFSDVLSRVQV

hPiezo2 (NP_071351): DLGKDSE-ESEEDGE---EEEES-EEEEETSDLRNKWHLVIDRLTVLFLKFLEYFHKLQV

E.histolytica (XP_649449): ---------------------------------QNPENHEIEEEIKVPK----KVKEISD

E.histolytica (XP_655549): NLTKVELISTSQEGNKILKSKKDKT------NKQNKYLNDEEKVQCIPK----VICEISD

L.major (XP_001686914): P----------ATGVPEASTPW-FW--RALVWWPKV------------L-SD----VLSI

L.infantum (XP_001469682): P----------ATGIPEASTPW-FW--RALVWWPKV------------L-SN----VLSI

T.cruzi (XP_819187): P----------KRGEQSEGEQR-VVP-VECVSLPCL------------E-SD----DQ--

T.cruzi (XP_812333): P----------KRGEQSEGEQR-VVP-VECVSLPCL------------E-SD----DQ--

L.major (XP_001686223): PS-----RAGSN------------------DWWR--------H----AQ-RG----RSDR

L.infantum (XP_001468509): PS-----RAGSN------------------HWWR--------H----AQ-RD----GSHR

L.braziliensis (XP_001564414): SS-----RGSSGSGPRRTRAPQQHVA----SWCR--------N-HQYAQ-HD----RQHM

T.cruzi (XP_817508): PS-----ISN-RRGAREEEEEEEEVA-MVVEVAREGNHAMREE-RDFLE-ND----GRQR

T.cruzi (XP_820998): PS-----ISN-RRGAREEEEEE---------VARERNHAMRGE-RDFLE-ND----GRQR

T.vaginalis (XP_001319509): ------------NG----NTKIPHFL-----------ELIINNLWAITIKFS------FY

T.vaginalis (XP_001582897): -------------E----DGKIPKWI-----------TLIIDNCWAIILKFS------FY

T.vaginalis (XP_001581503): -------------E----NHEL---M-----------TFLIENLWAIVLKFS------FY

T.vaginalis (XP_001580012): ------------PL----QKRPNIWI-----------QGIREEFKAALRSLF------FY

T.vaginalis (XP_001305124): ------------TG----YNKKGIYA-----------HGILEEIKAALRSFY------FY

N.gruberi (XP_002682879): PT--------TTTGPTHIHEHTPSLMDEIVETIQESSHVKIEKQIIFSEQIDSLLLECFL

D.discoideum (XP_640187): QL------------------------NTSNNNNQNNNNLIIKKYT--------FFDSLLY

D.rerio (XP_696355): --------RPEEEEL---IVDQDDYPSEADEVIPSKWGLVMDRLMVLSRKFSDTLTHVQS

D.melanogaster (AFB77909): SLRKSQ--GPSAEAA---PGATTDFETSVRDLVRISF-------RKIKNKSEYIFKNFKD

M.musculus (NP_001032375): ----EEE-VFREDGQ---SMDGPHQATQVPEGTASKWGLVADRLLDLAASFSAVLTRIQV

R.norvegicus (NP_001070668): ----EEE-VFRDDGQ---SMDGPHQTTQVPEGTASKWGLVADRLLDLASSFSAVLTRIQV

B.taurus (XP_002694905): ----EEE-VPRDEGL---GTASPHQVTQVPEGK-FKWGLVAERLLDLATGFSDVITRVQV

C.lupus (XP_546782): ------------DGL---STACPHQAMQVTE-A-NKWGLVAERLLDLASGFSDVLTRVQV

A.thaliana (NP_182327): ------------SG------------------------------HYIPGQSSLLHPGVFG

O.sativa (NP_001172359): ------------NS------------------------------DKNFGVSDASESGLLG

hPiezo1 (NP_001136336): FLRRLLELHVFKLV-----ALYTVWVALKEVSVMNLLLVVLWAFALPYP-RFR-------

hPiezo2 (NP_071351): FMWWILELHIIKIV-----SSYIIWVSVKEVSLFNYVFLISWAFALPYA-KLR-------

E.histolytica (XP_649449): LFMRFLSIYLPHIS-----I-------------------ICAIVFAAYPTIYTTPEIETW

E.histolytica (XP_655549): LIMRFLAIYIPHIS-----V-------------------ICAIIIAAYPIVDSTFEHQTW

L.major (XP_001686914): -------------AILAGMVHAAL----QRPSILAALYLVGPGL----G------VFPCW

L.infantum (XP_001469682): -------------AILAGMVHTAL----QRPSILAALYLVGPGL----G------VFPCW

T.cruzi (XP_819187): -------------RFIRHMLRLLA----VMDKLGDWIAAFGSDY----G------GILVW

T.cruzi (XP_812333): -------------RFIRHMLRLLA----VMDKLGDWVAAFGSDY----G------GILVW

L.major (XP_001686223): SA------HAPFGSNVSHCCWLCRDD----------------------------------

L.infantum (XP_001468509): SV------HAPFGSNVSHCCWLCRDG----------------------------------

L.braziliensis (XP_001564414): AG------HAPFGTSVSHCFWMDRDD----------------------------------

T.cruzi (XP_817508): MGLFFCSYTATRGTFIGRCVPMAFAISRYLYLVVTFIREAGTAQ----G------KLMVW

T.cruzi (XP_820998): MGLFFCSYTATRGTFIGRCVPMAFAISRYLYLVVTFIREAGTAQ----G------KLMVW

T.vaginalis (XP_001319509): -----LFWS----------SIFLVVIVSKGVSIVGSLMIMV-LG---VLKVTGVRTNAI-

T.vaginalis (XP_001582897): -----FFWS----------AIFLMVIVSKGVSIVGAVMLMV-LG---IVKVLGCRTKQI-

T.vaginalis (XP_001581503): -----FFWS----------AIFLVVIVSKGVSIVGAVMLIV-LG---IIKVTGHSAKKS-

T.vaginalis (XP_001580012): -----ISWL----------LMFFFSISNDYPTIIKCVISILFLL---GSKMARFFNKMV-

T.vaginalis (XP_001305124): -----ISWI----------FMFFFSIANDYPTVIKLLISVFFLL---GSKVARLFNKMV-

N.gruberi (XP_002682879): VMKRFCVMHTPKLL-----IIFLFLASNFNPNIIGLIYLVLVLVFAPAPVIAS----KIW

D.discoideum (XP_640187): IVKRICYLHGPKFV-----LWMVFAISIAEYNFFNFIYLIMIVISMSF-------KKGTY

D.rerio (XP_696355): FIWRVLELHIVKIV-----AFFVVWVALLEPSAMNLVLVVLWSFAMPYG-RFR-------

D.melanogaster (AFB77909): VFWRFLELHIMKAV-----YIAAFVCSVSEVCVLHIIFVGFCVLGATSRKAVQ-------

M.musculus (NP_001032375): FVRRLLELHVFKLV-----ALYTVWVALKEVSVMNLLLVVLWAFALPYP-RFR-------

R.norvegicus (NP_001070668): FVRCLLELHVFKLV-----ALYTVWVALKEVSVMNLLLVVLWAFALPYP-RFR-------

B.taurus (XP_002694905): LVRRLLELHVFKLV-----ALYTVWVALKEVSVLNFLLVVLWAFALPYP-RFR-------

C.lupus (XP_546782): LLRRLLELHILKLV-----ALYTVWVALKEVSVMNLLLVVLWAFALPYP-RFR-------

A.thaliana (NP_182327): FFERFLAWHGQKIL-----FAALFYASLSPISVFGFVYLLGLVICTTFPKSSSIPSKSF-

O.sativa (NP_001172359): FLRRLLIWHSEKIL-----SVTVFYACLSSISLSGLIYLLGLIMFSILPKVSRIPSKVY-

hPiezo1 (NP_001136336): -----------------------PMASCLSTVWTCVIIVCKMLY-QLKVVNPQEYSSNCT

hPiezo2 (NP_071351): -----------------------RLASSVCTVWTCVIIVCKMLY-QLQTIKPENFSVNCS

E.histolytica (XP_649449): DFLHFL-L-FVSTLIALLMKKGF-NAACIPLLWVCTLSSLILIIPNFHTINDF-------

E.histolytica (XP_655549): DCLHFL-L-FITTMICLLSKKGF-NAACAPLLWVCTISMIILIVPNFRTFNQL-------

L.major (XP_001686914): TLGV---AAVHAALQCVYQLWFSPGWLDAQ------------------------------

L.infantum (XP_001469682): TFGV---AAVHAALQCVYQLWFSPGWLDAQ------------------------------

T.cruzi (XP_819187): VSLFLAATYRFTAIGSVYMLFFLLDLTHISVLVYCVLHILV------LYTYKFSFVPEIT

T.cruzi (XP_812333): VSLFLAATYRFTAIGSVYMLFFLLDLTHISVLVYCVLHILV------LYTYKFSFVPEIT

L.major (XP_001686223): -------------------------PQHLPTSVGGGRRDG-------HECNPTLF-HNGP

L.infantum (XP_001468509): -------------------------PQHLPTSVGGGRRDG-------HECNPTLF-HNGP

L.braziliensis (XP_001564414): -------------------------SQHLPSYVS-SRSDS-------HEYYLTLL-HNKP

T.cruzi (XP_817508): VSLAYASLSNFSLLGAAFMALFLLDSRGMPVVAVAVAQMAL------NYVYPFFFIPEAP

T.cruzi (XP_820998): VSLAYASLSNFSVLGAAFMTLFLLDSRGMPVVAVAVAQMAL------NYVYPFFFIPEAP

T.vaginalis (XP_001319509): ---------------------------------------GILLYIIFI--A-DVL-----

T.vaginalis (XP_001582897): ---------------------------------------GVVLYAIFI--I-DVL-----

T.vaginalis (XP_001581503): ---------------------------------------GVFLYVIFM--L-DVL-----

T.vaginalis (XP_001580012): ---------------------------------------GALLYTNTVYIVIEVF----L

T.vaginalis (XP_001305124): ---------------------------------------KYQLLFNLVFLFVEMF----F

N.gruberi (XP_002682879): FLIALYSG-FIMTIKYIYQHPFFTNGLCDSAL-------GAIV-----------------

D.discoideum (XP_640187): RIGSF--------------------LLFYSQLWVLTQL--AALLPTV-------------

D.rerio (XP_696355): -----------------------AMASCLSTIWVCVIIVCKMLY-QLSVVNPAEYSNNCT

D.melanogaster (AFB77909): -----------------------VVISRLISFIVTVIVLSKMIY-QIEYLSHSQHNVVCS

M.musculus (NP_001032375): -----------------------PMASCLSTVWTCIIIVCKMLY-QLKIVNPHEYSSNCT

R.norvegicus (NP_001070668): -----------------------PMASCLSTVWTCIIIVCKMLY-QLKIVNPHEYSSNCT

B.taurus (XP_002694905): -----------------------PMASCLATVWTCIIIVCKMLY-QLKVVNPHEYASNCT

C.lupus (XP_546782): -----------------------PMASCLSTVWTCIIIVCKMLY-QLKVVSPHEYSSNCT

A.thaliana (NP_182327): ---LIYTG-FLVSAEYLFQLWGMQAQMFPGQKYA--------------------------

O.sativa (NP_001172359): ---LVYTG-LLATSEYLFQMLCEPAQMCPGQQFH--------------------------

hPiezo1 (NP_001136336): EPFPNSTNLLPTEISQSLLYRGPVD----PANWFGVRKGFPNLGY--------------I

hPiezo2 (NP_071351): LPNENQTNIPFNELNKSLLYSAPID----PTEWVGLRKSSPLLVY--------------L

E.histolytica (XP_649449): -------------INQSGIFKTEE-TKEWFYKLIGLKTCYENNERTELCETFDGIKYPKS

E.histolytica (XP_655549): -------------IDQYNVFGSTQ-TKIWIYSLIGIKTCFDDVLTAPKCVSYDELKYPYC

L.major (XP_001686914): -----------------TRLGVS------PAQLLGLWRPLSAMGSTPNL--------PST

L.infantum (XP_001469682): -----------------TRLGVS------AAQLLGLWKPSSAMGSAANL--------PST

T.cruzi (XP_819187): ----------------YSIYNVP------LAAVIGLEKK----GGNALF-----------

T.cruzi (XP_812333): ----------------YSIFNVP------LASVIGLDKK----GGNTLF-----------

L.major (XP_001686223): -------------------APSS------AAGV---------------------------

L.infantum (XP_001468509): -------------------APSS------AAGV---------------------------

L.braziliensis (XP_001564414): -------------------FSAS------NARV---------------------------

T.cruzi (XP_817508): -------------------LSVS------SKRFFGLQKTQNGERPS-IL--------P--

T.cruzi (XP_820998): -------------------LSVS------SKRFFGLQKTQNGERPS-IL--------P--

T.vaginalis (XP_001319509): --VVTAI-----DITNTS--DS---VKK-VFKLIGPLVNTSTAKF---------------

T.vaginalis (XP_001582897): --FVTTV-----AIVNAP--EK---VTK-VVNLIGPLCDTVLAKF---------------

T.vaginalis (XP_001581503): --FVTTV-----ASVNVP--SK---VTK-IVNLIGPLCDTPLAKL---------------

T.vaginalis (XP_001580012): DIFPKLT-----DKDQTP-YTS---TRK-WCEYIGLYFVDGDKGQ-------GDRNYSMT

T.vaginalis (XP_001305124): EIFPNYI-----EKH-----------RT-ILEYVGLYFVNGEYTI-------ANRNASMT

N.gruberi (XP_002682879): -------------QHQVENFGRSTGVCTWL-GYIGLEDARSTEGH---------HIGNVL

D.discoideum (XP_640187): -----------------QSFNSDK----FFMDWVGLRQ----------------PNVSTP

D.rerio (XP_696355): APMVNETSLEPDEVLNSTLYRASVD----PASWFGVRKDDTVLGY--------------V

D.melanogaster (AFB77909): DNR-------------------TAN----NAEWIGLTKADKVTGG--------------L

M.musculus (NP_001032375): EPFPNNTNLQPLEINQSLLYRGPVD----PANWFGVRKGYPNLGY--------------I

R.norvegicus (NP_001070668): EPFPNNTNLQPLEISQSLLYRGPVD----PANWFGVRKGYPNLGY--------------I

B.taurus (XP_002694905): EPFPNSTNLQKTEIRQSLLYRGPVD----PANWFGVRKGFPNLGY--------------I

C.lupus (XP_546782): EPLPNSTNLQKMEIKQSLLYRGPVD----PANWFGVRKGFPNLGY--------------I

A.thaliana (NP_182327): ---------------------E-------LSFYLGLRVYEPGFWG--------------I

O.sativa (NP_001172359): ---------------------G-------LSVFLGLKHYDAGFWG--------------V

hPiezo1 (NP_001136336): QNHLQV----LLLLVFEAI-----V-YRRQEHYRRQH-----------------------

hPiezo2 (NP_071351): RNNLLM----LAILAFEVT-----I-YRHQEYYRGRN-----------------------

E.histolytica (XP_649449): WELLKEYIIILFIIILSRT------SYFWGEKYKKKK-----------------------

E.histolytica (XP_655549): WDLIKDFLVIFFLIIITRT------SFFWGRIYKKKE-----------------------

L.major (XP_001686914): LVVAAAPLLVGVAQSLQL-QCALL--ARRRERDTV-------------------------

L.infantum (XP_001469682): LVVAAAPLLVGAAQFLQL-QCALL--ARWRERNTV-------------------------

T.cruzi (XP_819187): -EHVIGPVIVFFVMLLRIHTCGANREVRRRQRTEG-------------------------

T.cruzi (XP_812333): -EHVIGPVIVFFVMLLRIHTCGANREVRRRQRTEG-------------------------

L.major (XP_001686223): ----------------------------HAHDTCWTAAAVGSS-----------------

L.infantum (XP_001468509): ----------------------------HVHDTRWTAAAVGSS-----------------

L.braziliensis (XP_001564414): ----------------------------HGNNACGTADALRSS-----------------

T.cruzi (XP_817508): ------PILACAMAVFYMHT-EKDRAARHQEDALRQA-HRLKN-----------------

T.cruzi (XP_820998): ------PILACAMAVFYMHT-EKDRTARHQEDALRQA-HRRNN-----------------

T.vaginalis (XP_001319509): AN-----VIVCLCAFLNMS-------HFRVTEISEYV-----------------------

T.vaginalis (XP_001582897): AN-----VLVCLCAFINMS-------HFDTKEIHPII-----------------------

T.vaginalis (XP_001581503): AN-----VLVCLCAFLNMS-------HFDAKQINPII-----------------------

T.vaginalis (XP_001580012): WQ-----LLFILVIIVNMI-------AEHPEQTDP-------------------------

T.vaginalis (XP_001305124): WQ-----LLFIFVSILNII-------AEHPEPTDP-------------------------

N.gruberi (XP_002682879): WS----SLLVFVASLLQKV------TYRWERSMKSRG----LYDEGCLFI-EAEV-----

D.discoideum (XP_640187): WDAVKLNLAIILVISIQQT------SYWWNKEIQNEK----LEIKKKKQL-KKQQQQQRK

D.rerio (XP_696355): KDHLLV----LMLLVFEAT-----V-YRHQIHHYRQQ-----------------------

D.melanogaster (AFB77909): MSLLRTYIIYMVIVTMHAV-----ISLRQLQMRVKIG-----------------------

M.musculus (NP_001032375): QNHLQI----LLLLVFEAV-----V-YRRQEHYRRQH-----------------------

R.norvegicus (NP_001070668): QNHLQI----LLLLVFEAV-----V-YRRQEHYRRQH-----------------------

B.taurus (XP_002694905): QNHLQI----LLLLVFEAM-----V-YRSQDYHHRRH-----------------------

C.lupus (XP_546782): QNHLQV----LLLLVFEAI-----V-YRRQEHHRRQH-----------------------

A.thaliana (NP_182327): ESGLRGKVLVVAACTLQYN-----V-FRWLERTSGLTVIKGKYEEPCPLFVSAEDTTA-S

O.sativa (NP_001172359): EYGLRGKVLVIVACTIQYN-----V-FHWLDLMPTSLLHEGKWEEPCQLFISGDTSSN-A

hPiezo1 (NP_001136336): ------------------------------------------------------------

hPiezo2 (NP_071351): ------------------------------------------------------------

E.histolytica (XP_649449): ----------------------------------------------------------SL

E.histolytica (XP_655549): ----------------------------------------------------------RL

L.major (XP_001686914): ------------A--------A-CPGRV---SMWCR--LQRTC-----------------

L.infantum (XP_001469682): ------------A--------T-CSGRG---RMWCR--LQRTC-----------------

T.cruzi (XP_819187): ------------P-------------ES---SNWKS--VL--C-----------------

T.cruzi (XP_812333): ------------P-------------ES---SNWKS--VL--C-----------------

L.major (XP_001686223): ------------SLYSNASVYDCLAGIR---APWQH--SENASA-QPSPASA--EGDSAA

L.infantum (XP_001468509): ------------SLYSNTSVYDCLAGMR---ASWQY--SENASA-QPSPASA--ESDSAA

L.braziliensis (XP_001564414): ------------PLYSNSSVCGCLPGQP---VPWQY--SEITST-LPSLTIA--ESESTV

T.cruzi (XP_817508): ------------PPYHTPSTRD---GMR---AFFRS--EEIQTPYVPFVASR--ENRER-

T.cruzi (XP_820998): ------------PPYHNPSARD---GMR---AFVRS--EEIQTPYVPFVASR--ENRER-

T.vaginalis (XP_001319509): -----------------KLVGNSL-S----------------------------------

T.vaginalis (XP_001582897): -----------------TNIGDCL-A----------------------------------

T.vaginalis (XP_001581503): -----------------TYVGNCL-A----------------------------------

T.vaginalis (XP_001580012): ------------------KFDGSL-GMRLYRAFYMM------------------------

T.vaginalis (XP_001305124): ------------------KFDKCL-GMRLYRAFYSM------------------------

N.gruberi (XP_002682879): ---------------NVKSKND----PM---ASFAQK-----LRPSSSADKRKSKARNRN

D.discoideum (XP_640187): LEEHEEEYEEEEDQFGNKKNND----KL---SLLSNDSIEIILDDGNNNNNNNNNNNNNN

D.rerio (XP_696355): ------------------------------------------------------------

D.melanogaster (AFB77909): ------------------------------------------------------------

M.musculus (NP_001032375): ------------------------------------------------------------

R.norvegicus (NP_001070668): ------------------------------------------------------------

B.taurus (XP_002694905): ------------------------------------------------------------

C.lupus (XP_546782): ------------------------------------------------------------

A.thaliana (NP_182327): VSSSN----------GENPSST----DH---ASISMKQGEATSNSWPFF--SPRGNQGAG

O.sativa (NP_001172359): RDNNK----------DSHSSNR----FS---SLFSKVQGLIGSSSSSSLS-SGSTCQTSE

hPiezo1 (NP_001136336): QL----------------------------APLPAQAVFASGTRQQLDQ-DLLGCLKYFI

hPiezo2 (NP_071351): NL----------------------------TAPVSRTIFHDITRLHLDD-GLINCAKYFI

E.histolytica (XP_649449): VL-------------------FHYTD---ISK-------------EKEG-YSINKFKYYI

E.histolytica (XP_655549): TL-------------------FRYID---LEY-------------DLKK-YSWNKVKYYI

L.major (XP_001686914): -------------------------------------------------ATHLYMLL---

L.infantum (XP_001469682): -------------------------------------------------ATHLYVLL---

T.cruzi (XP_819187): -------------------------------------------------T-QLFFYAGYE

T.cruzi (XP_812333): -------------------------------------------------T-QLFFYAGYE

L.major (XP_001686223): VLV-------------------TAT----AAVVLSESAAEPLKLHNLEVERPLYMWQGWA

L.infantum (XP_001468509): VLI-------------------TTT----TAVVVSESAAEPLKRHDPEVEPSLYMWQGWA

L.braziliensis (XP_001564414): GLI-------------------AAT----ATVVLSESAAEPLKHHDSEVASSPYMWQGWS

T.cruzi (XP_817508): ----------------------------------TREVYEPPARLITLKQQLIFMWNYFR

T.cruzi (XP_820998): ----------------------------------TREVYEPPAKFVTLKQHLIFMWNYFR

T.vaginalis (XP_001319509): ------------------------------SILLSAV------------ELTLSIMAIQE

T.vaginalis (XP_001582897): ------------------------------SILISGV------------EFTLVVMAIQE

T.vaginalis (XP_001581503): ------------------------------SILISGV------------EFTLVIMAIQE

T.vaginalis (XP_001580012): -L---------------------------HNWLPVFV------------NISLCISTLYN

T.vaginalis (XP_001305124): -L---------------------------HNWLPVFV------------NISLCISTLYN

N.gruberi (XP_002682879): TI-----YISREEFEKMNFDEDHPTD---LTEEENFKSFYKRKIIKFLR-KLFSSFKYYC

D.discoideum (XP_640187): NNNNNN-NNNNNNNNNNNNNNNNNNN---QSNNENNE----NNNNSKKE-NLKKRLFWYI

D.rerio (XP_696355): QR----------------------------SPPPIPVIFPQATRDTLDQ-GLLHCIKYML

D.melanogaster (AFB77909): AL----------------------------NAPPTKLLFPNIIRADAEK-DLVGLVKYLL

M.musculus (NP_001032375): QQ----------------------------APLPAQAVCADGTRQRLDQ-DLLSCLKYFI

R.norvegicus (NP_001070668): QQ----------------------------APLPAQALCADGTRQRLDQ-DLLSCLKYFI

B.taurus (XP_002694905): QL----------------------------APLPAQAVCAEGTRQRLDR-DLPSCLKYYV

C.lupus (XP_546782): QL----------------------------APLPAQAVCADGTRQRLDQ-DLLSCLKYFV

A.thaliana (NP_182327): FLHPKTGGSESGSSRKFSFGHFWGSIKESHRWNRRRILALKKERFETQKNLLKIYLKFWI

O.sativa (NP_001172359): PVQNET--SGSDEGKRYSFSKIWGMSKESHKWDKRKIISLRRERFETQKTTFKCYMKFWM

hPiezo1 (NP_001136336): NFFFYKFGLEICFLMAVNVI-GQRMNFLVTLHGCWLV---------AILTRRHRQAIARL

hPiezo2 (NP_071351): NYFFYKFGLETCFLMSVNVI-GQRMDFYAMIHACWLI---------AVLYRRRRKAIAEI

E.histolytica (XP_649449): SNFFSVFGPLIVLIIILLCCAFHSNDIICIFYIGLA----------ISGIRLPYRVITKI

E.histolytica (XP_655549): SNFFSYFGPLYILLVLMICICLHANDFMAILYIIIS----------LVAVICPHKILTKM

L.major (XP_001686914): ---------------LWVLLYLTQKIALGWGVGVLIMMVWISAEVRSCGVLQRRR-----

L.infantum (XP_001469682): ---------------LWVLLYLTQKIALGWGVGVLITVVWISAEVRSCGVLQRRR-----

T.cruzi (XP_819187): G----------LVLTLLLVLACSSHSCIGAFVGLAVLPLLLATG---RQQLHRVS-----

T.cruzi (XP_812333): G----------LVLTLLLVLACSSHSCIGAFVGLAVLPLLLAMG---RQQLHRVS-----

L.major (XP_001686223): R-----------------------------------------APTRSPCKD---------

L.infantum (XP_001468509): R-----------------------------------------APTRSPCRD---------

L.braziliensis (XP_001564414): R-----------------------------------------APTRSSGRG---------

T.cruzi (XP_817508): TRFFLDLNFELTVLVASLGLALTPDRVTGLVFFVEFILMWLIGRVRSTTEIR--------

T.cruzi (XP_820998): TRFFLDLNFELTVLVASLGLALTPDRVTGLVFFVELLLMWLIGRVRSTIEIR--------

T.vaginalis (XP_001319509): SNILSIFGVVLVFVLLIKP----------------------------NLSLCSARLIT--

T.vaginalis (XP_001582897): NNILNIFGVVLVLIILLHP----------------------------RMSLRGARIIT--

T.vaginalis (XP_001581503): NNILNLFGIIVVFIILVHP----------------------------KVSLLEARIIT--

T.vaginalis (XP_001580012): PTVFGFFSLLVLIIVQFKP----------------------------KVLQRGAVWIS--

T.vaginalis (XP_001305124): PTIFGLFSYIILNIVQFKP----------------------------QVLKRGALLIT--

N.gruberi (XP_002682879): NHFYDDYGFEISLFTLLLGSLSLIQTLWGFIYLIVFGVCFFTG-KNSTILRRGWIF---L

D.discoideum (XP_640187): SNFYELYGLECVFLVLAFAL-FWRLNILGMIYLIIIAVGLNID----------KRNLHKL

D.rerio (XP_696355): NYSFYKFGLEICFLMTVNVI-GQRMNFLVIIHGCWLV---------AIMVRRRRANIATI

D.melanogaster (AFB77909): NFGFYKFGIEISLIALVSTI-TYRQDIVAVVYALWLV---------VLLLLR-RSQCAKI

M.musculus (NP_001032375): NFFFYKFGLEICFLMAVNVI-GQRMNFMVILHGCWLV---------AILTRRRREAIARL

R.norvegicus (NP_001070668): NFFFYKFGLEICFLMAVNVI-GQRMNFMVILHGCWLV---------AILTRRRREAIARL

B.taurus (XP_002694905): NFFFYKFGLEVCFLAAVNVI-GQRMNFMVILHGCWLV---------AILTRRRREAIARL

C.lupus (XP_546782): NFFFYKFGLEICFLMAVNVI-GQRMNFMVILHGCWLV---------AILTRRRRAAIARL

A.thaliana (NP_182327): ENMFNLYGLEINMIALLLAS-FALLNAISMVYIALLAACV----------LLRRRVIQKL

O.sativa (NP_001172359): ENLFKLREILSRNCGLFLL--FSLLR---SYYS----STL----------LFGRK-----

hPiezo1 (NP_001136336): WPNYC--LFLALFLLYQYLLCLGM--------------PPALC---IDYPWRWSRAVPMN

hPiezo2 (NP_071351): WPKYC--CFLACIITFQYFICIGI--------------PPAPC---RDYPWRFKGA-SFN

E.histolytica (XP_649449): IPIIK--LLLVILLII--NSIWTI--------------PLTGGPIKNWQKWNNWN-WDID

E.histolytica (XP_655549): IPVVE--IILLILVII--NSVWTI--------------PLEPL-DTQWEIWKKWD-WDID

L.major (XP_001686914): WLFAACGSATSVLMVLAYLLHW--------LHTVF----------------PWWSA----

L.infantum (XP_001469682): WLFGACGSATAVLMALAYLLHW--------LHTVF----------------PWWSA----

T.cruzi (XP_819187): YS-------FPLLLLLSLMVLWQYALLLNAAHRLFPAIADYSEAKETDGMWEYWVF----

T.cruzi (XP_812333): YS-------FPLLLLLSLMVLWQYALLLNAAHRLFPAIADYSEAKETDGMWEYWVF----

L.major (XP_001686223): ------------------------------------------------------------

L.infantum (XP_001468509): ------------------------------------------------------------

L.braziliensis (XP_001564414): ------------------------------------------------------------

T.cruzi (XP_817508): YAVL-----QESLIVIA-------------LG----------------------------

T.cruzi (XP_820998): YAVL-----QESLIVIV-------------LG----------------------------

T.vaginalis (XP_001319509): -------IILTIIL--------GY--------------VLTFKIITINTKNDWMS-----

T.vaginalis (XP_001582897): -------IILNFVL--------GY--------------VLALLIVKFNKHNTWYD-----

T.vaginalis (XP_001581503): -------IILNLVL--------GY--------------VLAFIIIKFKGKNEWYD-----

T.vaginalis (XP_001580012): -------VLFNFCFLFQYVIWLGP--------------PSRFYFISDALDNDWGD-----

T.vaginalis (XP_001305124): -------FLFNCCFMFQYLVWLGL--------------PKSPYYFT-GLPTRWLE-----

N.gruberi (XP_002682879): VMVVE--INMLILFFFRLQIVRSM--------------QFYEA-MMNIP-----------

D.discoideum (XP_640187): IYVSA--LLAPTILIQ-YLLILVV--------------PTKE----NSYPWLDHP-FFLN

D.rerio (XP_696355): WSKYC--LFLVVFMIYQYVLCVGI--------------PPALC---IDYPWRWRSTVTIH

D.melanogaster (AFB77909): WGVFQ--AFFAISILTQYIVLVGL--------------PPSSC---LVFPWDE---GPFG

M.musculus (NP_001032375): WPNYC--LFLTLFLLYQYLLCLGM--------------PPALC---IDYPWRWSKAIPMN

R.norvegicus (NP_001070668): WPNYC--LFLTLFLLYQYLLCLGM--------------PPALC---IDYPWRWSQAIPMN

B.taurus (XP_002694905): WPNYC--LFLALFLLYQYLLCLGV--------------PPALC---IDYPWRWSRAVPMN

C.lupus (XP_546782): WPNYC--LFLSLFLLYQYLLCLGI--------------PPALC---IDYPWRWSQAIPMN

A.thaliana (NP_182327): WPVVV--FLFASILAIEYVATWNSFL------------PSDQAPSETSV--HCHDCWSIA

O.sativa (NP_001172359): -----------------------------------------ECPGYT----------VSM

hPiezo1 (NP_001136336): SALIKWLYL----PDFFRAPNSTNLISDFLLLLCASQQWQV----F-SAERTEEW-----

hPiezo2 (NP_071351): DNIIKWLYF----PDFIVRPNPVFLVYDFMLLLCASLQRQI----F-EDENKAAV-----

E.histolytica (XP_649449): QTLRKYILLVPT-NSGIKL-----LLS---------------------------------

E.histolytica (XP_655549): YSLRKYTLLMPV-EQGKKL-----LVI---------------------------------

L.major (XP_001686914): --LAMYPYLF-GGSINEDKT-----MTALQSVCCAAAAACVVLRVSSGAPRGNES-EHLL

L.infantum (XP_001469682): --LATYPYLF-GGSTNEDKM-----MAELQSVCCAAAAACAVLRVSSGAPRDKES-EHLL

T.cruzi (XP_819187): --TSRYHTLL-GCVLGI-----------------------MLLHTSRQQSGDTAVSFSML

T.cruzi (XP_812333): --TSRYHTLL-GCVLGI-----------------------MLLHTSRQQSGDTAVSFSML

L.major (XP_001686223): -----------G-GDGDEDD------TGVRHSSCGSYSN--------GEHRAYRW-SPPP

L.infantum (XP_001468509): -----------E-GDGDEDD------NDVRHSSCGACSN--------GEHRAYWW-GPPP

L.braziliensis (XP_001564414): -----------G-VEGNEDD------AGVRHRSCGAYSN--------GEHSVNRC-ALPL

T.cruzi (XP_817508): ---GLYFIRL-GLPKGLVGV------SILHPEECNAWD------------HYCGC-GLPL

T.cruzi (XP_820998): ---GLYFIRL-GLPKGLVGV------SILHPEECNAWD------------HYCGC-GLPL

T.vaginalis (XP_001319509): -----YLLL--H---DVKMV---EIFYIFLTMF-ALCLFCE----YGS------------

T.vaginalis (XP_001582897): -----YLLL--T---NIKMI---EVFYIFLTMF-AFSLFCE----FGT------------

T.vaginalis (XP_001581503): -----YFLL--T---NVKMI---EIFYIFLTMI-VFSLFCE----FGR------------

T.vaginalis (XP_001580012): -----FLSL--R---NIETE---ALLTNMVTAF-MLTFYLQ----FRE------------

T.vaginalis (XP_001305124): -----FFSL--T---NIEIP---ALLTNMVTAF-VLTFYLQ----FRE------------

N.gruberi (XP_002682879): -IFSKYIILNLTSTSGGVISNTYIVVILYLMMFFMALQARV----F-FKGMYARL-----

D.discoideum (XP_640187): HKTIDNLLL-------LSIPDRYVLVIDFLVLFFSMLLFKQ------RNGYY-LY-----

D.rerio (XP_696355): SALVKWMYL----PDFYTVPNSKNLISDFLLLMCASQQWTV----F-DNEKKEEW-----

D.melanogaster (AFB77909): EGIQRWAML----PGALHFNHVPKLIFDFIVLVILNRQKSI----F-CIEQRYAS-----

M.musculus (NP_001032375): SALIKWLYL----PDFFRAPNSTNLISDFLLLLCASQQWQV----F-SAERTEEW-----

R.norvegicus (NP_001070668): SALIKWLYL----PDFFRAPNSTNLISDFLLLLCASQQWQV----F-SAEQTEEW-----

B.taurus (XP_002694905): SALIKWLYL----PDFFSTPNATNLISDFLLLLCASQQWQV----F-SAERMEEW-----

C.lupus (XP_546782): SALIKWLYL----PDFFRTPNSTNLISDFLLLLCASQQWQV----F-SAERTEEW-----

A.thaliana (NP_182327): ALYFKFCR---ECWLGVRVDDPRTLISYFVVFM-LACFKL-------RADHI--------

O.sativa (NP_001172359): TL--K-----------FIVANAGKIQGFSLHIA-QNVGWL-------RSDRF--------

hPiezo1 (NP_001136336): Q--------------------------------------------------RMAGVNT--

hPiezo2 (NP_071351): R--------------------------------------------------IMAGDNV--

E.histolytica (XP_649449): -------------------------------------RIF-----DALCIFLI---NRLM

E.histolytica (XP_655549): -------------------------------------KVF-----DCLFIFLI---NRIS

L.major (XP_001686914): CHS--HTPSFLDWLRRHSGQLRENHEDISASLSEMPPDEIRYFFRDVAGVFHCDEEVA--

L.infantum (XP_001469682): RHS--HSPSFLDWLRRHSRQLRENREDISASLSEMPPDEIRYFFRDVAGVFHCDEEAA--

T.cruzi (XP_819187): RSQFNGHVTLVNFVR----------AASVGSLQLLPRDTFDLVV----------------

T.cruzi (XP_812333): RSQFNGHVTLVSFVR----------AASVGSLQLLPRDTFDLVV----------------

L.major (XP_001686223): SHSSAHSPYTFSVTTSI---PLPGHSR-------------------LHG-----------

L.infantum (XP_001468509): SHSSAHSPYTFSVTTSS---PRTGHSR-------------------LHGAAYANATP---

L.braziliensis (XP_001564414): QHSCAHYPYAFSVASSG---PRASHSR-------------------LRGVTYVDVSL---

T.cruzi (XP_817508): HHFVLMFAL--LVLRRC---MRQNVAEVR------------ATTLCVEGAFELGELLR--

T.cruzi (XP_820998): HHFVLMFAL--VVMRRC---MRQNVAKVR------------ATILCVEGAFELGELLR--

T.vaginalis (XP_001319509): ----------------------------------------------------LTGSLGPT

T.vaginalis (XP_001582897): ----------------------------------------------------LNVSVGRS

T.vaginalis (XP_001581503): ----------------------------------------------------VDAAVGRS

T.vaginalis (XP_001580012): ----------------------------------------------------LAMNYDAG

T.vaginalis (XP_001305124): ----------------------------------------------------LAISYDIA

N.gruberi (XP_002682879): QHIEVSQPEKLPK--------RER-GEIVSSFLS-RTQKL-----DNALIVHLDTSSLIK

D.discoideum (XP_640187): KDFELHQQQQLNQ--------QLN-QQ-----------QH-----DSLKS--IASSNS--

D.rerio (XP_696355): M--------------------------------------------------VMGGENR--

D.melanogaster (AFB77909): ND-------------------D-----------------------------YPGGSNR--

M.musculus (NP_001032375): Q--------------------------------------------------RMAGINT--

R.norvegicus (NP_001070668): Q--------------------------------------------------RMAGVNT--

B.taurus (XP_002694905): R--------------------------------------------------HMAGVNT--

C.lupus (XP_546782): Q--------------------------------------------------HMAGVNT--

A.thaliana (NP_182327): -----------------------------SS---------------------F--SESST

O.sativa (NP_001172359): -----------------------------SG---------------------F--SDSDT

hPiezo1 (NP_001136336): -----DRL---EPL----R-G-EPNPVPNFI-HCRS---YLDMLKVAVFRYLFWLVLVVV

hPiezo2 (NP_071351): -----EICMNLDAA----S-FSQHNPVPDFI-HCRS---YLDMSKVIIFSYLFWFVLTII

E.histolytica (XP_649449): YNYHEGHSHDIYKF----I-S-----PYYFPKEIKT---LFDT-----------------

E.histolytica (XP_655549): FHFREGHVHEIYKF----I-S-----PPYFPEVSRN---IFDGVHRVLLKYFNLICGIII

L.major (XP_001686914): -E-E----ADT------SPLSRGPAPRRNGGVVVSVVRLM--PL--VACGSVAVGSATAS

L.infantum (XP_001469682): -E-G----ADT------SPLSTGPAPPSKGGVVVSVARRV--PL--VACGSVAVGSATAS

T.cruzi (XP_819187): ------------------PSFVESLPPSCLGRVTTFMVFAVSYL--CPVAFLSFAAGVMS

T.cruzi (XP_812333): ------------------PSFVESLPPSCLGRVTTFMVFAVSYL--CPVAFLSFAAGVMS

L.major (XP_001686223): --------A--------------------------------------GCSSARWPSMIHG

L.infantum (XP_001468509): -R-QDGSGG--------------------------------------GCSSARWPSMIHG

L.braziliensis (XP_001564414): -R-QNGSSG--------------------------------------GSSNKCWPPTTCG

T.cruzi (XP_817508): -R-KEKEVEDCRRLLRSDIESVLPAPSRDI---IRNPRTLFDLL--IAVSCACLPAAVFA

T.cruzi (XP_820998): -R-KEKEVEDCRRLLRSDIESVLPAPSRDI---IRNPRTLFDLL--IAVSCACLPAAVFA

T.vaginalis (XP_001319509): F------LTAYYTT------------------ICSI-------------------VAVII

T.vaginalis (XP_001582897): F------LTAYFPM------------------ILSV-------------------AATVI

T.vaginalis (XP_001581503): F------LTTYFPT------------------IVSV-------------------ATTVI

T.vaginalis (XP_001580012): FNDLPEFLKGVIKF------------------IVSN---IFEIIE---------AVAIAI

T.vaginalis (XP_001305124): FDELPETIKQIVLF------------------ICAN---IFEIIE---------AIAISV

N.gruberi (XP_002682879): RNIETGKIQKIKDF----T-T---------PDQQKK---LWNWIKLIVYRFFCFIVLIVM

D.discoideum (XP_640187): SSQKPLHPKNFFKF----N-SSIDGGDDDFTKEPRS---WSNELRYMIIRYSSQVILIVI

D.rerio (XP_696355): -----DNP---DPM----E-NMLFNPAPNFI-NCRS---YLDMAKILVFRYMFWFVLSVV

D.melanogaster (AFB77909): -----SVIADIAQL----GRVPFDNPTHDFCSYIRN---YSDILKNGVLCGFYWFTLAVV

M.musculus (NP_001032375): -----DHL---EPL----R-G-EPNPIPNFI-HCRS---YLDMLKVAVFRYLFWLVLVVV

R.norvegicus (NP_001070668): -----DHL---EPL----R-G-EPNPIPNFI-HCRS---YLDMLKVAVFRYLFWLVLVVV

B.taurus (XP_002694905): -----DRL---EPL----R-G-EPNPVPNFI-HCRS---YLDMLKVAVFRYFFWLVLVVV

C.lupus (XP_546782): -----DRL---ELP----L-G-EPNAVPNFI-YCRS---YLDMLKVAVFRYLFWLVLVVV

A.thaliana (NP_182327): YHQMKSQRKNSFVW----R---------DLSFETKSMWTVLDYLRLYCYVHLLDVVLILI

O.sativa (NP_001172359): YHQMMSQRKNALVW----R---------DLSLETKSFWTFLDYIRLYAYCHLLDIVLALI

hPiezo1 (NP_001136336): FVTGATRISIFGLGYLLACFYLLLFGTALLQRDTRARLVLWDCLILYNVTVIISKNMLSL

hPiezo2 (NP_071351): FITGTTRISIFCMGYLVACFYFLLFGGDLLLKPIKSILRYWDWLIAYNVFVITMKNILSI

E.histolytica (XP_649449): ----ITRKDMISYIYIGMMFY-----VIFKGN-FLKQRKTWIIIQGINAIILVLQNIIV-

E.histolytica (XP_655549): MLLAVNRKDIISYVYVGMTMY-----IVFKGK-FLRERIQWKILQVLNFFILIVQNIFV-

L.major (XP_001686914): ------PPCVLRAALLLAGLCLA----VRHPRLHWSCWRWWRLTVALYALLPLIALVAAC

L.infantum (XP_001469682): ------PPCVLRAALLLAGLCLA----ARHPRLHWSFWRWWRLTVALYALLPLIALVAAC

T.cruzi (XP_819187): ------QPSVINLLLIIGGLLQV----AQMEMLHWTFFSLWPFITMAYWFLITLTVVCNA

T.cruzi (XP_812333): ------QPSVINLLLIIGGLLQV----AQLEMLHWTFFSLWPFIAMAYWFLITLSVVCNA

L.major (XP_001686223): TSVDISAV------GHAS---------SFAEEAAH-----------------------QR

L.infantum (XP_001468509): TSVDISAV------DHAS---------SFAEEAAH-----------------------HR

L.braziliensis (XP_001564414): MPPGVSVA------DGAS---------SFEEAAAR-----------------------QC

T.cruzi (XP_817508): FVQGVLMASLLGVVEIAASLWIL----AFKRYLAWRWHKVWPFFILFLFLT-FLPQLLAR

T.cruzi (XP_820998): FVQGVLMASLLGVVEIAASLWIL----AFKRYLAWRWHKVWPFFILFLFLT-FVPQLVAR

T.vaginalis (XP_001319509): SLTGTTY-----LILVHSLLLIINLVYTSHNKYF--HIYSFKVAIFYCFGNLLMKSFRPL

T.vaginalis (XP_001582897): SFYAHTY-----LTMVHTIMLCVNLLFSFYTNDF--HLRSFTVVIWYAFAIILIKSLRPL

T.vaginalis (XP_001581503): SFYAHTY-----LTFIHTCLLLINQGFNYYNSRF--HPKSFNVVIWYAYIILLVKALRPL

T.vaginalis (XP_001580012): SIFIPTF-DGLFFTILISILFYMTLLHDFDN------HKTISIHLWGLFLVIAGRLLSRI

T.vaginalis (XP_001305124): SIFIPTV-DGIFFFLLVNTLFFATLLFEFDS------HKTISIHLWGIFLVILARWISRC

N.gruberi (XP_002682879): FIDAAIDSN---VIKLGQMILCLFYLNLFNRL-YWRTVPFWRGIGIFYYLTFVIDAIYQI

D.discoideum (XP_640187): FLAGTAECDILSCFYVFFSVYVLFSGNAHSR----KWSYLWKSLHIYNWLVLMAQIIFQV

D.rerio (XP_696355): FVTGATRISVFGLGYLMACFFFLLFGTKLLTKPSRTRLVMWDCLIIYNVAVIISKNILSI

D.melanogaster (AFB77909): FLAGTNIADLLALGYLIGAFIFLWQGSDFYLRPIHTIIFRWKWLLAFNVANILIKTSFQM

M.musculus (NP_001032375): FVAGATRISIFGLGYLLACFYLLLFGTTLLQKDTRAQLVLWDCLILYNVTVIISKNMLSL

R.norvegicus (NP_001070668): FVTGATRISIFGLGYLLACFYLLLFGTTLLQKDTRAQLVLWDCLILYNVTVIISKNMLSL

B.taurus (XP_002694905): FITGATRISVFGLGYLLACFYLLLFGTSLQQKDTRARLVLWDCLILYNVTVIVSKNMLSL

C.lupus (XP_546782): FVTGATRVSVFGLGYLLACFYLLLFGTSLLQKHTRTRLVLWDCLILYNVTVIISKNMLSL

A.thaliana (NP_182327): LITGTLEYDILHLGYLAFALVFARMRLEI----LKKKNKIFRFLRVYNFVLIIFSLAYQS

O.sativa (NP_001172359): AITGTLEYDVLHLGYLGFALVFFRMRLEI----LKRKNKIFKYLRMYNFALIVLSLAYQS

hPiezo1 (NP_001136336): LACVFVEQMQ------------------------TGFCWVIQ------------------

hPiezo2 (NP_071351): GACGYIGTLV------------------------HNSCWLIQ------------------

E.histolytica (XP_649449): ----LSNMLN------YF-TL----NQ-------SNNKIT--------------------

E.histolytica (XP_655549): ----LSSLLT------YF-KD----DE-------ETKK-T--------------------

L.major (XP_001686914): PCV---RDAIP-----------------------K-------------------------

L.infantum (XP_001469682): PCV---RDAIP-----------------------K-------------------------

T.cruzi (XP_819187): PYV---RQFIL-----------------------E-------------------------

T.cruzi (XP_812333): PYV---RQFIL-----------------------E-------------------------

L.major (XP_001686223): PPL---SPEAS-----------------------A-------------------------

L.infantum (XP_001468509): PSV---WPEDS-----------------------A-------------------------

L.braziliensis (XP_001564414): LSL---PPKDQ-----------------------A-------------------------

T.cruzi (XP_817508): LPL---TPPLM-----------------------T-------------------------

T.cruzi (XP_820998): LPL---SPPLT-----------------------T-------------------------

T.vaginalis (XP_001319509): RIF---------------------------------------------------------

T.vaginalis (XP_001582897): RFI---------------------------------------------------------

T.vaginalis (XP_001581503): RFI---------------------------------------------------------

T.vaginalis (XP_001580012): PYF---STNG--------------------------------------------------

T.vaginalis (XP_001305124): PYF---TSLG--------------------------------------------------

N.gruberi (XP_002682879): PIVLLGDPIN------WG-NE----ND-------TSG-WV--------------------

D.discoideum (XP_640187): AVILYFQFKFNSNQMFEGHNHNHNHNHSSSSSSSSSGSIIDILSSQSSAIGIGGGNSSGS

D.rerio (XP_696355): LACVFVMEMQ------------------------KNFCWVIQ------------------

D.melanogaster (AFB77909): AGCLFMTQLT------------------------KDCCWLVH------------------

M.musculus (NP_001032375): LSCVFVEQMQ------------------------SNFCWVIQ------------------

R.norvegicus (NP_001070668): LSCVFVEQMQ------------------------SNFCWVIQ------------------

B.taurus (XP_002694905): LSCVFVEQMQ------------------------SSFCWVIQ------------------

C.lupus (XP_546782): LSCVFVEQMQ------------------------SNFCWVIQ------------------

A.thaliana (NP_182327): PFV---GNFN------------------------DGKCETVD------------------

O.sativa (NP_001172359): PYF---GQFS------------------------SGKCDQID------------------

hPiezo1 (NP_001136336): -------------LFSLVCTVKGYYDP------KEMMDRDQDCLLPVEEAGIIWDSVCFF

hPiezo2 (NP_071351): -------------AFSLACTVKGYQMP------A----ANSPCTLPSGEAGIIWDSICFA

E.histolytica (XP_649449): ---------TFITILFQFLRAFGLNTF------------AGK-----EFY---INLVIFF

E.histolytica (XP_655549): ---------QGINLVLQIFETFGLYMH------------AGE-----PFV---FNVVILF

L.major (XP_001686914): -------------LPPWVGLLIGWSVDA--------SHVDGN-----ALVFSHWHVMLFL

L.infantum (XP_001469682): -------------LPLWMGLLIGWSVDA--------SHVDGN-----ALVFSHRHVMLFL

T.cruzi (XP_819187): -------------HYGLSE-VLG-------------LLQAGK-----PFQLGSLHVLLLF

T.cruzi (XP_812333): -------------HYGLSE-VLG-------------LLQAGK-----PFQLGSLHVLLLF

L.major (XP_001686223): -------------HPEEVRAEH--AP----------------------------------

L.infantum (XP_001468509): -------------RPEEVRAEH--AP----------------------------------

L.braziliensis (XP_001564414): -------------RPEQMRAEY--TT----------------------------------

T.cruzi (XP_817508): -------------HAENVMLALGFSPWS-----------RGT---------TVGQAMVLF

T.cruzi (XP_820998): -------------HAENLMLALGFSPWT-----------RGT---------TVGQAMVLF

T.vaginalis (XP_001319509): ---------------TQSETEIDRI-------------------FGFRGKDNFQWIFVYW

T.vaginalis (XP_001582897): ---------------PQTNSVFDQA-------------------MGTMKADNLQWCVCFW

T.vaginalis (XP_001581503): ---------------PQTETTFDKA-------------------FGTIKVDNLNWCVCFW

T.vaginalis (XP_001580012): -------------YAHYVQEAFGLP-------------------FRGKSSTEYLWIILFA

T.vaginalis (XP_001305124): -------------MGKWISSLFDLP-------------------FRASSSSEVWWIILFA

N.gruberi (XP_002682879): ---------K------NVCLLFGLKAR------------GLE-----SFT---TEIIVVC

D.discoideum (XP_640187): DSSYEVIENSLPTELYNIAVVFGFKIE------------TGP-----LSISTISDVIIMV

D.rerio (XP_696355): -------------LFSLVCTVKGYYDP------ESVC--NKDCSLPVEEAGIIWDSICFF

D.melanogaster (AFB77909): -------------MLGITCTSNVLTEQIMLPEEAELALKPGECPKITHQVVLLWDTICFA

M.musculus (NP_001032375): -------------LFSLVCTVKGYYDP------KEMMTRDRDCLLPVEEAGIIWDSICFF

R.norvegicus (NP_001070668): -------------LFSLVCTVKGYYDP------KEMKTRDRDCLLPVEEAGIIWDSICFF

B.taurus (XP_002694905): -------------LFSLVCTVKGYYDP------KEMLGRDQDCLLPVEEAGVLWDSVCFL

C.lupus (XP_546782): -------------LFSLVCTVKGYYDP------KEMLSRDRDCLLPVEEAGVLWDSICFL

A.thaliana (NP_182327): ----------------YIYEVIGFYKY------------D--YGFRITARSALVEIIIFM

O.sativa (NP_001172359): ----------------YIYEIIGFYKY------------D--YGFKITSRSAFVEIVIFL

hPiezo1 (NP_001136336): FLLLQRRVFLSHYYL-HVRADL----QATALLASRGFALYNAANLKSIDF------HRRI

hPiezo2 (NP_071351): FLLLQRRVFMSYYFL-HVVADI----KASQILASRGAELFQATIVKAVKA------RIEE

E.histolytica (XP_649449): VLLVRVALF--------------KQLPE--------------------------------

E.histolytica (XP_655549): VMLIRSAVF--------------RQIPE--------------------------------

L.major (XP_001686914): CLWLQSCVYNCPQSGTALLRQQGEER---LLSETRHAA----------------------

L.infantum (XP_001469682): CLWLQSCVYNCPQSGTALLRQQGEER---LLSETRHAA----------------------

T.cruzi (XP_819187): MVTLQTRVYDEFRYT-KLLRHLYDRT---ATRHTRHEE----------------------

T.cruzi (XP_812333): MVMLQTRVYDEFRYT-KLLRHLYDRT---ATRHTRHEE----------------------

L.major (XP_001686223): ------------------------------------------------------------

L.infantum (XP_001468509): ------------------------------------------------------------

L.braziliensis (XP_001564414): ------------------------------------------------------------

T.cruzi (XP_817508): SAVLQQRIFSEFFFAAYLIGL-CRGS---LAAVERNRE----------------------

T.cruzi (XP_820998): SAVVQQRIFSEFFFAAYLIGL-CRGS---LAAVERNRE----------------------

T.vaginalis (XP_001319509): LEFLLNNALNSPSYLDIYNKEK------------RRC-----------------------

T.vaginalis (XP_001582897): LEFLLKCCLDSPLYNEVHAKEL------------KRA-----------------------

T.vaginalis (XP_001581503): LEFLLRSCLNSPMYRMIHEKEL------------KRA-----------------------

T.vaginalis (XP_001580012): LESLVIHIMETPMFQECRKDSI------------QRL-----------------------

T.vaginalis (XP_001305124): LESLIIHVMETPMFQTCRKDGI------------QRL-----------------------

N.gruberi (XP_002682879): LYYLQILLFESEDYI-HFINLLYRESQERATKYRRNQAYRAAQILSRFAEGLFSEKRRDA

D.discoideum (XP_640187): LLAYQKMIFQSRDFH-ILEEH---------LKAKRDLNYETA------------------

D.rerio (XP_696355): FLLLQRRVFLSFYFL-HVSADL----QASARQASRGFELFRASIIKNMHF------HQQA

D.melanogaster (AFB77909): FIIFQLRIFKSHYFC-HIITDT----KANNILASRGADIIESLRHKQIAH------RHDH

M.musculus (NP_001032375): FLLLQRRIFLSHYFL-HVSADL----KATALQASRGFALYNAANLKSINF------HRQI

R.norvegicus (NP_001070668): FLLLQRRVFLSHYFL-HVSADL----KATALQASRGFALYNAANIKNINF------HRQT

B.taurus (XP_002694905): FLLLQRRVFLSYYFL-HVQAEL----RATALQASRGFALYNAANLKTIEL------HRRA

C.lupus (XP_546782): FLLLQRRVFLSRYFL-HVSAEL----QATALQASRGFALYNAANFKSIDL------HRKA

A.thaliana (NP_182327): LVSLQSYMFSSQEFD-YVSRYLE----AEQIGA---------------------------

O.sativa (NP_001172359): LVSIQSYIFSSGEFD-YVSRYLE----AEQIGA---------------------------

hPiezo1 (NP_001136336): ------------------------------EE------KSLAQLKRQMERIRAKQEK--H

hPiezo2 (NP_071351): ------------------------------EK------KSMDQLKRQMDRIKARQQK--Y

E.histolytica (XP_649449): ---------------------------------------VFKQIKEIEEEQKEIIF---S

E.histolytica (XP_655549): ---------------------------------------TFRKLEDNIEKERANIF---V

L.major (XP_001686914): ---------------------------------------LQRQLLS------HMAN----

L.infantum (XP_001469682): ---------------------------------------LQRQLLS------HMAN----

T.cruzi (XP_819187): ---------------------------------------LWAWLRGKQEAEQQLAD----

T.cruzi (XP_812333): ---------------------------------------LWAWLRGKQEAEQQLAD----

L.major (XP_001686223): ------------------------------------------------------------

L.infantum (XP_001468509): ------------------------------------------------------------

L.braziliensis (XP_001564414): ------------------------------------------------------------

T.cruzi (XP_817508): ---------------------------------------MLEYFRRKDEELQCQVV----

T.cruzi (XP_820998): ---------------------------------------MLEYFRRKDEELQCQVV----

T.vaginalis (XP_001319509): ---------------------------------------QYR-------KDRQIIIQ---

T.vaginalis (XP_001582897): ---------------------------------------EFR-------RNRAHIIE---

T.vaginalis (XP_001581503): ---------------------------------------EFR-------RNRTKIIE---

T.vaginalis (XP_001580012): ---------------------------------------AYR-------FIRVRQLK---

T.vaginalis (XP_001305124): ---------------------------------------AYR-------FIRTRQLK---

N.gruberi (XP_002682879): NRQALRSFHEVNKDSDFQSFYNRKHMLKEEYEKV---VNAYAKNSEKIERERAILA---L

D.discoideum (XP_640187): -----REFYKIRRNARI---------------------EQLNSIQDKITQRRSRLQH--L

D.rerio (XP_696355): ------------------------------ER------KSIQQLKESMKRIRGKQQK--Y

D.melanogaster (AFB77909): ------------------------------EK------QVLHKIKRKMERIRATQQK--M

M.musculus (NP_001032375): ------------------------------EE------KSLAQLKRQMKRIRAKQEK--Y

R.norvegicus (NP_001070668): ------------------------------EE------RSLAQLKRQMKRIRAKQEK--Y

B.taurus (XP_002694905): ------------------------------EE------KSLAQLKRQMERIRAKQEK--H

C.lupus (XP_546782): ------------------------------EE------KSLAQLKRQMERIRAKQEK--H

A.thaliana (NP_182327): -------------------------IVREQEKKAARKTEQLQQIREAEEKKRQRNLQVEK

O.sativa (NP_001172359): -------------------------MVHEQEKKALKKTEQLQHLRRSEEQKRERNMQVER

hPiezo1 (NP_001136336): RQG----RVDRS-------RPQ-DTLGPKDPGL---------EPGPDSPGGSSPPRRQWW

hPiezo2 (NP_071351): KKG----KERML-------SLT-QEPGEGQDMQKLS---EEDDEREADKQKAKGKKKQWW

E.histolytica (XP_649449): RTRELIDLDRDK-------ILF--ELDKEYNDKIMRM--IRLE-----ELRKL-RT----

E.histolytica (XP_655549): KLKELIDLDRDK-------IMI--ELDKKYNEKVLRM--KRLD-----ELREL-RK----

L.major (XP_001686914): ATEKTVRVD-QE-------VCS--YL----------------------------------

L.infantum (XP_001469682): ATEKVVRVD-RE-------VCS--YL----------------------------------

T.cruzi (XP_819187): ARSRALHAKLEE-------IRS--TL-----------------------------H----

T.cruzi (XP_812333): ARSRALHAKLEE-------IRS--TL-----------------------------H----

L.major (XP_001686223): ------------------------------------------------------------

L.infantum (XP_001468509): ------------------------------------------------------------

L.braziliensis (XP_001564414): ------------------------------------------------------------

T.cruzi (XP_817508): KEEEALKRRLEQ-------LFS--ER----------------------------------

T.cruzi (XP_820998): KEEEALKRRLEQ-------LFS--ER----------------------------------

T.vaginalis (XP_001319509): ---DLF------------------KLDQEYCDEYFNKELDRLKA------GVYS------

T.vaginalis (XP_001582897): ---EMF------------------EIDHKYCDQNFKHTLEVLKS------GIVN------

T.vaginalis (XP_001581503): ---EMI------------------EIDQKFADLYFKHTLKILEE------GVVN------

T.vaginalis (XP_001580012): ---VIS------------------RLNQEVVDSIQHAKIEQIFRIAD--QSSAG------

T.vaginalis (XP_001305124): ---VIN------------------RLNQEIVENLQHKKIDDIFTIAE--STSN-------

N.gruberi (XP_002682879): AMSDLLDLENKESAKIRKKIVS--NLNKEE--QLMKKCLKKAEHKFQKKMREVVST----

D.discoideum (XP_640187): KLK---RINRRK---NRHNHYYNNNPNNNYNNNNNNN-----------NSNSSNSN----

D.rerio (XP_696355): KDG----RITSD-------IEE-QPSDPKE----------------QKKETKLSKSKSWQ

D.melanogaster (AFB77909): LRP----LDKQT-------HFD--------------------EHGYPLPAPTV----RRR

M.musculus (NP_001032375): RQS----QASRG-------QLQ-SK-DPQDPSQ---------EPGPDSPGGSSPPRRQWW

R.norvegicus (NP_001070668): RQS----QASRG-------QLQ-ST-DPQ-------------EPGPDSPGGSSPPRTQWW

B.taurus (XP_002694905): RQG----RANRG-------CLQ-G---SPDPGQ---------EPGPSSPGGSSPPQQQWW

C.lupus (XP_546782): RQS----RAGRS-------RPQ-DTP---DPTQ---------EPGPGSPGGSSLPRRQWW

A.thaliana (NP_182327): MKSEMLNLRVQL---HRMNSDS--NFGV------------------ASPRTEGLRR--RK

O.sativa (NP_001172359): MKSEMYNLQSQL---NRMNSFT--PINN------------------AS-HSEGLRH--RR

hPiezo1 (NP_001136336): RPWL--DHATVIHSGDYFLFESDSEEEE--EAVPE----DPRPS---------AQSAFQ-

hPiezo2 (NP_071351): RPWV--DHASMVRSGDYYLFETDSEEEEEEELKKE----DEEPP---------RRSAFQ-

E.histolytica (XP_649449): -------------------KEGLSDSIENPKVIEPAQFLSTTPD---------SSSFFEK

E.histolytica (XP_655549): -------------------KDRLSDFHDNDF-VEVAETAPKEIT---------TKGFFQN

L.major (XP_001686914): ---------NALRAGDDMVFTCQVCQRRSSEHVEE-----------NGDEHDSDAERVR-

L.infantum (XP_001469682): ---------DALRAGDDVVFTCQVCSRRSPDYVEE-----------DGDEHDSDAERVR-

T.cruzi (XP_819187): ------GAHHAWVPEDSSTQKCSANAVTNSQQLKDEKMMMMMARQRIADDDDDTAARLK-

T.cruzi (XP_812333): ------GAHHAWVPEDSSTQKCSANAVTNSQQLKDEKMMMMMARQRIADDDDDTAARLK-

L.major (XP_001686223): -----------------------------------------VSLSMVSAEET---RRAA-

L.infantum (XP_001468509): -----------------------------------------VSLSMVGAEET---RRAA-

L.braziliensis (XP_001564414): -----------------------------------------APPTMTSDGGA---RRSA-

T.cruzi (XP_817508): -----------------NMCACQD----------EIKIGGLMPTQRVNEKETREEREFS-

T.cruzi (XP_820998): -----------------NMCACQD----------EIEIGALMPPQRVNEKETREEREFS-

T.vaginalis (XP_001319509): ---------------L---NVTNDTTSESPDLIQFEAM---VNR-----QDDIQNNDV--

T.vaginalis (XP_001582897): ---------------L---NYTDGMDVDA-DLIKFSSS-----------RDPIEEN----

T.vaginalis (XP_001581503): ---------------L---NITEDIQNTH-SVED--------------------------

T.vaginalis (XP_001580012): ----------LNRSAS---FLMPQQQQKKPEKVDTRT----EEQ-----KQKDEEEAI--

T.vaginalis (XP_001305124): ----------MSRSVF---NLLPSMST--PNLAEKAKI---EQK-----KQQKE------

N.gruberi (XP_002682879): -------------------LENLQNYKSSTLVVDN-------------------------

D.discoideum (XP_640187): -------------------NNNNDDDSNEPLSLGDNSFVPPKNT---------TNQ---N

D.rerio (XP_696355): YPWV--DHATVLHSGEYYMFESDSGEED--ETFQE----EQKPR---------RQTAFQ-

D.melanogaster (AFB77909): KEIK--LHPHATRAGDYYMFEEMDDKFELDLIHDEIDFLEEENI---------TESEMK-

M.musculus (NP_001032375): RPWL--DHATVIHSGDYFLFESDSEEEE--EALPE----DPRPA---------AQSAFQ-

R.norvegicus (NP_001070668): RPWQ--DHATVIHSGDYFLFESDSEEEE--EALPE----DPRPA---------AQSAFQ-

B.taurus (XP_002694905): RPWL--DHATVIHSGDYFLFESDSEEEE--EAQPE----DPRPS---------SQSAFQ-

C.lupus (XP_546782): RPWL--DHATVIHSGDYFLFESDSEEEE--EALPE----DPRPS---------AQSAFQ-

A.thaliana (NP_182327): SPYLIPD--------------SGAASPEIDGVVHR---KEEQPI--------DEDSQYP-

O.sativa (NP_001172359): NTKLYTDIDTPL-------QDSGIGSPRKEDKTG--------ST--------DSSQSFE-

hPiezo1 (NP_001136336): --------------------------------------------------------LAYQ

hPiezo2 (NP_071351): --------------------------------------------------------FVYQ

E.histolytica (XP_649449): IIKVTKTYVLKGIDIFIH---FLHNRN-LIVRLNIPKDS-------------SEEE----

E.histolytica (XP_655549): LCDTTKEISLNCIDALIH---FLHSRN-LVVRLNLPKNA-------------TDEQ----

L.major (XP_001686914): --------------VSM------------------PSPP------------SMTPVLEPE

L.infantum (XP_001469682): --------------VSM------------------PSPP------------SMTPVLEAE

T.cruzi (XP_819187): --------------REE------------------EEEE--------------EEKEERS

T.cruzi (XP_812333): --------------REE------------------EEE------------------EERS

L.major (XP_001686223): -------------DARP------------------PLSS------------LLARLRGRA

L.infantum (XP_001468509): -------------DVRP------------------PASS------------LLARLHGRA

L.braziliensis (XP_001564414): -------------DGCA------------------LVSL------------LLKGLRSRA

T.cruzi (XP_817508): -------------SGVA------------------PLSP------------MMGSVLK--

T.cruzi (XP_820998): -------------SGVA------------------PHSP------------MMGSVSK--

T.vaginalis (XP_001319509): -----------------------------------PVQ---------------EAEN---

T.vaginalis (XP_001582897): ------------------------------------------------------------

T.vaginalis (XP_001581503): ------------------------------------------------------------

T.vaginalis (XP_001580012): -----------------------------------RKQ---------------AEEEERK

T.vaginalis (XP_001305124): -------------------------------------------------------EEAKK

N.gruberi (XP_002682879): ----------SAKRRPVH---FER------GQLDLPSDPNQE----FLPSNISKEE----

D.discoideum (XP_640187): A--TNSTYSPF-ANSTMHMPPYENNNNNNNNNNNFNNNPLSNSSSTVSSFGVIEKPLEKK

D.rerio (XP_696355): --------------------------------------------------------LAYQ

D.melanogaster (AFB77909): --------------------------------------------------------MQRR

M.musculus (NP_001032375): --------------------------------------------------------MAYQ

R.norvegicus (NP_001070668): --------------------------------------------------------MAYQ

B.taurus (XP_002694905): --------------------------------------------------------MAYQ

C.lupus (XP_546782): --------------------------------------------------------MAYQ

A.thaliana (NP_182327): ---------------------F----------------------------EAH-------

O.sativa (NP_001172359): ---------------------F----------------------------SVEDAQ----

hPiezo1 (NP_001136336): AWVT-NAQAVLR-------------------RR-QQEQEQARQEQ---------------

hPiezo2 (NP_071351): AWIT-DPKTALR-------------------QRHKEKKRSAREERKRRRKGS--------

E.histolytica (XP_649449): --IKKEVNETIRPIIQPDL-INLE------------------------------------

E.histolytica (XP_655549): --IEDSLYTTIKPVSKEPS-ATFTPKNGSTRQTPINRVDVITSSD---MVQSDDDDNVFY

L.major (XP_001686914): E----V-NKVL-------------------------------------------------

L.infantum (XP_001469682): E----V-NEVL-------------------------------------------------

T.cruzi (XP_819187): A----KGSMLT-------------------------------------------------

T.cruzi (XP_812333): A----KGSMLT-------------------------------------------------

L.major (XP_001686223): ESVAHKAVELLR---------------------------------------------GYL

L.infantum (XP_001468509): RWVAHKVVELLR---------------------------------------------DYL

L.braziliensis (XP_001564414): RLAAHKVLDLLG---------------------------------------------GYL

T.cruzi (XP_817508): --VPLSVSAQSD---------------------------------------------TYF

T.cruzi (XP_820998): --VPLSVSAQSD---------------------------------------------TYF

T.vaginalis (XP_001319509): ----------------------------------YNQQQNQENQT---------------

T.vaginalis (XP_001582897): ----------------------------------KEQQKKEEEKP---------------

T.vaginalis (XP_001581503): -----------------------------------IKQTIIDKEP---------------

T.vaginalis (XP_001580012): K------------------------------KEAEEKQRILEEKK---------------

T.vaginalis (XP_001305124): K------------------------------K-----QALLDN-----------------

N.gruberi (XP_002682879): --VQKVMKNILSKIVPHDRIVALGDVLGAPSSKLEQQLNIISKH----------------

D.discoideum (XP_640187): NWVKIYLNKILDWLDPLPDILIEN----YKKQQLQQQQ----------------------

D.rerio (XP_696355): AWVT-SAKDALK-------------------ERQQRQRKQRAEAEKQQNAQATDD-----

D.melanogaster (AFB77909): KTLYDKSKDAPT-------------------GEFPSTSKGISKER--DAATASSS-----

M.musculus (NP_001032375): AWVT-NAQTVLR-------------------QRR----ERARQER---------------

R.norvegicus (NP_001070668): AWVT-NAQTVLR-------------------QRR----EQARRDR---------------

B.taurus (XP_002694905): AWVT-NAQTVLR-------------------QQRQQRAEQLRTGE---------------

C.lupus (XP_546782): AWVT-NAQTVLR-------------------Q---QREEQARQDQ---------------

A.thaliana (NP_182327): ----------------------------------EF------------------------

O.sativa (NP_001172359): KSLTDLMFRTPC-------------------DTPRS------------------------

hPiezo1 (NP_001136336): -----------------A-GQLPTGGGPSQE-----VEPAEGP---EEAAA---GRSHV-

hPiezo2 (NP_071351): ----------------KE-GPVEWED---RE-----DEPI------KKKSD---GPDNI-

E.histolytica (XP_649449): -------------------ST----EEEKD----------EQ-------MNNT-------

E.histolytica (XP_655549): SVKSSPENL-------SINSSNSSSEEESEV-----SEEESEVSEEESEVSEEESEVSEE

L.major (XP_001686914): ------------DDAVEL-PR----------------PPLTFPAAAGRNNV---------

L.infantum (XP_001469682): ------------DDAGEL-PR----------------PLLTFPAADGRSNA---------

T.cruzi (XP_819187): ------------DDKVKV-SK----------------KK----SEKGGTC----------

T.cruzi (XP_812333): ------------DDKVKG-SK----------------KK----SEKDGTC----------

L.major (XP_001686223): WRHTILLPAEVENSATEC-PAGPRADVVH---------PQRQPRRQ--------------

L.infantum (XP_001468509): RRHTVPLPTEVENSATEC-PAGPRTDAAH---------PQRQRRRQ--------------

L.braziliensis (XP_001564414): QRHTAPLPAEVDDSAGEC-PTGPRADAPH---------AQRQRCRQ--------------

T.cruzi (XP_817508): SVLDSPKQLTKAGAAVDC-PATTFDGRLSGVCSEEHVPPAREDDRKGGICS---------

T.cruzi (XP_820998): SVLDSPKQLTKAGAAVDC-PAATFDGRLSGVCSEEHVPPAREDDRKGGICS---------

T.vaginalis (XP_001319509): --------N-------E---------Q---------QENAGNDPNNAVVV--------PT

T.vaginalis (XP_001582897): --------N-------E--------GE---------ENPVGPDGNPDQPP--------MS

T.vaginalis (XP_001581503): --------S-------L--------PK---------YKLITNDGNPDAPP--------IS

T.vaginalis (XP_001580012): --------A-------KA-----AAEG---------KEYIPEEEEEEEEP--------PK

T.vaginalis (XP_001305124): -------------------------SE---------EDY----EEDESEK--------KK

N.gruberi (XP_002682879): -----ARND-------SVN------EALSKV--------IQQV---APEIAQEGSQENKK

D.discoideum (XP_640187): -------KL-------EMNQSLLFGDQ---L-----QQEQQQQQEQQQQLNPQQQQSQSS

D.rerio (XP_696355): --------A-------EE-GETVFDGENSQH-----AEEMQEEGEDAASQA---TGSDM-

D.melanogaster (AFB77909): --------A-------SP-APTRDVGDLPVI-----PPPSTGLGREQTSKETSDSKSKME

M.musculus (NP_001032375): -----------------A-EQLASGGDLNPD-----VEPVDVP---EDEMA---GRSHM-

R.norvegicus (NP_001070668): -----------------A-EQLASGGDLSPE-----VELVDVP---ENEMA---GHSHV-

B.taurus (XP_002694905): -----------------S-GPLCLGGDPSQE-----AGPAEGL---EDEVT---GRSHV-

C.lupus (XP_546782): -----------------T-GQLPTGGGPGQE-----AEPADGP---DEVVA---GRSHV-

A.thaliana (NP_182327): PVSTTPEAL-----D---SPEYSFGASPCEI---------TEVQQDLDVMS--MERERKQ

O.sativa (NP_001172359): PIRGTSEEF-----KVTDNARNSLG-STSEI---------TEVEENEGKVNHNLLKLQYG

hPiezo1 (NP_001136336): -----V-----QRVLSTAQFLWMLGQALVDELTRWLQEFTRHHGTMSDVLRAERYLLTQE

hPiezo2 (NP_071351): -----I-----KRIFNILKFTWVLFLATVDSFTTWLNSISREHIDISTVLRIERCMLTRE

E.histolytica (XP_649449): ----------------------EI------------------------------------

E.histolytica (XP_655549): -----------EK---NPPP-SVK------------------------------------

L.major (XP_001686914): ------------------PT----------------------------------------

L.infantum (XP_001469682): ------------------PT----------------------------------------

T.cruzi (XP_819187): -------------------A----------------------------------------

T.cruzi (XP_812333): -------------------A----------------------------------------

L.major (XP_001686223): ------------------------------------------------------------

L.infantum (XP_001468509): ------------------------------------------------------------

L.braziliensis (XP_001564414): ------------------------------------------------------------

T.cruzi (XP_817508): ------------------------------------------------------------

T.cruzi (XP_820998): ------------------------------------------------------------

T.vaginalis (XP_001319509): ---------STRVFYI---SLLKWIFGLIWGVVVRLVDR-------LTVM----------

T.vaginalis (XP_001582897): ---------ATKQFYM---TILKFVLMCLKTAFLWVIDK-------LIVL----------

T.vaginalis (XP_001581503): ---------PVKLLYH---SIIGWVLRLLRWFFLWLVDS-------LIEL----------

T.vaginalis (XP_001580012): ---------TVKD------RILGFWDIFMENIVWPLCSH-------IIIF----------

T.vaginalis (XP_001305124): ---------PLKE------ILYDLIQDFKDKVLKPILRK-------LLLL----------

N.gruberi (XP_002682879): -----------EE---STPK-LPLLFRAKSLLAVV-FANVGHYAYMLTDLIVNRIQIFIG

D.discoideum (XP_640187): -----------KE---LPPI-LEQFEHNDDDFEISLNQAPGDYR----------NSIFID

D.rerio (XP_696355): -----V-----QRILDILKFLWVLFLAMVDGFTMWLNLLTKQYVDTSMVLSEERYLFIHN

D.melanogaster (AFB77909): VDSGEVTAKDSDEDFDTNPII-RLLEGFLVTLTIRLNRFSRNYRFVNRILAGEKKTLKES

M.musculus (NP_001032375): -----M-----QRVLSTMQFLWVLGQATVDGLTRWLRAFTKHHRTMSDVLCAERYLLTQE

R.norvegicus (NP_001070668): -----M-----QRVLSTMQFLWVLGQATVDGLTRWLRTFTKDHRTMSDVLCAERYLLTQE

B.taurus (XP_002694905): -----M-----QRVLSAVQFLWVLGQALVDGLTRWLHDFTRHHRAISDVLRAERYLLTQE

C.lupus (XP_546782): -----M-----QRVLSTVQFLWVLGQALVDGLTDWLHTFTRHHRAMSDVLRAERYLYTQA

A.thaliana (NP_182327): --KSEG-----KE---N-PL----------------------------------------

O.sativa (NP_001172359): --RGAV-----KE---N-PL----------------------------------------

hPiezo1 (NP_001136336): LLQGG-----EVHRGVLDQLYTSQAEA-TLPGPTEAPNAPSTVSSG--------------

hPiezo2 (NP_071351): IKKGN-----VPTRESIHMYYQNHIMNLSRESGLDTIDEHPGAASGAQTAHRMDSLDSHD

E.histolytica (XP_649449): ----------------------------------------LLTKNETSE-----------

E.histolytica (XP_655549): ----------------------------------------LVPKNEVQH-----------

L.major (XP_001686914): ------------------------------------------PSYD--------------

L.infantum (XP_001469682): ------------------------------------------LSSD--------------

T.cruzi (XP_819187): ------------------------------------------HKFK--------------

T.cruzi (XP_812333): ------------------------------------------HKFK--------------

L.major (XP_001686223): ------------------------------------------------------------

L.infantum (XP_001468509): ------------------------------------------------------------

L.braziliensis (XP_001564414): ------------------------------------------------------------

T.cruzi (XP_817508): ------------------------------------------------------------

T.cruzi (XP_820998): ------------------------------------------------------------

T.vaginalis (XP_001319509): --------------------ITYFT-----------------------------------

T.vaginalis (XP_001582897): --------------------VSNFT-----------------------------------

T.vaginalis (XP_001581503): --------------------GSNFT-----------------------------------

T.vaginalis (XP_001580012): --------------------YAKTV-----------------------------------

T.vaginalis (XP_001305124): --------------------VINIC-----------------------------------

N.gruberi (XP_002682879): IAWMDELEIKKKYRVNLDEIIADAINE-Y----IEEKEEEWKKKNETTT-----------

D.discoideum (XP_640187): SEQMR-----------------NAMQQ-M----EQRRQQRL------QQ-----------

D.rerio (XP_696355): VSQRA-----S-RENMDDQISHDSEDL-TLETCLDETDTDNICDTN--------------

D.melanogaster (AFB77909): SSL---------NRLGL---SSAAAMFHFLKSNLESDESEPPASSSTPR--RV-------

M.musculus (NP_001032375): LLRVG-----EVRRGVLDQLYVGEDEA-TLSGPMETRDGPSTASSG--------------

R.norvegicus (NP_001070668): LLRGG-----EVHRGVLDQLYVSEDEI-ALSGPVENRDGPSTASSG--------------

B.taurus (XP_002694905): LLRGG-----EVRRDVLDQLYANEAEA-A-------RDALSTASSG--------------

C.lupus (XP_546782): LVEGR-----EVHRSLLDQLYTGEAEA-APAVPLGVRDAPSTASSG--------------

A.thaliana (NP_182327): ------------------------------------------------------------

O.sativa (NP_001172359): ------------------------------------------------------------

hPiezo1 (NP_001136336): ---------------LGAEEPLSSMT----DDMG-------------------SPLSTGY

hPiezo2 (NP_071351): SISSEPTQCTMLYSRQGTTETIEEVEAEQEEEAGSTAPEPRE-----AK----EYEATGY

E.histolytica (XP_649449): --------T------------------------------------------IS-------

E.histolytica (XP_655549): --------Q------LKSMSDIIEIEQKE---------------ETSQKDSLSNFENTNV

L.major (XP_001686914): ----------------QAQPPLLRCWQQR----------------------CVLWLR---

L.infantum (XP_001469682): ----------------QAQPPLSRCWRQR----------------------CARWLR---

T.cruzi (XP_819187): ----------------TGLQRIIHVSATK----------------------LANW-----

T.cruzi (XP_812333): ----------------TGIQRIIHVSATK----------------------LANW-----

L.major (XP_001686223): -----------------PVEKVAEHTQPA----------------------FASATS---

L.infantum (XP_001468509): -----------------PMEKAVECTQPA----------------------FASAAS---

L.braziliensis (XP_001564414): -----------------SMERMTKHSQAG----------------------FTSAAL---

T.cruzi (XP_817508): -----------------KISNAIECWSEQ----------------------LVQYFS---

T.cruzi (XP_820998): -----------------KISNAIECWSEQ----------------------LVQYFT---

T.vaginalis (XP_001319509): DINLEP----------------------------------------------------RV

T.vaginalis (XP_001582897): DINLEP----------------------------------------------------GV

T.vaginalis (XP_001581503): DINLEP----------------------------------------------------GV

T.vaginalis (XP_001580012): NLSCEA----------------------------------------------------GV

T.vaginalis (XP_001305124): PINSEA----------------------------------------------------GI

N.gruberi (XP_002682879): --------T------T---ETLVELEQQPEDEIKLTED-EKEKEEKEDQADIPNLKSKEF

D.discoideum (XP_640187): --------S-------IDASQLLQQQQQQQQASSSNTNTNSNNNNYNNNNNNNNNNNNNN

D.rerio (XP_696355): ---------------VVESETLGGFA-----------------------------KSRDF

D.melanogaster (AFB77909): ---------------------------------VIAPPNATE-----HS----DPTSTTL

M.musculus (NP_001032375): ---------------LGAEEPLSSMT----DDTS-------------------SPLSTGY

R.norvegicus (NP_001070668): ---------------LGAEEPLSSMT----DDTG-------------------SPLSTGY

B.taurus (XP_002694905): ---------------LGVEEPLSSVT----EDTS-------------------SPLSTGY

C.lupus (XP_546782): ---------------LGAEEPLSTAT----EDTG-------------------SPLSTGY

A.thaliana (NP_182327): ----------------ISAVQLIGDGVSQVQFIGNQ-----------AVNNLVNFLNI--

O.sativa (NP_001172359): ----------------KSAVQLIGDGVSQVQSFGNQ-----------AVTNIVSFLNI--

hPiezo1 (NP_001136336): HTRSGSEEA------------V-T----DPGER---------------------------

hPiezo2 (NP_071351): DVGAMGAEEASLTPEEELTQFSTLDGDVEAPPSYSKAVSFEHLSFGSQDDSAGKNRMAVS

E.histolytica (XP_649449): ------------------------------------------------------------

E.histolytica (XP_655549): KSNTTTPEK-----------------------K-----------------------VT--

L.major (XP_001686914): --------------------WI-----CDKLAA---------------------------

L.infantum (XP_001469682): --------------------RI-----CDKLAA---------------------------

T.cruzi (XP_819187): ------------------------------LAV---------------------------

T.cruzi (XP_812333): ------------------------------LAV---------------------------

L.major (XP_001686223): --------------------LTAAEGITPPPGI---------------------------

L.infantum (XP_001468509): --------------------LTTAEGITSPPGI---------------------------

L.braziliensis (XP_001564414): --------------------STDAKGVTALLGT---------------------------

T.cruzi (XP_817508): --------------------FRTYRG----------------------------------

T.cruzi (XP_820998): --------------------FRTYRG----------------------------------

T.vaginalis (XP_001319509): HTGFLNKLKEMM---ETMVSVYKSDHVLQ-----------------------------IP

T.vaginalis (XP_001582897): HTQFLERLRDMM---NEMVETFVNTNVLT-----------------------------IP

T.vaginalis (XP_001581503): HTDFLLKVRDMM---TEMVDKFSNSNVLA-----------------------------IP

T.vaginalis (XP_001580012): NVLTFNSVNAIL---RRIAAALTAGGTYQ-----------------------------LT

T.vaginalis (XP_001305124): NVLTLNSIHSIL---RRYASVMRISGSFT-----------------------------LS

N.gruberi (XP_002682879): TTQELN------------------------------------------------------

D.discoideum (XP_640187): NNNNNNNNN-----------------------N-----------------------NN--

D.rerio (XP_696355): SSTTLSSEL------------T-LELDCEP------------------------------

D.melanogaster (AFB77909): NTNTTTTPL---SPPEPLQPL-------QPNTTSTPQQQHQHIR-------AAEEIIELP

M.musculus (NP_001032375): NTRSGSEEI------------VTDAGDLQAGTS---------------------------

R.norvegicus (NP_001070668): NTRSGSEEI------------ITDTGGLQAGTS---------------------------

B.taurus (XP_002694905): NTRSSSEE-------------VVT----EPGAS---------------------------

C.lupus (XP_546782): HTRSSSSEE------------VAT----EPGAS---------------------------

A.thaliana (NP_182327): ------------SPENS-------------------------------------------

O.sativa (NP_001172359): ------------DPEEP-------------------------------------------

hPiezo1 (NP_001136336): -------EAGASL----YQG--LMRTASELLLDRRLRIPELEEA------ELFAEGQGRA

hPiezo2 (NP_071351): PDDSRTDKLGSSIL--PPLT--HELTASELLLKKMFHDDELEES------EKFYVGQPRF

E.histolytica (XP_649449): -------------------K--KENKFE--------LLPIKTYS------IEPYKYKSRL

E.histolytica (XP_655549): -MKTSRIYSNKNQIPIHKPI--PQRNYE--------FIQI-HER------NEPYKYKGRI

L.major (XP_001686914): ------------CTYHPSEY--RLSAVVADSSTSTLW-LL--SQ----------------

L.infantum (XP_001469682): ------------CTYHPSEY--RLSAVAADSSTSTLW-LL--SQ----------------

T.cruzi (XP_819187): ------------HTYEPHRH--TAAH-YRGLFSRLFW-V---------------------

T.cruzi (XP_812333): ------------HTYEPHRH--TAAH-YRGLSSRLFW-V---------------------

L.major (XP_001686223): ------------RVGAPSST--VSRR--EVVHLPSLWRLL--EL----------------

L.infantum (XP_001468509): ------------CLGAPSSA--VSRR--EVVHLPSLWRLL--EL----------------

L.braziliensis (XP_001564414): ------------LVGAPPSS--TSHP--ETIHVRSLWKLL--EL----------------

T.cruzi (XP_817508): --------------KAPPRT--YSQG------YRVLF-AF--TQ----------------

T.cruzi (XP_820998): --------------KAPPRT--YSQG------YRVLF-AF--TQ----------------

T.vaginalis (XP_001319509): DQYTDF------VKTIPLSFLRHFQIF-EPLE-----LKKVTPE--------------ER

T.vaginalis (XP_001582897): QAYSGF------VQQIPMSYLYHFQIF-HKLQ-----IKTIDEK--------------NR

T.vaginalis (XP_001581503): QNYVDF------SKQLPLSYLNHFQIF-HSLD-----LLSITEK--------------TR

T.vaginalis (XP_001580012): PIEQNF------LKKLPPSFYMQLGGIGEILQ-----FPEIKRE--------------QY

T.vaginalis (XP_001305124): PAEQNF------MKKLPPSIFYQISSLGDLCG-----FPELKDE--------------RL

N.gruberi (XP_002682879): ----DLLQLKLEQSKVHPDM--KKKPFN--------WVPIVLKL------K---------

D.discoideum (XP_640187): -NNNNNNNNNNNEIPKPNQT--SSSTIT--------LEPIEDEE------EFLYKEKTQY

D.rerio (XP_696355): ----------FYSQQTHRPH--RSRTASELLSERPFRVDELIQS------REFYSSQNRL

D.melanogaster (AFB77909): VDTVDGVAHRKQSINSSPPA----KGAGEF---------NLEEE------NFAQRDHHII

M.musculus (NP_001032375): -------LHGSQELLANART--RMRTASELLLDRRLHIPELEEA------ERFEAQQGRT

R.norvegicus (NP_001070668): -------LHGSQELLANART--RMRTASELLLDRRLRIPELEEA------EQFEAQQGRT

B.taurus (XP_002694905): -------LRSSGELPAGGRA--RMRTASELLVDRRVHIPELEEA------EQFAAGRGRA

C.lupus (XP_546782): -------LHGSRELPAGTPT--RMRTASELLLSRHLRIQELEEA------ELFEAGQGRA

A.thaliana (NP_182327): ----DTNEQ--------SSV--DDEVYDEMESQKRKHTPFE-RSTSLQSDR-SSDGTSFQ

O.sativa (NP_001172359): ----HSSDH---------PA--EDDIYDMVESQRETHDGQLLRTHSVTSGNGTKSSANMP

hPiezo1 (NP_001136336): L-RLLRAVYQCVAAHS-ELLCYFIIILNHMVTASAGSLVLPVLVFL-WAMLSIPRPSKRF

hPiezo2 (NP_071351): L-LLFYAMYNTLVARS-EMVCYFVIILNHMVSASMITLLLPILIFL-WAMLSVPRPSRRF

E.histolytica (XP_649449): Y-MLLWGIWFYCSEQT-EVFIQILFIINHLFNQNILSSFYPIIGFSIIMMCKRPNPSKVI

E.histolytica (XP_655549): Y-MLFWGLCFYFGQQT-EVLVQLILVLDELINQNILSAVFPIIGFAVIALSIRPYPTKFF

L.major (XP_001686914): --YALRAAAQVVLRHT-SFALLGCSVVNSLLTGCLWELIGLCYVVQ-VALAYHPHPPRVV

L.infantum (XP_001469682): --YALRAAVQVVLRHT-PFALLGCSVVNSLLTGCLWELIGLCYVVQ-VALAYHPHPPRLV

T.cruzi (XP_819187): -------TIRALERHT-VMVVLLATMINFMLSQCVWELLPFCFMLV-VAIAYHPFPPRPI

T.cruzi (XP_812333): -------TIRALERHT-VMVVLLATMINFMLSQCVWELLPFCFMLV-VAIAYHPFPPRPI

L.major (XP_001686223): ----------YAVQYW-SSLCALLMLSQFAVCGTVVNLAPSFVSIV-YALLQRPWPPLWY

L.infantum (XP_001468509): ----------YAVQYW-SSLCALLMLSQFAVCGTVVNLAPSFSSIV-YALLQRPWPPLWY

L.braziliensis (XP_001564414): ----------CAVQHW-PYLCALLMLVQFTVSGTVVNLVPSLASIV-YALLQRPWPPLWY

T.cruzi (XP_817508): ----------FLLRKY-AYVCYFLFMLNYALSGTIVDMVLSLSAAL-YGMLILPWSGRIY

T.cruzi (XP_820998): ----------FLLRKY-AYVCYFLFMLNYALSGTIVDMVLSLSAAL-YGMLILPWSGKIY

T.vaginalis (XP_001319509): F-SVFYYYLKVISRQIIPALLLVMAIFYPMEERSIFGILF--------------------

T.vaginalis (XP_001582897): F-PLFYHYVKLCSRQFIPALLLAMCFYYPMEERSIFALFM--------------------

T.vaginalis (XP_001581503): F-PLLYHYLKVCSRQAIPALLLALCFYYPLVERSVFALIL--------------------

T.vaginalis (XP_001580012): R-ELLLRYFGYTIRKLSLPCLVLMSVIYTYVKPYLLSFLF--------------------

T.vaginalis (XP_001305124): F-EHSMRYLGLVVRNLSLPLLLLICVIHLFIRENIWSFIN--------------------

N.gruberi (XP_002682879): --QLLVALTKLFFSNT-DVICYFAMLVNLLYAPTYFNTAFTFFSLT-YAAMQYPYPHRYF

D.discoideum (XP_640187): FKRIIRGFSRIARDES-KWLVFMACIANGVFYNSIISLVYLLAVFLYGRLFESPRPSKNF

D.rerio (XP_696355): L-KLLFALYNVLAAHS-ELVCYFIIVLNNLVTASVISLVLPVLVFL-WAMLSVPRPSKRF

D.melanogaster (AFB77909): V-EVLISSWYALLANT-DLICYIVVFINQVVNASLISLPLPIMVFL-WGTLSLPRPTKTF

M.musculus (NP_001032375): L-RLLRAGYQCVAAHS-ELLCYFIIILNHMVTASAASLVLPVLVFL-WAMLTIPRPSKRF

R.norvegicus (NP_001070668): L-RLLRAMYQCVAAHS-ELLCYFIIILNHMVTASAASLVLPVLVFL-WAMLTIPRPSKRF

B.taurus (XP_002694905): L-RLLEALYQCVAAHS-ELLCYFVIVLNHMVTASATSLVLPVLVFL-WAMLSIPRPSKRF

C.lupus (XP_546782): L-RLLQAAYQCVAAHS-ELLCYFIIILNHMVTASATSLVLPVLVFL-WAMLSIPRPSKRF

A.thaliana (NP_182327): IGRIFRHIWSRMQSNN-DIVCYCCFIIAFLWNFSLLSMVYLAALFL-YALCVHTGPTHIF

O.sativa (NP_001172359): I----------------------------GVIFRLLSMVYLGALFL-YALCVNYGPSYLF

hPiezo1 (NP_001136336): WMTAIVFTEIAVVVKYLFQFGFFPWNSHVVL-----------------------------

hPiezo2 (NP_071351): WMMAIVYTEVAIVVKYFFQFGFFPWNKNVEV-----------------------------

E.histolytica (XP_649449): WKIILWYCFILVFIRMLLELPGFCLTSTRYENY-DA-----LSFTTDAIQ----------

E.histolytica (XP_655549): WNFLSLYNVCLILIRLFFQLPGFCLTPNEYDQQHDV-----ISYTTHIFN----------

L.major (XP_001686914): YKALGVYLGTGMLLKEAASMWTFFGNANPSLITA-------LSWIL-L-PLKG-------

L.infantum (XP_001469682): YKALGVYVGAGVLLKEAASMWTIFGNTNSSLITA-------LSWIL-L-PLKG-------

T.cruzi (XP_819187): FVFFSFYVALGILLKALIDVVLAQVSYSPGLLKI-------LGWTI-L-HVSE-------

T.cruzi (XP_812333): FVFFSFYVALGILLKALIDVVLAQVSYSPRLLKI-------LGWTI-L-HVSE-------

L.major (XP_001686223): WRVQLLFTALAFLAKGAARVYLLTMGPSVSLTTC--------RWLS-LLLLRVGVGAPSA

L.infantum (XP_001468509): WRLQMLFSVLALLAKGTARVYLLAMGPSVSLTTC--------RWLS-VLLLRVDVGAPSA

L.braziliensis (XP_001564414): WRLQMLFSSFVFLVKSGARVYLLTMGPSVSLTTC--------RWLS-LLVLRVDVAASSA

T.cruzi (XP_817508): WRNALIYNVFSILVKCVIQLIV--------------------KWFA-MNHMALKIVSACV

T.cruzi (XP_820998): WRNALIYNVFSILVKCVIQLIV--------------------KWFA-MNQMALKIVSACV

T.vaginalis (XP_001319509): -----FFLSLTTLS--------FNI-----------------------------------

T.vaginalis (XP_001582897): -----FFLALICVA--------FNV-----------------------------------

T.vaginalis (XP_001581503): -----FFLALFCVS--------FNI-----------------------------------

T.vaginalis (XP_001580012): --------TIYIFA--------LFIGTNIYA-----------------------------

T.vaginalis (XP_001305124): --------LVYCFV--------FFAGTNLYA-----------------------------

N.gruberi (XP_002682879): WELMLILAQLLISVQYILYLVQSGLPDNLK------------------------------

D.discoideum (XP_640187): WRFMIGYSSLIICLKYVFQIPKNYYNCNEN--YHNSNNGTILMTSTSVYNTNNNINNNNQ

D.rerio (XP_696355): WMTAIIYTEVMVVVKYLFQFGFFPWNTEYEL-----------------------------

D.melanogaster (AFB77909): WVTLIAYTQAIVLIKCIFQFKLI-WSNYH-------------------------------

M.musculus (NP_001032375): WMTAIVFTEVMVVTKYLFQFGFFPWNSYVVL-----------------------------

R.norvegicus (NP_001070668): WMTAIVFTEVMVVTKYLFQFGFFPWNSYIVL-----------------------------

B.taurus (XP_002694905): WMTAIIFTEVTVVAKYLFQFGFFPWNSHAVL-----------------------------

C.lupus (XP_546782): WMTAIIFTEVSVVTKYLFQFGFFPWNTHTVL-----------------------------

A.thaliana (NP_182327): WVIMLMYTEIYILLQYLYQIIIQHCGLSIDA-----------------------------

O.sativa (NP_001172359): WVIVLIYTELNILSQYIYQIVIQHCGLNIHI-----------------------------

hPiezo1 (NP_001136336): ---------R-------RYENKPYFPPRILGLEKTDGYI----KYDLVQLMALFFHRSQL

hPiezo2 (NP_071351): ------------------NKDKPYHPPNIIGVEKKEGYV----LYDLIQLLALFFHRSIL

E.histolytica (XP_649449): ------SKTIHCDSKPLTYNH--MSPIYLIGIYPVENYITSSFICDIICLFSVIIHISCM

E.histolytica (XP_655549): ------EKTMQCDATPLTKNH--MSPVYLIGIYPVHRYIASRFIWDIFCLFAILIHLSCM

L.major (XP_001686914): -------QGRSSR----NSNQMWANVPYSRGAL-RYD----MLWMDLVTIGVIVLYDRVC

L.infantum (XP_001469682): -------QDRSSS----NSNQVWANVPYSRGPL-RYG----TLWMDLVTIGVIVLHDRVC

T.cruzi (XP_819187): -------G------------------AYWKQRI-GYS----YVMMDFVVFATLLLHQDIC

T.cruzi (XP_812333): -------G------------------AYWKQRI-GYS----YVMMDFVVFATLLLHQDIC

L.major (XP_001686223): PVAS---L--SSP----SSSTM-------FGSE-SHR----DMWVDLALSGCVV--TAVA

L.infantum (XP_001468509): LVAS---L--PSP----SPSAM-------FGSE-SHR----DMWVDLVLSGCVV--TAVA

L.braziliensis (XP_001564414): PAAS---L--LSP----LSSTM-------FGSE-FHR----YMWVDLALSGCVV--AAVA

T.cruzi (XP_817508): LDIG---LDRTEP----SFSAM-------------NL----DIIMDFVLFVSIIIHRQFC

T.cruzi (XP_820998): LDIG---LDRTEP----SFSAM-------------NL----DIIMDFVLFVSIIIHRQFC

T.vaginalis (XP_001319509): -----------------------------------QSYL----P-FVILSIVYMLLRCIL

T.vaginalis (XP_001582897): -----------------------------------ETYI----P-FVITSGIYMFMRCLL

T.vaginalis (XP_001581503): -----------------------------------ESYI----P-FVITSGFYMFLRCIL

T.vaginalis (XP_001580012): ------------------T-------P--------LVYR----I-FYIALIIMLLLRAVC

T.vaginalis (XP_001305124): ------------------K-------P--------NILR----L-FYLFIMLMMLVRAIC

N.gruberi (XP_002682879): -----------------STES--DPAIFLLGWQPSTALLADIV-MYLLIMLAIFFHREIT

D.discoideum (XP_640187): LNNNENYEWWQCPNTLLSEQNLLLTLPYVFGLYIIDGHFISGAFWDLAILLCCLWHRHVY

D.rerio (XP_696355): ---------K-------LNEDKPFFPPRILGLEKTDNYI----RYDLLQLLVLFFHRSLL

D.melanogaster (AFB77909): -----------------QLPNQPLTPAKIFGVENKAHYA----IYDLILLLVLFLHRYLL

M.musculus (NP_001032375): ---------R-------RYENKPYFPPRILGLEKTDSYI----KYDLVQLMALFFHRSQL

R.norvegicus (NP_001070668): ---------R-------RYENKPYFPPRILGLEKTDSYI----KYDLVQLMALFFHRSQL

B.taurus (XP_002694905): ---------R-------RYENKPYFPPRILGLEKSDSYV----KYDLLQLMALFFHRAQL

C.lupus (XP_546782): ---------R-------RYENKPYFPPRILGVEKTDSYI----KYDLVQLMALFFHRSQL

A.thaliana (NP_182327): --------------PLLHEL---GFPT----QRIKSSFVVSSLP-LFLIYIFTLIQSSIT

O.sativa (NP_001172359): --------------PLLQRL---GFPD----DKIKASFVVSILP-LFLVYISTLLQSSIT

hPiezo1 (NP_001136336): LCYGLWDHEEDSPSKEHDKSGEEEQGA---------------------------------

hPiezo2 (NP_071351): KCHGLWDEDDMTESGMAREESD--------D------ELSLGHGRR--------------

E.histolytica (XP_649449): KSRGYWKQQYLL----RRQWSNIIIEN-----YRRQLVNKIYPGTY-------GELLFIV

E.histolytica (XP_655549): RSRGYWKQQYLL----RRQWSNLIIEN-----YRRQLVNKIKPRTF-------GELKFVV

L.major (XP_001686914): IIHGVHVERDQQQS----MERDRSDVM---------------------------------

L.infantum (XP_001469682): IIHGVHVERDQQQS----MERDRSDVT---------------------------------

T.cruzi (XP_819187): LANGVYSRKDILRS----REGEQAAGNTK--------------GTN--------N-----

T.cruzi (XP_812333): LANGVYSRKDILRS----REGEQAAGNTK--------------GTN--------N-----

L.major (XP_001686223): IQWAVVYP----------------------------------------------------

L.infantum (XP_001468509): IQWAVVYP----------------------------------------------------

L.braziliensis (XP_001564414): IQWSIVYP----------------------------------------------------

T.cruzi (XP_817508): FDYGVFANEHEEPFRPHRMETEKGEVHRKKKHHRNE--ES-TAGRR--------E-----

T.cruzi (XP_820998): FDYGVFAKEHEEPFIPHRMETEKGEVHRKEKHHRNE--ES-TAGRR--------E-----

T.vaginalis (XP_001319509): GCYGVKDIISSFL---------------------------VEDKYRKIQI----------

T.vaginalis (XP_001582897): RSYALDPLVETFIG-------------------------SLEENHRKLRV----------

T.vaginalis (XP_001581503): RSYGMKPLVQTFIN-------------------------SLEETHRKLKV----------

T.vaginalis (XP_001580012): TVDLISDQMDKYLN-------------------------SVSAAQSRVSY----------

T.vaginalis (XP_001305124): TVEVVANVINNYMD-------------------------SVAAGTASVNV----------

N.gruberi (XP_002682879): RNRGEWKTNSRK----KRLSAAALASANQQENNTTQQDNPIITTTN--------------

D.discoideum (XP_640187): RSKGLWNFQEKDFYVDQKQQSPLNLFNLDQQHFDQQQIDQIQNQQD-------LNN----

D.rerio (XP_696355): QRYGLWDQEGPLEEKSDLSPAENSKNE--ND------EKKFQHSDE--------------

D.melanogaster (AFB77909): KSQGLWKSGYKDTDNQFTKPTASIDE-------------------R--------------

M.musculus (NP_001032375): LCYGLWDHEEDRYPKDHCRSSVKDREAKEEP------EAKLESQSE--------------

R.norvegicus (NP_001070668): LCYGLWDHEEDGVPKDHCRSSEKDQEAEEES------EAKLESQPE--------------

B.taurus (XP_002694905): LCYGLWDHEDPPLSKEHDRGSAKEKGAEEEP--------ALPAPQE--------------

C.lupus (XP_546782): LCYGLWDHEEDPLSKEHDRGSEKKGTEEEQA--------PLEPQTE--------------

A.thaliana (NP_182327): VKDGDWVPSADFTSRRNARGSQK-DLT--RIRLSQRILDVFKKLRDSAKLVIRSIYRYWI

O.sativa (NP_001172359): AKDGEWVPVTEFSFLSARNNVEEKQRM--PYNWRDRLKNIHLPVMNLIRMIGRGISRYWL

hPiezo1 (NP_001136336): -----EEGPG---V------PA----ATTE-----DHIQVEARVGPTDGTPEPQVEL---

hPiezo2 (NP_071351): DSSDSLKS-----INLA-ASVESVHVTFPE---QQTAVRRK-RSGSS---S---------

E.histolytica (XP_649449): EAITNFTS--DSSDYLS-YNKHDLFVVDKIQENGMLFVKKGNKQGLIHFENVRLFEQQSD

E.histolytica (XP_655549): EALVDFEAPEGEEDFLS-YKKHDLFVVENMQVNGDLFVSKGGKMGLINCENVTIYVQNGK

L.major (XP_001686914): ----VMLEGP---------------------------AS---------------------

L.infantum (XP_001469682): ----AMLEGP---------------------------AS---------------------

T.cruzi (XP_819187): ----DPLPPP---------------------------SP---------------------

T.cruzi (XP_812333): ----DPLPLP---------------------------SP---------------------

L.major (XP_001686223): --------------------DHCVFW-----------N----------------------

L.infantum (XP_001468509): --------------------DHCVFG-----------N----------------------

L.braziliensis (XP_001564414): --------------------DHCVLW-----------R----------------------

T.cruzi (XP_817508): ----LPEEDMDRID------VQCVVV-----------VA---------------------

T.cruzi (XP_820998): ----LPEEDMDRID------VQCVVG-----------VA---------------------

T.vaginalis (XP_001319509): SKVLGLDLNKSQKAYLYIVFILS-------------------------------------

T.vaginalis (XP_001582897): SVCFGLDLNVGEKAYLLIVFCLA-------------------------------------

T.vaginalis (XP_001581503): SVCFGLNLSVGEKIYLLIVLCLS-------------------------------------

T.vaginalis (XP_001580012): PSLFGFNSADSSTIEILMFLVGTWYVVDNL---------------RNA------------

T.vaginalis (XP_001305124): IALFGANPNTDSNVEIFVFLIANWYIVDKL---------------RNA------------

N.gruberi (XP_002682879): ----ATIEPRDDQMILP----------SN----SQILTNTTTTTGRNRANT---------

D.discoideum (XP_640187): SPI-SLNSSNNNNNNNN-N--------NNNNNNNNNNNNNNNNNNNNNNNDQTLIDINNN

D.rerio (XP_696355): EDSQAQPEPNTEVLSLT-PSAE----TPTD-----AEIQTDPEPNSHTNNTKTSDTV---

D.melanogaster (AFB77909): DDSDNLSQPDSRQL----------------------------------------------

M.musculus (NP_001032375): TGTGHPKEPV---L------AG----T-PR-----DHIQGKGSIRSKDVIQDPPEDL---

R.norvegicus (NP_001070668): TGTGHPEEPV---L------TG----T-PK-----DHIQGKGSVRSKDEIQDPPEDL---

B.taurus (XP_002694905): EPGGAA---------------R----PPAE-----DNVQAEAKGG----------PL---

C.lupus (XP_546782): EGTGPQGEPV---V------AG----ALTQ-----DHIQAE----AGDGPPEPPVEL---

A.thaliana (NP_182327): SLTRGAESPP-------------YFVQVTMDVHM--WPEDGIQPERVECRMNQL----LR

O.sativa (NP_001172359): SLTQGAESPP-------------YFVQVTMEVNH--WPEDGIQPERIESAINRV----LA

hPiezo1 (NP_001136336): ----------RPRDT----RRISLRFRRRKKEGPA--RKG-----------AAAIEAEDR

hPiezo2 (NP_071351): ----------EPSQR----S--SFSSNRSQRGSTS--TRN-----------S--------

E.histolytica (XP_649449): KIKKQKYCTIKPQHKETFDNSYSIINDDSDSLNDE--FDLLKQHEVELMNQNELEVTNII

E.histolytica (XP_655549): VINSQVCKKSKKVHKKTYDKKYCVNKDKILDAIDE--FECVNYHEVEMLSEKSLEVSEQM

L.major (XP_001686914): -------SAGSA---SL-S--------NSITAATTLLPSPSFLLLTPIS--------H--

L.infantum (XP_001469682): -------SAGGA---SL-S--------TSVTAATTVSPSASFLLLTPIS--------H--

T.cruzi (XP_819187): -------SSSVP---AIYSPS-SF---GDAASGTEQLPAGRSPMEDPIR--------RSR

T.cruzi (XP_812333): -------PSSFP---AIYSPS-SF---GDAAPGTEQLPAGRSPVEDPIG--------RSR

L.major (XP_001686223): -------CKGERDATANVSAA-AF-------ASLE--PIGP-PT-REAR--------QGK

L.infantum (XP_001468509): -------CKGEPDTTASVSAA-DF-------ASLE--PTGS-PT-REAR--------QDK

L.braziliensis (XP_001564414): -------REGERDAPVSVSAA-VL-------TSME--STGA-SA-HERR--------HDD

T.cruzi (XP_817508): -------NEGRPSRQAPLSSS-------SSSSTLE--LLANKHD-DNNV--------YNS

T.cruzi (XP_820998): -------NEGRPSRQEPLSAS-SS---SSSSSTLE--LLANKHD-DNNV--------TNS

T.vaginalis (XP_001319509): -------V--AS---QHFTFN-H------------P-PQIRNIKPS--------------

T.vaginalis (XP_001582897): -------C--AS---QTYAFT-H------------P-FQSRRPNQHKVV-----------

T.vaginalis (XP_001581503): -------C--AS---QTYSFT-H------------P-FQIRRPKQHPIE-----------

T.vaginalis (XP_001580012): -------CVFAP---SHYNNK-IFGGDNKLEGYPDK-YFYNFDNPDKVL-----------

T.vaginalis (XP_001305124): -------LVFSP---HHYTET-VFGGDNKLDGFPDK-YFYNFDNPDKVL-----------

N.gruberi (XP_002682879): ------------------ATSVELVTNDIMTATPR--PSNATNNE------------EKK

D.discoideum (XP_640187): NNNNNNNNNNNNNN----NNNNNNNNNNNNNNNNN--NNNNNNNNNIIIDQILVDCSNEY

D.rerio (XP_696355): ---------KNDGMK----KSHFLRFRKKKKAEPD--AVQ-----------KEPKKKKK-

D.melanogaster (AFB77909): ----------NDDAA----QKLSLQVS--QASL---------------------------

M.musculus (NP_001032375): ----------KPRHT----RHISIRFRRRK-ETPG--PKG-----------TAVMETEHE

R.norvegicus (NP_001070668): ----------KPQHR----RHISIRFRRRK-ETQG--PKG-----------AAVVEAEHE

B.taurus (XP_002694905): ----------EPGGK----RRISLRFRRRRKESTE--TRG-----------QAIGCWGSG

C.lupus (XP_546782): ----------KPRDI----KRISLRFRKRRRETTE--PVQ-----------PTATE----

A.thaliana (NP_182327): LVHNERCEKGNPDLC-PYSSRVHVQSIERSTETPN--EAL-------VV--LEVEYASPT

O.sativa (NP_001172359): IAHEERCQANSPSSC-HSCSRVRIQSIERSKENSS--MAL-------AV--LEVVYAAPL

hPiezo1 (NP_001136336): EEEEGEEEKE-A-PTGREKRPSRSGGR-------VRAAGRRLQGFCLSLAQGTYRPLRRF

hPiezo2 (NP_071351): -SQKGSSVLS-IKQKGK---RELYMEK-------LQEHLIKAKAFTIKKTLEIYVPIKQF

E.histolytica (XP_649449): NFEKLESYLH---------KREIYLKE--------QDEIYSLTNKYISFIKSIYLSIKTF

E.histolytica (XP_655549): SSDKLNNFLK---------SREEYLRE--------KELKCKKKNLIGKAFSTVYHPLKSF

L.major (XP_001686914): GTSIIGAFR---A-------------------------------------------LVAD

L.infantum (XP_001469682): GTSIISAFR---A-------------------------------------------LVAD

T.cruzi (XP_819187): TSCMMGIYRKVCL-------------------------------------------AVLG

T.cruzi (XP_812333): TSCMMGIYRKVCL-------------------------------------------AVLG

L.major (XP_001686223): PHRGVAELWRSCK-------------------------------------------RHLQ

L.infantum (XP_001468509): PHRGVAELWGSCK-------------------------------------------LHLH

L.braziliensis (XP_001564414): PHRGVAELCKICK-------------------------------------------RHLR

T.cruzi (XP_817508): NHDGIGEYNNSMRQKW---QKRVFKK----------------------------GGVLHK

T.cruzi (XP_820998): KHDGIGEYNNSMRQKW---QKRVFKK----------------------------GGVLQK

T.vaginalis (XP_001319509): ---------------------QTV-----------------------------KKTLSEK

T.vaginalis (XP_001582897): ------DPE----------VA-QP-----------------------------KLTFGEK

T.vaginalis (XP_001581503): ------EPQ----------TNQMP-----------------------------KLGLSEK

T.vaginalis (XP_001580012): ------GLS----------QKKMP-------------------------------SIWQQ

T.vaginalis (XP_001305124): ------GLS----------SKQTG-------------------------------TVWEQ

N.gruberi (XP_002682879): ESKKTVKFNV---------QQEEDKKG--------KKDNSEKPNIF----RRGLSKFINH

D.discoideum (XP_640187): DEEDQQQFNE-FYDEEFDDRNEQEKENDEQEIQVIKKSSKSIAKIIIYPFKWLFVSIIEY

D.rerio (XP_696355): ---------K-CKTKH---SKETSKKL-------LNAIEGKFKSLFLSVVKNVYRPTWDF

D.melanogaster (AFB77909): --------------PGS---PEFSKTG-------INQL----------ERTKYTSSLYKF

M.musculus (NP_001032375): EGE-GKETTE-RKRPRHTQEKSKFRER-------MKAAGRRLQSFCVSLAQSFYQPLQRF

R.norvegicus (NP_001070668): EGEEGREAAG-RKRLRRPREGLKIREK-------MKAAGRRLQSFCLSLAQSFYQPLRRF

B.taurus (XP_002694905): CRA-SACPCE-C-PAGS------AQAR-------VGGAGPGLS-PPARRARSMYWPVRRF

C.lupus (XP_546782): --------GV-V-VASRREKWSRPRER-------MTAMGLRLQTFCLAVAQSMYRPLRRF

A.thaliana (NP_182327): NGCSSAEWYK-SLTPASDVAKEIRKA---------QHSGLGEGTGF-------PYPIL--

O.sativa (NP_001172359): -DCQSAGWYK-SLTPAADVEKEIHES---------QKAGLFEDVNF-------PYPVV--

hPiezo1 (NP_001136336): FHDILHTKYRAATDVYALMFLADVVDFIIIIFGFWAF-GKHSAATD--ITSSLSDDQVPE

hPiezo2 (NP_071351): FYNLIHPEYSAVTDVYVLMFLADTVDFIIIVFGFWAF-GKHSAAAD--ITSSLSEDQVPG

E.histolytica (XP_649449): FTSITNDQFKRGKDLYIPMFICELLCLLFLVIFQGTF-----INSDGSFIEFFTSDYLPI

E.histolytica (XP_655549): FNSIANDQFKQGADFYIPLFISEALCFLFLIIFQGTF-----TNIQGSFIKFFTDDYLPI

L.major (XP_001686914): YCQNLLTVPGVGEDWYIFYTSVDSLALLMVAVYYSRIAG----SNNGTLQDHVQNNLLPG

L.infantum (XP_001469682): YSQNLLTVPGVGEDWYISYTSVDSLALLIVAVYYSRIAG----NNNGTLQDNVQNNLLPG

T.cruzi (XP_819187): FVRNATSIPGVGRDWYIYYSTVEAIALLVFVFGYNKLSG----WVEDTLQESVKRNLLPG

T.cruzi (XP_812333): FVRNATSIPGVGRDWYIYYSTVEAIALLVFVFGYNKLSG----WVDDTLQESVKRNLLPG

L.major (XP_001686223): CWS--AHRCGAGADYYAAQLFFDSLSLALFGWAYYAIVPEDIATSEGNFLHAVQQNHLPG

L.infantum (XP_001468509): CWS--VHRCGAGADYYAAQLFFDGLSLALFGWAYYAIVPEDTATSEGNLLHAVQQNHLPG

L.braziliensis (XP_001564414): CWN--AHRCGAGADYYAAQLFFDGLSLVLFGCAYYAIVPEGAAISEGNLLHAVQQNHLPG

T.cruzi (XP_817508): YWNNICERYGTGSDYYTLQFWADGLSLIVFTVVYFLLAG----DFRGSILYSVQQDLLPW

T.cruzi (XP_820998): YWSNICERYGTGSDYYTLQFWADGLSLIVFTVVYFLLAG----DFRGSILYSVQQDLLPW

T.vaginalis (XP_001319509): IKERINMKSFVKYHFNKLCFTIDITAFILSLFSYLGWTIS---SSANSM--LSGSATITY

T.vaginalis (XP_001582897): LLRRINMKSFIQYSFNKVVLLIDVIAFCIALFFYLSWTVS---SDAISF--FSGSATITY

T.vaginalis (XP_001581503): LLSRINMKSFIKYAFNKIALLIDVIAFVIALFFYMSWTRS---SDANSF--IHGSSSVTY

T.vaginalis (XP_001580012): IVANANNIRIPTSNEYFIVMIIDAIAFLVMIFNISSWTTD---TSSD-----KTSTGISI

T.vaginalis (XP_001305124): IKQNANSIRIFPSTQYILVIIIDIIAFVIMLFGIDDWTTK---PRSTAVLLDFSKDGISL

N.gruberi (XP_002682879): FKGLTNDQFKLGIDYYVVTVAVEILAFVFFFFSFSSM----SGRAADETLGAIKSNELSG

D.discoideum (XP_640187): VWLAIRTDEKPGRDYYMPLLFTDFACLFFLVIFPQNF----TGIPSSDIAEFLEQNVIPR

D.rerio (XP_696355): FQNILHAEYRASTDVYALMFLTDVVDFIIIIFGFWAF-GKHSAAAD--IASSLSEDQVPE

D.melanogaster (AFB77909): FFSLVH-KSRLATDVYALMFLCDFVNFFVLLFGFTAF-GTQQTESDEGVQTYLAENKVPI

M.musculus (NP_001032375): FHDILHTKYRAATDVYALMFLADIVDIIIIIFGFWAF-GKHSAATD--IASSLSDDQVPQ

R.norvegicus (NP_001070668): FHDILHTKYRAATDVYALMFLADIVDIVVIIFGFWAF-GKHSAATD--IASSLSDDQVPQ

B.taurus (XP_002694905): FQDILHTKYRAATDVYALMFLADVIDFIIIIFGFWAF-GKHSAATD--ITSSLSDDQVPE

C.lupus (XP_546782): FDDILHTKYRAATDVYALMFLADVVDFIIIIFGFWAF-GKHSAATD--ITSSLSDDQVPE

A.thaliana (NP_182327): ---SVIGGGKRDTDLYAYIFGADLIVFFLVAIFYQSV----IKNKSEFIDVYQLEDQFPF

O.sativa (NP_001172359): ---SVIGGGKREIDLYAYYFGADLAVFFLVAMFYQSV----LKNKSEFLEVYQLEDQFPK

: :

hPiezo1 (NP_001136336): AFLVMLLIQFSTMVVDRALYLRKTVLGKLAFQVALVLAIHLWMFFILPAVTERMFN----

hPiezo2 (NP_071351): PFLVMVLIQFGTMVVDRALYLRKTVLGKVIFQVILVFGIHFWMFFILPGVTERKFS----

E.histolytica (XP_649449): FYVLGLLLQFIMILIDRIIYLCKSIKAKLIMQYFSLILYHVLIFIIYPSILQTKTR----

E.histolytica (XP_655549): TYVLGLFLQFAMILADRIIYLCKSIKAKLFMQYFSLLLYHILIVIVYPSFVETKTK----

L.major (XP_001686914): PMALLICVSVLQLVMDRMLYVQRSMRLKALANGVCAISYTVFYWWWRNAVTVSEH-----

L.infantum (XP_001469682): PMALLICVSVLQLVMDRMLYVQRSMRLKAIANGVCAISYTLFYWWWRNAVTVSEH-----

T.cruzi (XP_819187): PMVTVVLVSILYMIADRMLYVTQCMKGKLLLNTVTGVAYCILYMLWGNLLTVSSR-----

T.cruzi (XP_812333): PMVTVVLVSILYMIADRMLYVTQCMKGKLLLNTVTGVAYCILYMLWGNLLTVSSR-----

L.major (XP_001686223): IFVATALGLVVVLFLERILYVLHALFAKYLLHFFLAAVYHVLYVLWRAAQESKGGSGATP

L.infantum (XP_001468509): IFVATALGLVVVLFLERILYVLHALFAKYVLHFFLAAVYHVLYVLWRTAQESKGGSGATA

L.braziliensis (XP_001564414): IFVVTALGFVVVLFLERILYVLRALVAKYVLHFFLATVYHTLYVLWHTVQESKRGRGATT

T.cruzi (XP_817508): QLVLILFAGVMLMAAERIVYVLHSVQLKFVLHVVTILAYHAIFMLWRLTLAHRST-----

T.cruzi (XP_820998): QLVLILFAGVILMAAERIVYVLHSVQLKFVLHVVTILAYHAIFMLWRLTLAHRST-----

T.vaginalis (XP_001319509): DFVFFLMGNFLFILLIQWVIISEHKSWLFSFNLLYSIFTFVYMSFLIPVFTNNGCF----

T.vaginalis (XP_001582897): DFVFFLMGNFIFVLLNQWVILSKHKLWLFTFNFLYGIFTFIYMTYLIPVFTDNSCF----

T.vaginalis (XP_001581503): DFVFFLMGNFIFILLNQWVILSKHKLWLFIYNFLYSVFTFIYMTYLIPVFTDNGCF----

T.vaginalis (XP_001580012): FFVFSLIFSAIFIFVFFLLRISNNFLSLYIFNTIQLICVLIF--------DFWVCY----

T.vaginalis (XP_001305124): KAIFSLIFSTIFVLIYYFLRISNNFLGIFIFNIIQLLCILFF--------YFIVCV----

N.gruberi (XP_002682879): TFVIILFLFFIEICIERVLYLFATATGKLIFHFLVVLIYHIVYIAFFVEVSRNQNT----

D.discoideum (XP_640187): QYIVILLAQFGVIILDRIIYLYKSVKAKFVLQIVLTVLYHVFLFFYFPDLIVKPFS----

D.rerio (XP_696355): AFLVMLLIQFSTMIIDRALYLRKSILGKLIFQVILVFGIHLWMFFILPAVTERMFN----

D.melanogaster (AFB77909): PFLIMLLVQFLLIVIDRALYLRKALVNKIIFHFFSVIGIHIWMFFVVPAVTERTFN----

M.musculus (NP_001032375): AFLFMLLVQFGTMVIDRALYLRKTVLGKLAFQVVLVVAIHIWMFFILPAVTERMFS----

R.norvegicus (NP_001070668): AFLFMLLVQFGTMVIDRALYLRKTVLGNLAFQVVLVVAIHLWMFFILPAVTERMFR----

B.taurus (XP_002694905): AFLVMLLIQFSTMVIDRALYLRKTVLGKLAFQVVLVLAVHLWMFFILPAVTERMFS----

C.lupus (XP_546782): AFLVMLLIQFSTMVIDRALYLRKTVLGKLAFQVVLVLAIHLWMFFILPAVTERMFN----

A.thaliana (NP_182327): DFVIILMVIFFLIVVDRVIYLCSFATGKVVYYLFSLILFTYAVTEYAWSIYPTQQH----

O.sativa (NP_001172359): EFVFILMILFFLIVVDRIIYLWSFATGKVIFYLFNLVLFTYSVTEYAWGMELVHRN----

: : :

hPiezo1 (NP_001136336): -QNV--VAQLWYFVKCIYFALSAYQIRCGYPTRI--LGNFLTKKYN--HLNLFLFQGFRL

hPiezo2 (NP_071351): -QNL--VAQLWYFVKCVYFGLSAYQIRCGYPTRV--LGNFLTKSYN--YVNLFLFQGFRL

E.histolytica (XP_649449): -LSSI-CLTIFYVFKVVYWTISGLQIKSGYLILS--SKRILMANYS--YISSLIFSTYYS

E.histolytica (XP_655549): -IVKA-CLTIFYLFKVVYWIISGLQIRAGYNILS--SKRILMTNYS--FYSQMIFHTYYT

L.major (XP_001686914): -----ATGNVYFALKVVALVLSVTQVCRGFPVHR--RRHIFTTHLG--SLLSYSFTVYRS

L.infantum (XP_001469682): -----ATGNVYFALKVVALVLSVTQVCRGFPVHR--RRHIFTTHLG--SLLSYSFTVYRS

T.cruzi (XP_819187): -----VVGNLFFTLKILALTIAVIQVRVGYPRHR--RHDPFTWTAGRSGIAGYLYFWYRC

T.cruzi (XP_812333): -----VVGNLFFTLKILALTIAVIQVRVGYPRHR--RHDPFTWTAGRSRIAGYLYFWYRC

L.major (XP_001686223): SAATTASVSLLLSAKFASLWCGALQLRHGYALHH--LHDPFTIKTDLLHWL--GHVSFRA

L.infantum (XP_001468509): SAATTASVSLLLSAKLASLWCGALQLRHGYALHH--LHDPFTIKTDLLHWL--GHVSFRA

L.braziliensis (XP_001564414): SVATTASVSLLLAAKFASLWCSALQLRHGYALHR--LHDPFTIKTDLLHWV--GHVTFRA

T.cruzi (XP_817508): ----TA-AVIILLMRFFSGSVSALQLQKGYPFHR--KHDPFTTHTDIFHWL--GHVVYRA

T.cruzi (XP_820998): ----TV-AVIILLMRFFSGSVSALQLQKGYPLHR--KHDPFTTHTDIFHWL--GHVVYRA

T.vaginalis (XP_001319509): -E--HFTFWLFFFLRLTSEMILSINCLFGFCKVPPSIGNPNPLVVY--VKIL----IVNA

T.vaginalis (XP_001582897): -E--HSTFWIFYFLRILSEIIYSCNIMFGFARMPPSLGNPNALFTY--IKLL----TITK

T.vaginalis (XP_001581503): -E--HASYWTFYFLRILSVIVYSCNLMFGFARMPPSLGNPNPLFTY--IKLL----TISK

T.vaginalis (XP_001580012): -KPKASSLQFYLFLKYISILIVYHDTYLGK--IIANFSFPDIENNW--RKILCMNNFMRI

T.vaginalis (XP_001305124): -N-RISSLEFYMFIKFVSILIVFHDTYCGK--LFANFSFPDIKKNG--SKILAMNNVIRI

N.gruberi (XP_002682879): -IGLV-LLKILFFIKCVYLFLGCLQIRTGYPKRR--YTQFFTKSYY--YIAQYVYLVYRA

D.discoideum (XP_640187): -FGYTWPLVVFYLMKCIYLYYSALQICYGYPILS--QNRFLMDGYS--DFHNIGYALYKA

D.rerio (XP_696355): -HNS--VAQLWYFFKCIYFTLSAYQIRCGYPTRI--LGNFLTKKFN--HLNLFLFQGFRL

D.melanogaster (AFB77909): -SLA--PPIIFYVIKCFYMLLSSYQIKSGYPKRI--LGNFFTKGFS--MVNMIAFKVYMQ

M.musculus (NP_001032375): -QNA--VAQLWYFVKCIYFALSAYQIRCGYPTRI--LGNFLTKKYN--HLNLFLFQGFRL

R.norvegicus (NP_001070668): -QNA--VAQLWYFVKCIYFALSAYQIRCGYPTRI--LGNFLTKKYN--HLNLFLFQGFRL

B.taurus (XP_002694905): -QNA--VAQLWYFVKCIYFALSAYQIRCGYPTRI--LGNFLTKKYN--HLNLFLFQGFRL

C.lupus (XP_546782): -QNA--VAQLWYFVKCIYFSLSAYQIRCGYPTRI--LGNFLTKKYN--HLNLFLFQGFRL

A.thaliana (NP_182327): --AAGLALRIIFLAKAMSLALQAIQIRYGLPHKSTLYRQFLTSEVS--RINYYGYRLYRA

O.sativa (NP_001172359): --VGGFVLRAIYLTKSISLALQALQIRYGIPNKSNLYRQFLTSKVT--QVNYFGFRLYRA

: : *

**_PFEW motif_**

hPiezo1 (NP_001136336): VPFLVELRAVMDWVWTDTTLSLSSWMCVEDIYANIFIIKCSRETEKKYPQPKGQKKKKIV

hPiezo2 (NP_071351): VPFLTELRAVMDWVWTDTTLSLSSWICVEDIYAHIFILKCWRESEKRYPQPRGQKKKKVV

E.histolytica (XP_649449): LPFVYEIRTILDWTFAKTSMFYKLWLKVEDIHAELYMNQCDREIEKARNHVYGESRGIME

E.histolytica (XP_655549): LPFVYEIRTILDWCLSKTSMFYKQWLKVEDIHAELYMNQCDRVIERNRNHVYGQPRGIVE

L.major (XP_001686914): LPFLWELRTLVDWTVLRTSLSLQEYLTVEDIYVYIYQCRDRYLEKHRNNEKLGDAVAPLS

L.infantum (XP_001469682): LPFLWELRTLVDWTVLRTSLSLQEYLTVEDIYVYVYQCRDRYLEKHRNNEKLGDAVAPLS

T.cruzi (XP_819187): LPFLWEIRVVTDWTVEKTSLHLDNYLKIEDLYDIVFERQCKLYNVCKQRQALGTPIPRST

T.cruzi (XP_812333): LPFLWEIRVVTDWTVEKTSLHLDNYLKIEDLYDIVFERQCKLYNVCKQRQALGTPIPRST

L.major (XP_001686223): VPFLMELRVMLDWSFSATTLKVQHWMLLEDIHHTVYRRYVDMHDLYHTSRHHGRHFPYLV

L.infantum (XP_001468509): VPFLMELRVMLDWSFSATTLKVQHWMLLEDIHHTVYRRYVDMHDLYHTSRYHGRHFPYLV

L.braziliensis (XP_001564414): VPFLMELRVLLDWSFSATALKVQHWMLLEDIHHTVYRRYVDMHDLHYTSHHQGRSFPYLV

T.cruzi (XP_817508): IPFLFELRLLLDWSVSCTALKLQHWMLLEDVHHTVYMRYVDINDLAWTSPRKGRQFPFFV

T.cruzi (XP_820998): IPFLFELRLLLDWSVSCTALKLQDWMLLEDVHHTVYMRYVDINDLAWTSPRKGRQFPFFV

T.vaginalis (XP_001319509): VPFLFEFIYLIKWLASTTSLDLFDFFIISQMKSKFMKQL---SYLKLWPL---DNK--KK

T.vaginalis (XP_001582897): TPFLFEFIQLTKWLACTTSLNMFDFMIIGQLRSKLSKQV---ALLKLFPA---NSK--KK

T.vaginalis (XP_001581503): VPFLFEFIQLTKWLACTTSLNMFDFMIIGQLRSKLCKQV---ALLKLFPA---NSR--KK

T.vaginalis (XP_001580012): CPFVFEIHSLLMWMSKDTRVEFAHFFIQEDIKTLLENQM---VYNHRPEQ---KRL--ER

T.vaginalis (XP_001305124): VPFIYEIYTLLVWMSKTTYVSYRKFFVMSELKIYLENQM---VYNHRHKE---EELKHQR

N.gruberi (XP_002682879): IPFVFELKTLLDWTFIDTTLTFYEYLKLEDVYSQLYKRKCDLEYKILQGKGFGNRIFLTT

D.discoideum (XP_640187): IPFVYELRTLLDWIATDTTMLFYDWLKFEDLYSTIFSVKCRLEWIKRQGRQKGHKQPKFE

D.rerio (XP_696355): VPFLVELRAVMDWVWTDTTLSLSNWMCVEDIYANIFIIKCSRETEKKYPQPKGQKKKRIV

D.melanogaster (AFB77909): IPFLYELRTILDWVCIDSTMTIFDWLKMEDIFSNIYLIRCTRQSETDFPAMRAQKKASLS

M.musculus (NP_001032375): VPFLVELRAVMDWVWTDTTLSLSNWMCVEDIYANIFIIKCSRETEKKYPQPKGQKKKKIV

R.norvegicus (NP_001070668): VPFLVELRAVMDWVWTDTTLSLSNWMCVEDIYANIFIIKCSRETEKKYPQPKGQKKKKIV

B.taurus (XP_002694905): VPFLVELRAVMDWVWTDTTLSLSNWMCVEDIYANIFIIKCSRETEKKYPQPKGQKKKKIV

C.lupus (XP_546782): VPFLVELRAVMDWVWTDTTLSLSSWMCVEDIYANIFIIKCSRETEKKYPQPKGQKKKKIV

A.thaliana (NP_182327): LPFLYELRCVLDWSCTATSLTMYDWLKLEDVNASLYLVKCDTVLNR-ATHKHGEKQTKMT

O.sativa (NP_001172359): LPFLYELRCVLDWSCTTTSLTMYDWLK---------------------------------

**: *: : * : : ::

∆ ○

hPiezo1 (NP_001136336): KYGMGGLIILFLIAIIWFPLLFMSLVRSVVGVVNQPIDVTVTLKLG---GYEPLFTMSAQ

hPiezo2 (NP_071351): KYGMGGMIIVLLICIVWFPLLFMSLIKSVAGVINQPLDVSVTITLG---GYQPIFTMSAQ

E.histolytica (XP_649449): KLTGGCIMIIIMLSILWFPLLLMSS-AAPNFIQPKPTNVEITFSLA---GNGKFFDQEDS

E.histolytica (XP_655549): RITSGFIMVIVLLAILWFPLLFMSS-AAPNFTQPKPTNIEVVLNFV---GQGELYKQQQS

L.major (XP_001686914): KWSFGVSRLALVLLALLGPLLYYST-YNPSTVANSATQLNFQLSFF---GTYDFFATTVR

L.infantum (XP_001469682): KWAFGVSRLALVLLALLGPLLYYST-YNPSTVANSATQLNFQLSFF---GAYDFFATTVR

T.cruzi (XP_819187): KVTAGLSRLVLFVGALMVPLVYYST-FNPSMQSNEVTQLRVGLSFM---SSQPFYASTTF

T.cruzi (XP_812333): KVTAGLSRLVLFVGALMTPLVYYST-FNPSMQSNEVTQLRVGLSFM---SSQPFYASTTF

L.major (XP_001686223): RLYQGVLGFTVILFVLFFPLFWYST-FSPQVRASHVTAWATEVALA---RMSSVPLFSAD

L.infantum (XP_001468509): RLYQGVLGFTVILFVLFFPLFWYST-FSPQVRASHVTAWATEVALA---RMSSLPLFSAD

L.braziliensis (XP_001564414): RLYQGVFGFTVILLVLFFPLFWYST-FSPQVRASHVTGWSTEVTFA---RMSPVPLFSAD

T.cruzi (XP_817508): RMYQGIVGFAACLLVLFFPLMLYST-FNPNVGVNLVTSWQTKIAFG---TTSNFY--TAT

T.cruzi (XP_820998): RMYQGIVGFAACLLVLFFPLMLYST-FNPNVGVNLVASWQTKIAFG---TTSNFY--TAT

T.vaginalis (XP_001319509): SHIIGYLFILALFALLFVPFIVMMS-SSSTSKPNPVKVASVSIGIY---GLPEIWSGTVL

T.vaginalis (XP_001582897): RHTLGIFFVIGLFCLLFVPFVVMMS-SSSTSIPNGVKVATVSLGIY---GLPELFTGTVL

T.vaginalis (XP_001581503): SHVLGILFMIAMFCLLFAPFIVMMS-SSTTSITNNVQVASVSLGIY---GLPELFTGTVL

T.vaginalis (XP_001580012): NLCIGGCFLFLIILLLFGPLFFMVG-GSGAYMNNPILSSELEIGFT---AVGAIYRSYAQ

T.vaginalis (XP_001305124): SGLIGTIFILLIIIVLFGPMFFLIK-SKISTEPNPIYSASLECGLS---STGYLYKSYAH

N.gruberi (XP_002682879): KATSGLLLFIVFAMIIFFPLLFYST-ANPALSYNTVNSIQFSIGIE---GFEPFYQNEYF

D.discoideum (XP_640187): KFVTGVTFFIGLVILLWFPLIILSS-GLPGSNIEPVNNIQIEVSVV---GWNPFLKINQD

D.rerio (XP_696355): KYGMGGLIILFLICIIWFPLLFISLVKSVVGVVNHPVDVTVTVKLG---GYEPLFTMSVQ

D.melanogaster (AFB77909): KLIMGGTIVLLIVICIWGPLCLFAL-GNAVGTSNVPFHVSLSIRIG---PYDPIYTTNNY

M.musculus (NP_001032375): KYGMGGLIILFLIAIIWFPLLFMSLIRSVVGVVNQPIDVTVTLKLG---GYEPLFTMSAQ

R.norvegicus (NP_001070668): KYGMGGLIILFLIAIIWFPLLFMSLIRSVVGVVNQPIDVTVTLKLG---GYEPLFTMSAQ

B.taurus (XP_002694905): KYGMGGLIILFLVAIIWFPLLFMSLVRSVVGVVNQPIDVTVTLKLG---GYEPLFTMSAQ

C.lupus (XP_546782): KYGMGGLIILFLVAIIWFPLLFMSLVRSVVGVVNQPIDVTVTLKLG---GYEPLFTMSAQ

A.thaliana (NP_182327): KCCNGICLFFILLCVIWAPMLMYSS-GNPTNIANPIKDASVQIDLKTVGGKLTLYQTTLC

O.sativa (NP_001172359): ---------------------IYSS-GNPTNIANPIIDVSVKIDIKALGGRLTFFKTTVC

hPiezo1 (NP_001136336): QPSIIPFTAQAYEELSRQFDP-------QPLAMQFISQYSPEDIVTAQIEGSSGALWRIS

hPiezo2 (NP_071351): QSQLKVMDQQSFNKFIQAFSR-------DTGAMQFLENYEKEDITVAELEGNSNSLWTIS

E.histolytica (XP_649449): --NFIELTGA-------EWKV-------LGQTHDLK-KDASLWIFGCSLSLKSMSYWSLT

E.histolytica (XP_655549): --VFKEITKT-------EWNE-------LQKDHSSLSLLSGEAIYSSQLSSDAMTYWLLT

L.major (XP_001686914): DNVTTP-------EGWWTWIERTRPTLGSYG---F--MAEGKTVQLMEFTSCSSSLWMAS

L.infantum (XP_001469682): DNVTTP-------EGWWTWIERTRPTLGSYG---F--MAEGKTVQLMEFTSCSSSLWMAS

T.cruzi (XP_819187): TAEEIP-------NEWSLWLARTRPSLDRNG---I--MNTKKTLQLVNVSSCSNELWPIS

T.cruzi (XP_812333): TAEEIP-------NEWFLWLARTRPSLDRNG---I--MNTKKTLQLVNASSCSNELWPIS

L.major (XP_001686223): A-AVRPFSFHAAATI-----SLGGASVGAAGLLRF--AAVTGTWQTAHVASCSTRMWSYT

L.infantum (XP_001468509): A-AVRPSSFHAAATL-----SLGGASVGAAGLLRF--AAVTDTWQTVHVASCSIRMWSYT

L.braziliensis (XP_001564414): S-TVCPSSFHEAAAL-----FGDGTVVRAEKLLRY--AAATDTWQTAHVAPCSTRMWSYT

T.cruzi (XP_817508): A-TEAPVSQNLVSILWELLPSLQHDEFGSQG-----------LLQLLMFPRCSAEVWSAT

T.cruzi (XP_820998): A-TEVSVSQNLVSILWDLLPSLQHDEFGSQG-----------LLQLLMFPRCSAEVWSAT

T.vaginalis (XP_001319509): PDESNIFSDTMQR----EILNL------NDPMLDAFYSNNKKESQYFQYPDITLTNWQIS

T.vaginalis (XP_001582897): PDDTTLMTDSMQK----HVMTM------NDKTLEPFFANSRKESQYFTYPSITMTNWIIS

T.vaginalis (XP_001581503): PNEDSFITNSMQK----KIMAM------NDQKLDPFFANSKRESQYFNYPGITMTNWIIS

T.vaginalis (XP_001580012): FQ---NLTSSEHN----DLANS------GFADLSVLKTYSRDDLIIALFPLSS----QST

T.vaginalis (XP_001305124): IM---PMTTAQQN----ELIKD------S-DEFDYLRAIPTDNMYIIDFPLES----QLV

N.gruberi (XP_002682879): --KVNPEKSLET---FVDFYD-------GNGLKLFPNYDPTFWIQNFTLAPYSERYWLIS

D.discoideum (XP_640187): --LSLESGDGDSTMDQNSFNN-------LKEDYSFLTTDDRQGIQNISINTFSEEIWNLS

D.rerio (XP_696355): QQSIQPFTESRYNQLNNQFSK-------NAVAMQFITMYSYEDIVTANIEGSSGSVWRIS

D.melanogaster (AFB77909): -DSIFEINPEMYSQMTNAYIK-------EKQALTFIAGYDATDVAAVRLAGNSPSLWNIA

M.musculus (NP_001032375): QPSIVPFTPQAYEELSQQFDP-------YPLAMQFISQYSPEDIVTAQIEGSSGALWRIS

R.norvegicus (NP_001070668): QPSIVPFTPEDYEELSQQFDP-------YPLAMQFISQYSPEDIVTAQIEGSSGALWRIS

B.taurus (XP_002694905): QPSIVPFTHHAYEELSKQFDP-------HPLAMQFISQYSPEDIVTVHIEGSSGALWRIS

C.lupus (XP_546782): QPSIVPFTQQAYEELSRQFDP-------NPLAMQFISQYSPEDIVTAQIEGSSGALWRIS

A.thaliana (NP_182327): -----ERISG----DNIDLGL-------DLGSQSFLPTYNKNDIQLICCQADASVLWLVP

O.sativa (NP_001172359): -----EKIPW----KHMRAYD-------DVDPLDYLGGYNVEDIQLICCQPDASTMWLIP

:

hPiezo1 (NP_001136336): PPSRAQMKRELYNGT---ADITLRFTWNFQRDLAKGGTVEYANEKHMLAL-----APNST

hPiezo2 (NP_071351): PPSKQKMIHELLDPN---SSFSVVFSWSIQRNLSLGAKSEIATDKLSFP-------LKNI

E.histolytica (XP_649449): PDKQEDLNETVCN-G---KQVILKTKIFMSREDSAVANTFELN--EKTS-------LSKK

E.histolytica (XP_655549): PQKKKELNTNLLA-D---ETLTLTYSISMSRESSAASNSFKFS--GSYE-------MNTE

L.major (XP_001686914): PQAVRQVLAGLRAAASNASSAYMLQTLEVSRSVSSTDAAMAASLVNHWP-------IPQD

L.infantum (XP_001469682): PQAVRQVLAGLRAVASNASSAYMLQTLEVSRSVSSTDAAAAASLVNRWP-------IPWD

T.cruzi (XP_819187): PQAYGRLNEQLMEVMENKTTMQLFQTIELSRSGTSN----FVSHTQSWT-------FSWE

T.cruzi (XP_812333): PQAYGQLNEQLMEVMENKTTMQLFQTIELSRSGTSN----FVSHTQSWT-------FSWE

L.major (XP_001686223): PAALQELVDRLGKRTEVVTLLVR---HRVTRNRASEAAMITVGLEESYA-------LPPT

L.infantum (XP_001468509): PAALQELVDLLGKRTEVVTLLVR---HRVTRDRASDATMTTVGLEESYA-------LPPT

L.braziliensis (XP_001564414): PAALWELLDRLGKTADAVTLVVR---NRVTRDRASEPTRIAVDFEESYT-------LPPA

T.cruzi (XP_817508): PETRLLLLGQLNAAVNNETTFFIHKEDHVVRMEVSAPETRRVRSYQRYM-------VPWS

T.cruzi (XP_820998): PETRLLLLSQLNAAVNNETTFFIHKEDHVVRMEVSAPATRRVCSYQRYM-------VPWS

T.vaginalis (XP_001319509): DSSANYALNKM-----TKFIPYVNFKFTTTSPTTKLTT-------DQITITKTGNNLDEE

T.vaginalis (XP_001582897): ESSARFAMNTIKNTSNPIFIPYVTFKFTVQTPTTKKNV-------DQITITKNGKQLKPE

T.vaginalis (XP_001581503): ESSARFAMYTIKNTTNPIFIPYVTFKFTVQTPTTKKNI-------DQITITKNGKQLSEK

T.vaginalis (XP_001580012): AVATDASIKSFLTNSNLNKRPYVKLTTTFQYPAT--TAKSN---VISYQLNMD--PITIS

T.vaginalis (XP_001305124): PELPNETIDTIFKSNDYEIVYTLN--LNFLKGTTNSFSPNV---VQQYQLFNE--TLSLQ

N.gruberi (XP_002682879): TPSRMSMVEALAN-----ETLALNLTMSLLVKRSGPSTQLELETRQSIV-------LNAW

D.discoideum (XP_640187): PPAKAQLINYLLT-N---TSLQIEVSYTLTR--SGGVNSVIVGS-NSVQ-------LKPD

D.rerio (XP_696355): PPSRQELIKELLSST---GDMTLRLDWNFQRDLGKGGTVENTFDKHSISL-----APKNP

D.melanogaster (AFB77909): PPDRQRLLNDLRN-N---HTLKARFSYSLTRKAPAKGLKENVGDEHAISL-----DESFE

M.musculus (NP_001032375): PPSRAQMKQELYNGT---ADITLRFTWNFQRDLAKGGTVEYTNEKHTLEL-----APNST

R.norvegicus (NP_001070668): PPSRAQMKHELYNGT---ADITLRFTWNFQRDLAKGGSVEYTNEKHTLEL-----APNST

B.taurus (XP_002694905): PPSRAQMKRELYNGT---ADITLRFTWNFQRDLAKGGSVEYTNEKHTLDL-----APNST

C.lupus (XP_546782): PPSRAQMKRELYNGT---ADITLRFTWNFQRDLAKGGTVEYTNEKHTLDW-----APNST

A.thaliana (NP_182327): DTVVTRFIQSLDWD----TDMDITFTWVLNRDRPKGKETVKYER--SVD------PLDLP

O.sativa (NP_001172359): APVQTRFIQSLEETEMIFGNMELILNWDFLRARPKGKELVKYES--PVD------R--SP

hPiezo1 (NP_001136336): ARRQLASLLEGTS-------------------------------DQSVVIPNLFPKYIRA

hPiezo2 (NP_071351): TRKNIAKMIAGNS---------------------------TESSKTPVTIEKIYPYYVKA

E.histolytica (XP_649449): ELQGFCDLLN------GKEN-------------------NFTLSFFPE-------IVSM-

E.histolytica (XP_655549): EKKKLSTILN------NGTS-------------------DLYLSIFPQ-------ILKM-

L.major (XP_001686914): TAQDLVAILERETSGARGAAVVDSEN----SS------SSV--VVGSASLPFFYSPFVFN

L.infantum (XP_001469682): TAQDLVAILERETSGTRGAAVEDSEN----SSSSSSSSSSI--VVGSASLPFFYSPFVFN

T.cruzi (XP_819187): AARDIHRMLNNPTTNG----------------------------TGYVKLQNFYTPFLFS

T.cruzi (XP_812333): AARDIHRMLNNPTTNG----------------------------TGYVKLQNFYTPFLFS

L.major (XP_001686223): SQALLAEAL---NAWRTM-------DDADSAGTAEYSASAW--DPIEVPLPLLYSPYVLS

L.infantum (XP_001468509): SQALLAEAL---KAWRAM-------DDADSAGTAEYSASAW--DPVVVPLPLLYSPYVLS

L.braziliensis (XP_001564414): SQALLAEAL---KAWHAA-------DSADSADTTDYLASPW--RTVVVPLPSLYAPYVLS

T.cruzi (XP_817508): TVVKLRDALEGAHLWYTSRHLEKHQGGVHSASTLVYESGDW------IPLPQFYSPFLNN

T.cruzi (XP_820998): TVVRLRDALEGAHLWYTSRHLEKHQGGVHSASTLVYESGDW------IPLPQFYSPFVNN

T.vaginalis (XP_001319509): QIKTLKEIINCTL------------------------NTQEP-ENLSLNIENLIPLYFTI

T.vaginalis (XP_001582897): VLETLYKALECTM------------------------NLATP-ENLSFYLDDLVPLFFII

T.vaginalis (XP_001581503): DLETLYKALECTV------------------------TSTSPQENLTFYLDSLVPVFFII

T.vaginalis (XP_001580012): QSSDYLEAW---K------------------------NISSY-T-NLTNVPVV---YFNP

T.vaginalis (XP_001305124): NKTMLYENY---L------------------------SHNGT-VMFNFSLPVLLMTFYSY

N.gruberi (XP_002682879): KRKQLAQMIN------YNVSKVNSEN-------L-KESIDIPLSYNPY-------MMNKY

D.discoideum (XP_640187): QELNFYNILTRVQSNNSNNSNNPNENSSSGSDDN-NNNSNNNIGSNSFIVEGLFYKFIKL

D.rerio (XP_696355): VRADLASLLLGTR-------------------------------KDPVHVPHMFPNYIRA

D.melanogaster (AFB77909): GRAALIHMLSETHDVEP----IHSNGTTNG---------TTPEVEEVVVIPGMIPKFIKV

M.musculus (NP_001032375): ARRQLAQLLEGRP-------------------------------DQSVVIPHLFPKYIRA

R.norvegicus (NP_001070668): ARRQLAQLLEGRP-------------------------------DQSVVIPHLFPKYIRA

B.taurus (XP_002694905): ARRQLASLLEGTS-------------------------------DQSVVIPHLFPKYIRA

C.lupus (XP_546782): ERRQLASLLEGTS-------------------------------DQSVVIPHLFPKYIRA

A.thaliana (NP_182327): KRSDIQMVLNG--------------------------------SMDGFRVHNLYPKFFRV

O.sativa (NP_001172359): SVDDVKRVLNG--------------------------------TINSFRITDAYPRYFRV

hPiezo1 (NP_001136336): PNGPEANPVKQLQPNEEADY-LGVRIQLRRE-QGAGAT---------GFLEWWVIELQEC

hPiezo2 (NP_071351): PSDSNSKPIKQLLS--ENNF-MDITIILSRDNTT------------KYNSEWWVLNLTGN

E.histolytica (XP_649449): PTLSQTELLR--------H--DSVNISL---------IPKKGIDNKTQLSYWWFESI---

E.histolytica (XP_655549): PTLKEKELLD-------AF--DNVNITL---------KPILHFDSVTNLYYWSFQQCVNV

L.major (XP_001686914): RAS-RIDKLTTSLHFP-HRNQHNCTLELNHGTD-ITL---------NSRVRYWCLRCAPL

L.infantum (XP_001469682): RAS-RIDGLPTSLRFP-HRNQHNCTLELNHNTD-VAL---------NSLVRYWCLHCAPL

T.cruzi (XP_819187): HPD-GLAAFDG---GG-GVNVMDCSILLRQEVD-SVL---------NNTVRYWCVECQSL

T.cruzi (XP_812333): HPD-GLAAFDD---GG-GANVMDCSILLRQEAD-SVL---------NNTVRYWCVECQSL

L.major (XP_001686223): YGT-GVSFLSG----A-TMRRANCSLTLHRTGRYRG---------------FSCVKCAEG

L.infantum (XP_001468509): YGT-GVSFLSG----A-TMRRANCSLTLHRMGRYRG---------------FSCVKCAEG

L.braziliensis (XP_001564414): SGA-GISFFSG----L-DVRGVNCSLTLHRVGKYSG---------------FSCLTCAED

T.cruzi (XP_817508): IQN-DLVM-------K-KHVRADCAVQLNGVGNSDGK---------TPPVLYWCVRCNSF

T.cruzi (XP_820998): IQN-DLVM-------K-NYVRTDCAVQLNGVGNSDGK---------TPPVLYWCVRCNSF

T.vaginalis (XP_001319509): PLSDEATFIN--------KYYFNLSMQFMKS--------------QSSGQFYWLISVNKT

T.vaginalis (XP_001582897): PLAKDASVVS--------NYYFNTTFTFEQS---------------TNGNFYWSISTKRT

T.vaginalis (XP_001581503): PLSADATAVS--------GYTFNVTFSFQQS---------------YSGNYYWSINAMRN

T.vaginalis (XP_001580012): REGAASSSST--------NYSC--GFQFD-----------------TTANVVQLATVN--

T.vaginalis (XP_001305124): PIMPTKSESQ--------IY----GMEFT-----------------ARGKFAFV--HR--

N.gruberi (XP_002682879): SALATATVME-------SRYLPNCSLTVT--NVYN--SSRTELVHNPPGFVSWFMSCTQP

D.discoideum (XP_640187): PGVSGNPIYP-------TDN-NGNVLALD--ALFTMNSTNFNNSNQPPQY-FWQVNAYDS

D.rerio (XP_696355): PSGAEAKPVMQLFDGDEEGF-QDVTVSLMREAS----------VNTTGAQEWWDISIADC

D.melanogaster (AFB77909): LNSGDAAVVSVLSP-KHYDY-RPLVIKMHRDNET--------------NGLWWEIRDYCN

M.musculus (NP_001032375): PNGPEANPVKQLQPDEEEDY-LGVRIQLRREQVGTGASGEQAGTKASDFLEWWVIELQDC

R.norvegicus (NP_001070668): PNGPEANPVKQLQPDEEEDY-LGVRIQLRREQVGTGTSGEQAGTKASDFLEWWVIELQDC

B.taurus (XP_002694905): PNGPEANPVKQLQPNEEADY-LGVRIQLRRERVGTGAA---------GFLEWWVIELQDC

C.lupus (XP_546782): PNGPEANPVKQLQPNEEADY-LGVRIQLRRERVGSGAA---------GFLEWWVIELQDC

A.thaliana (NP_182327): TGSGDVRSFEDQT--------DEVS-------------ADIL-INHANFKWWWSFHNLKA

O.sativa (NP_001172359): TGSGEVRRLEASI--------DSVS-------------GELL-LNNGTPPWWSFYD-T-N

hPiezo1 (NP_001136336): R---------------------------------------------------------TD

hPiezo2 (NP_071351): R-I-----------------------------------------------------YNPN

E.histolytica (XP_649449): ----N------------------------------------------------------G

E.histolytica (XP_655549): DELWN------------------------------------------------------C

L.major (XP_001686914): FPEGNIPSENSSSAAEWRCLTTGEGCDDFNYEDAAGGSGSRTVQASLAAQMNSGV--TAK

L.infantum (XP_001469682): FPEGNIPSENSSSAAEWRCLTTGEGCDDFNYEDATGGSGSRTVRASLAAQMNSRV--AAK

T.cruzi (XP_819187): FTHGNIPNSNKTRFPEWGCLSTGEGCSKFNFERRNGD-----------------V--NYN

T.cruzi (XP_812333): FTHGNIPNSNKTRFPEWGCLSTGEGCSNFNFERRNGD-----------------V--NYN

L.major (XP_001686223): GQSSP-----A--TATFAAATTANPAGS--------------------SQIASGAEIREM

L.infantum (XP_001468509): DQSSP-----A--ASTFAAATAADPAGS--------------------SQIASGAEIHEM

L.braziliensis (XP_001564414): GQRNT-----A--KNTLATDAPTSPAGS--------------------SKIASAVEIDEK

T.cruzi (XP_817508): ANLTD-----GSTTRTWSMNGANEPLDMADNDNDNGEAMQS------KETLVSDTNAG--

T.cruzi (XP_820998): ANLTD-----GSTTRTWSMNGANEPLDMADSDNDNVEVMQS------KETLESGTDAG--

T.vaginalis (XP_001319509): EDMPD----------------------------------------------------FVE

T.vaginalis (XP_001582897): AKQPV----------------------------------------------------DVQ

T.vaginalis (XP_001581503): ENLPD----------------------------------------------------DIK

T.vaginalis (XP_001580012): PNISE----------------------------------------------------LKD

T.vaginalis (XP_001305124): ESASS----------------------------------------------------LLA

N.gruberi (XP_002682879): TKFYP--------------------------LEKSGN-------------------LKSI

D.discoideum (XP_640187): TKTYN--------------------------IST--------------------------

D.rerio (XP_696355): K---------------------------------------------------------ES

D.melanogaster (AFB77909): DTFYN--------------------------ETLSKF------------------AYSNC

M.musculus (NP_001032375): K---------------------------------------------------------AD

R.norvegicus (NP_001070668): Q---------------------------------------------------------AE

B.taurus (XP_002694905): Q---------------------------------------------------------AN

C.lupus (XP_546782): Q---------------------------------------------------------AE

A.thaliana (NP_182327): SE--N-------------------------------I------------------SACEG

O.sativa (NP_001172359): PS--D-------------------------------L------------------AGCQG

○

hPiezo1 (NP_001136336): CNLLPMVIFS-DKV-SPPSL-GFLAG--YGIMGLYVSIVLVIGKFVRGFFSEISHSIMFE

hPiezo2 (NP_071351): SQALELVVFN-DKV-SPPSL-GFLAG--YGIMGLYASVVLVIGKFVREFFSGISHSIMFE

E.histolytica (XP_649449): STDVDWYVSS-PNVPDTSVI-SSLSS--KGIIGLYTVVVITLYSLIKSDYSGLSHTIMFK

E.histolytica (XP_655549): SEGIKFFISS-PKVPNSGIL-SALSS--LGIIGLYTVVVLALYSLIKSDYVGQAHTIMFK

L.major (XP_001686914): QVSVYMVIISDTVV---MGI-SLLKG--IGIVALYTTFVLALGRLLRSVLTNQTSTLLYS

L.infantum (XP_001469682): QVPMYMVIISGTVV---MGI-SLLKG--IGIVALYTTFVLALGRLLRSVLTNQTSTLLYS

T.cruzi (XP_819187): PVPMYFVVLSDPSV---GGV-PFLQG--IGIVALYTTFVLALGRIFRGMFANKAKYTILS

T.cruzi (XP_812333): PVPMYFVVLSDPSV---GGV-PFLQG--IGIVALYTTFVLALGRIFRGMFANKAKYTILS

L.major (XP_001686223): SSPLTFVVASNNVAIVQSSF-SLIPN--VGIIALYTSFVLVMSSYIRNYFAGDAHRVVLL

L.infantum (XP_001468509): STPLTFVVASNHVAIVQSSF-SLIPN--VGVIALYTSFVLVMSSYIRGCFAGDAHRVVLL

L.braziliensis (XP_001564414): STPLAFVIVSKNVAVVQSSF-SLIPS--VGIIALYTSFVLVMSSYIRNYFAGDAHRVVLL

T.cruzi (XP_817508): NITGIFEIVASSYVALNLSL-VFLPN--MGIIALYTTFILAIGTFLRNTVMDRAHRVVLM

T.cruzi (XP_820998): NITSIFEIVASSYVALNPSL-VFLPN--MGIIALYTTFILAIGTFLRNTVMDRAHRVVLM

T.vaginalis (XP_001319509): EGALSSAVYC-KPSY-NAVIGSLLTST-GGFYGLYVFIIFTCGTFVRSFITSFFQDLWID

T.vaginalis (XP_001582897): EGPIGSVVFS-KPTY-DAVISTLMSKTGGGFYGLYVFFTLSCGKMVRSFINSFFTDLWID

T.vaginalis (XP_001581503): EGPISSIVFS-KPTY-DETIGSILSKTGGGFYGMLLFVIMTCGKFIRSFINSFFSDLWID

T.vaginalis (XP_001580012): ATFLKIIIWS-DEVESDGTVG-TLVSMGGGIIGIYILIIFTFGEVLRERTLGGYDELWLE

T.vaginalis (XP_001305124): TPTIRVMLYS-SEV----------------------------------------------

N.gruberi (XP_002682879): TGGPFFFIQSTNIVAADSVI-SAISS--FGIITFYTSFVLAVAQFVRLYSSNLVGRITFE

D.discoideum (XP_640187): LESIQFYTIS-S-KLPNGIT-STLVS--AGIIGLYVSVVLSVGRFLRLSITQISIKIQLE

D.rerio (XP_696355): CNVLPMIIFN-DKV-SPPSL-GFLAG--YGIMGLYVSVVLVIGKFVRGFFSEISHSIMFE

D.melanogaster (AFB77909): TSGIVMYTFN-DKK-FPSTF-SFLTA--GGIIGLYTTFVLLASRFMKSFIGGQNRKIMFE

M.musculus (NP_001032375): CNLLPMVIFS-DKV-SPPSL-GFLAG--YGIVGLYVSIVLVVGKFVRGFFSEISHSIMFE

R.norvegicus (NP_001070668): CNLLPMVIFS-DKV-SPPSL-GFLAG--YGIVGLYVSIVLVVGKFVRGFFSDISHSIMFE

B.taurus (XP_002694905): CNLLPMVIFS-DKV-SPPSL-GFLAG--YGIMGLYVSIVLVIGKFVRGFFSEISHSIMFE

C.lupus (XP_546782): CNLLPMVIFS-DKV-SPPSL-GFLAG--YGIMGLYVSIVLVIGKFVRGFFSEISHSIMFE

A.thaliana (NP_182327): MDGPVAIIMS-EETPPQGFLGDTLSK--FSIWGLYITFVLAVGRFIRLQCSDLRMRIPYE

O.sativa (NP_001172359): LNGPMAIVVS-EET-PQGIIGETLSK--FSIWSLYITFVLAVARFIRLQCSDLRMRIPYE

●

hPiezo1 (NP_001136336): ELPCVDRILKLCQDIFLVRE------TRELELEEELYAKLIFLYRSPETMIKWTREKE--

hPiezo2 (NP_071351): ELPNVDRILKLCTDIFLVRE------TGELELEEDLYAKLIFLYRSPETMIKWTREKTN-

E.histolytica (XP_649449): NLPNCHGLLQLCDDIIMARQ------DGDLKLEEDLVDELIMIYRTPSLLLEKTKID---

E.histolytica (XP_655549): NLPNCLGLLQLCDDIIIARQ------DGDLRLEEDLVNELITIYRTPSLLFEKTK-----

L.major (XP_001686914): NMANPAALQNMVRCMEMARE------YGDLRLEHTIYLELVDLLRSAERLFRVTGPLRCM

L.infantum (XP_001469682): NMANPAALQNMVRCMEMARE------YGDLRLEHTIYLELVDLLRSAERLFRVTGPLRCM

T.cruzi (XP_819187): DMADPTPIAQLIEYITVARE------CGDLPLEQSVYIELVDLLRSPERLLRLTGFNRQM

T.cruzi (XP_812333): DMADPTPIAQLIEYITVARE------CGDLPLEQSVYIELVDLLRSPERLLRLTGFNRQM

L.major (XP_001686223): QLANPEPVTELLRYLYLARSSANDGQVGDLALEQLLFVELLDLLRSPERLLRLSGRRVDD

L.infantum (XP_001468509): QLANPEPVTELLRYLYLARSSANDGQVGDLALEQLLFVELLDLLRSPERLLRLGGRRVDD

L.braziliensis (XP_001564414): QLANPEPVAELLRYLYLARSSANSGQVGDLVLEQLLFVELLDLLRSPERLLRLGGRRVDD

T.cruzi (XP_817508): QVANPDPIAELLRYIYMARSSAINGYVDGLLLEQQLFFELVDMLRSPERVLALSGRRVDD

T.cruzi (XP_820998): QVANPDPIAELLRYIYMARSSAINGYVDGLLLEQQLFFELVDMLRSPERVLALSGRRVDD

T.vaginalis (XP_001319509): RMGNPEIFLNVILAIEAHRN------TGDLPMEYATATMILNTARSLHNMAKIGKTVKVK

T.vaginalis (XP_001582897): KMGKPEIFLNVILAIEAHRK------AGDLKSEYATAMMLLNTIRSVHNLVKIGGTLPID

T.vaginalis (XP_001581503): KMGKPQIFLNVILAIEAHRK------EGDLTSEYATAMMLLNTIRSVHNLVRIGGTVPIN

T.vaginalis (XP_001580012): RMRKPENLYQYIIAIEAFRS------VGAIDREYMMMEMFLDVMRSKEACITLTNEPESE

T.vaginalis (XP_001305124): ------------------------------------------------------------

N.gruberi (XP_002682879): DLDNVDLILCYCQDIYKARE------DKDLAMEETMFVNLLRIFRSTELLQIWTRDAKKN

D.discoideum (XP_640187): NLDSCDEILKMIDDVFIARE------YGDLVLEEELYHELIQVFRQPQLLYSLTTFKNNQ

D.rerio (XP_696355): ELPCADRILKLCMDIFLVRE------TGELELEEELYSKLIFLYRSPETMIKWTREKNHN

D.melanogaster (AFB77909): DLPYVDRVLQLCLDIYLVRE------ALEFALEEDLFAKLLFLYRSPETLIKWTRPKEEY

M.musculus (NP_001032375): ELPCVDRILKLCQDIFLVRE------TRELELEEELYAKLIFLYRSPETMIKWTRERE--

R.norvegicus (NP_001070668): ELPCVDRILKLCQDIFLVRE------TRELELEEELYAKLIFLYRSPETMIKWTREKE--

B.taurus (XP_002694905): ELPCVDRILKLCQDIFLVRE------TRELELEEELYAKLIFLYRSPETMIKWTRDKE--

C.lupus (XP_546782): ELPCVDRILKLCQDIFLVRE------TRELELEEELYAKLIFLYRSPETMIKWTREKE--

A.thaliana (NP_182327): NLPSCDRLIAICEDLYAARA------EGELGVEEVLYWTLVKIYRSPHMLLEY-------

O.sativa (NP_001172359): NLPSCDRLLDICEGIYAARA------EGELEVEEVLYWTLNINGLFEKLEIVAGPVGDIK

hPiezo1 (NP_001136336): ------------------------------------------------------------

hPiezo2 (NP_071351): ------------------------------------------------------------

E.histolytica (XP_649449): ------------------------------------------------------------

E.histolytica (XP_655549): ------------------------------------------------------------

L.major (XP_001686914): YGDAGHEDLFGLGQVRRR--NIHRNPS---VE-----AREGEAGEGHGDSGVPAAV--PH

L.infantum (XP_001469682): YGDAGHEDLFGLGQVRRR--NIRRNPS---VE-----AHEGEAGEGHGDSGVPAAV--PH

T.cruzi (XP_819187): YDAETDVIQS-----------PHATPS---NRMTENLSHRGDALRGPEAGIAPADDAKRE

T.cruzi (XP_812333): YDAETDVIQP-----------SHATPS---NRMTENLSHRGDALRGPEEGIAPADDAARE

L.major (XP_001686223): YAYST-YRQVLYDATR----RPFAPPG---EG------------RG--------------

L.infantum (XP_001468509): YAYST-YRQALYDATR----RPFAPPD---EG------------RG--------------

L.braziliensis (XP_001564414): YADST-YRQALFEATK----RPFSLPE---ER------------RR--------------

T.cruzi (XP_817508): YDGCGRFCKYLKKSSY----HPF-------------------------------------

T.cruzi (XP_820998): YDNCGRFCKYLKKSSY----HPF-------------------------------------

T.vaginalis (XP_001319509): EA----------------------------------------------------------

T.vaginalis (XP_001582897): QEEAIKGRGATFI-----------------------------------------------

T.vaginalis (XP_001581503): EGERVNYLL---------------------------------------------------

T.vaginalis (XP_001580012): EPSTK-------------------------------------------------------

T.vaginalis (XP_001305124): ------------------------------------------------------------

N.gruberi (XP_002682879): WI----------------------------------------------------------

D.discoideum (XP_640187): LNLPT---------------TPT-INSTLNNQNNQNNNNN--------------------

D.rerio (XP_696355): ------------------------------------------------------------

D.melanogaster (AFB77909): VDDDG---------------DTDSIPSRMSVRRPEQLQPQ--------------------

M.musculus (NP_001032375): ------------------------------------------------------------

R.norvegicus (NP_001070668): ------------------------------------------------------------

B.taurus (XP_002694905): ------------------------------------------------------------

C.lupus (XP_546782): ------------------------------------------------------------

A.thaliana (NP_182327): -------TKL----------DYDA------------------------------------

O.sativa (NP_001172359): SRVFVVKSSLNRDYVMKINLRWDQIEQTIEILR---------------------------

hPiezo1 (NP_001136336): ----------

hPiezo2 (NP_071351): ----------

E.histolytica (XP_649449): ----------

E.histolytica (XP_655549): ----------

L.major (XP_001686914): LAHGG-----

L.infantum (XP_001469682): PAQGG-----

T.cruzi (XP_819187): VNAAGHEKKE

T.cruzi (XP_812333): VNAAGHYKKE

L.major (XP_001686223): ----------

L.infantum (XP_001468509): ----------

L.braziliensis (XP_001564414): ----------

T.cruzi (XP_817508): ----------

T.cruzi (XP_820998): ----------

T.vaginalis (XP_001319509): ----------

T.vaginalis (XP_001582897): ----------

T.vaginalis (XP_001581503): ----------

T.vaginalis (XP_001580012): ----------

T.vaginalis (XP_001305124): ----------

N.gruberi (XP_002682879): ----------

D.discoideum (XP_640187): -NNNNHEKIN

D.rerio (XP_696355): ----------

D.melanogaster (AFB77909): -QPQ------

M.musculus (NP_001032375): ----------

R.norvegicus (NP_001070668): ----------

B.taurus (XP_002694905): ----------

C.lupus (XP_546782): ----------

A.thaliana (NP_182327): ----------

O.sativa (NP_001172359): --------IN
